# Supplementary material for: Valorization of various lignocellulosic wastes to Ganoderma lucidum (Curtis) P. Karst (Reishi Mushroom) cultivation and their FT-IR assessments
Source: PLoS One. 2025 Jul 22;20(7):e0328732. doi: 10.1371/journal.pone.0328732 (PMC12282901; doi:10.1371/journal.pone.0328732)

PLOS ONE

Valorization of Various Lignocellulosic Wastes to Ganoderma lucidum (Reishi Mushroom) Cultivation and Their FT-IR Assessments

--Manuscript Draft--

**Manuscript Number:**	PONE-D-25-12794
**Article Type:**	Research Article
**Full Title:**	Valorization of Various Lignocellulosic Wastes to Ganoderma lucidum (Reishi Mushroom) Cultivation and Their FT-IR Assessments
**Short Title:**	Valorization of Various Lignocellulosic Wastes to Ganoderma lucidum (Reishi Mushroom) Cultivation
**Corresponding Author:**	RECAİ ARSLAN, Ph.D<br>Düzce Üniversitesi: Duzce Universitesi<br>Düzce, TÜRKIYE
**Keywords:**	Reishi; Ganoderma lucidum; Lignocellulosic wastes; Waste recovery; Biodegradation; FT-IR
**Abstract:**	This study explores the valorization of various lignocellulosic wastes for cultivating Reishi (Ganoderma lucidum) mushrooms, highlighting its potential contributions to sustainable agriculture and waste management. G. lucidum has significant pharmacological benefits, and optimizing its cultivation on diverse substrates enhances its commercial viability. In this research, mushrooms were cultivated using hazelnut branches (HB), hazelnut husk (HH), wheat straw (WS), rhododendron branches (RD), oak wood (OW), beech wood (BW), corn husk (CH), and spent coffee grounds (CG). The biodegradation properties of the mushrooms on the selected substrates were also investigated. HB and RD materials were used for the first time to cultivate and assess the biodegradation properties of G. lucidum. Composts were prepared for cultivation at varying compositions (91% substrate + 9% wheat bran (WB) and 75% HB + 25% other substrates). The nutritional properties of the mushrooms and substrates, the chemical composition (holocellulose, alpha cellulose, extractives, and ash) of the substrates, and Fourier Transform Infrared Spectroscopy (FT-IR) assessments before and after cultivation were analyzed. Among the substrates, OW 91% + WB 9% yielded the highest mushroom production (46 g/kg), whereas CH (18.3 g/kg) resulted in the lowest yield, with no significant difference compared to WS (18.5 g/kg). Following mushroom cultivation, the holocellulose content and pH values of the substrates decreased proportionally, while alpha-cellulose, extractives, and ash content increased. FT-IR spectra indicated that this method could effectively elucidate the biodegradation properties of G. lucidum on lignocellulosic materials before and after cultivation.
**Order of Authors:**	Caglar Akcay
	RECAİ ARSLAN, Ph.D
	Faik Ceylan
**Additional Information:**	
**Question**	**Response**
**Financial Disclosure**<br><br>Enter a financial disclosure statement that describes the sources of funding for the work included in this submission. Review the submission guidelines for detailed requirements. View published research articles from *PLOS ONE* for specific examples.	Yes

Powered by Editorial Manager® and ProduXion Manager® from Aries Systems Corporation

This statement is required for submission and **will appear in the published article** if the submission is accepted. Please make sure it is accurate.<br><br>**Funded studies**<br>Enter a statement with the following details:<br>• Initials of the authors who received each award<br>• Grant numbers awarded to each author<br>• The full name of each funder<br>• URL of each funder website<br>• Did the sponsors or funders play any role in the study design, data collection and analysis, decision to publish, or preparation of the manuscript?<br><br>Did you receive funding for this work?	
Please add funding details.<br>as follow-up to "**Financial Disclosure**<br><br>Enter a financial disclosure statement that describes the sources of funding for the work included in this submission. Review the submission guidelines for detailed requirements. View published research articles from *PLOS ONE* for specific examples.<br><br>This statement is required for submission and **will appear in the published article** if the submission is accepted. Please make sure it is accurate.<br><br>**Funded studies**<br>Enter a statement with the following details:<br>• Initials of the authors who received each award<br>• Grant numbers awarded to each author<br>• The full name of each funder<br>• URL of each funder website<br>• Did the sponsors or funders play any role in the study design, data collection and analysis, decision to publish, or preparation of the manuscript?<br><br>Did you receive funding for this work?"	This research was financially supported by Directory of Scientific Research Projects of Düzce University (project number 2024.29.01.1446).

Please select the country of your main research funder (please select carefully as in some cases this is used in fee calculation).<br>as follow-up to "**Financial Disclosure**<br><br>Enter a financial disclosure statement that describes the sources of funding for the work included in this submission. Review the submission guidelines for detailed requirements. View published research articles from *PLOS ONE* for specific examples.<br><br>This statement is required for submission and **will appear in the published article** if the submission is accepted. Please make sure it is accurate.<br><br>**Funded studies**<br>Enter a statement with the following details:<br>• Initials of the authors who received each award<br>• Grant numbers awarded to each author<br>• The full name of each funder<br>• URL of each funder website<br>• Did the sponsors or funders play any role in the study design, data collection and analysis, decision to publish, or preparation of the manuscript?<br><br>Did you receive funding for this work?"	TURKEY - TR
**Competing Interests**<br><br>Use the instructions below to enter a competing interest statement for this submission. On behalf of all authors, disclose any competing interests that could be perceived to bias this work—acknowledging all financial support and any other relevant financial or non-financial competing interests.<br><br>This statement is required for submission and **will appear in the published article** if the submission is accepted. Please make sure it is accurate and that any funding	The authors have declared that no competing interests exist.

Powered by Editorial Manager® and ProduXion Manager® from Aries Systems Corporation

sources listed in your Funding Information later in the submission form are also declared in your Financial Disclosure statement.<br><br>View published research articles from *PLOS ONE* for specific examples.<br><br>**NO authors have competing interests**<br><br>Enter: *The authors have declared that no competing interests exist.*<br><br>**Authors with competing interests**<br><br>Enter competing interest details beginning with this statement:<br><br>*I have read the journal's policy and the authors of this manuscript have the following competing interests: [insert competing interests here]*<br><br>* typeset	
**Ethics Statement**<br><br>Enter an ethics statement for this submission. This statement is required if the study involved:<br><br>• Human participants<br>• Human specimens or tissue<br>• Vertebrate animals or cephalopods<br>• Vertebrate embryos or tissues<br>• Field research<br><br>Write "N/A" if the submission does not require an ethics statement.<br><br>General guidance is provided below. Consult the submission guidelines for detailed instructions. **Make sure that all information entered here is included in the Methods section of the manuscript.**	N/A

**Format for specific study types**<br><br>**Human Subject Research (involving human participants and/or tissue)**<br>• Give the name of the institutional review board or ethics committee that approved the study<br>• Include the approval number and/or a statement indicating approval of this research<br>• Indicate the form of consent obtained (written/oral) or the reason that consent was not obtained (e.g. the data were analyzed anonymously)<br><br>**Animal Research (involving vertebrate animals, embryos or tissues)**<br>• Provide the name of the Institutional Animal Care and Use Committee (IACUC) or other relevant ethics board that reviewed the study protocol, and indicate whether they approved this research or granted a formal waiver of ethical approval<br>• Include an approval number if one was obtained<br>• If the study involved *non-human primates*, add *additional details* about animal welfare and steps taken to ameliorate suffering<br>• If anesthesia, euthanasia, or any kind of animal sacrifice is part of the study, include briefly which substances and/or methods were applied<br><br>**Field Research**<br><br>Include the following details if this study involves the collection of plant, animal, or other materials from a natural setting:<br>• Field permit number<br>• Name of the institution or relevant body that granted permission	
**Data Availability**<br><br>Authors are required to make all data underlying the findings described fully available, without restriction, and from the time of publication. PLOS allows rare exceptions to address legal and ethical concerns. See the PLOS Data Policy and FAQ for detailed information.	Yes - all data are fully available without restriction

A Data Availability Statement describing where the data can be found is required at submission. Your answers to this question constitute the Data Availability Statement and **will be published in the article**, if accepted.<br><br>**Important:** Stating 'data available on request from the author' is not sufficient. If your data are only available upon request, select 'No' for the first question and explain your exceptional situation in the text box.<br><br>Do the authors confirm that all data underlying the findings described in their manuscript are fully available without restriction?	
**Describe where the data may be found in full sentences. If you are copying our sample text, replace any instances of XXX with the appropriate details.**<br><br>• If the data are **held or will be held in a public repository**, include URLs, accession numbers or DOIs. If this information will only be available after acceptance, indicate this by ticking the box below. For example: *All XXX files are available from the XXX database (accession number(s) XXX, XXX.*).<br>• If the data are all contained **within the manuscript and/or Supporting Information files**, enter the following: *All relevant data are within the manuscript and its Supporting Information files.*<br>• If neither of these applies but you are able to provide **details of access elsewhere**, with or without limitations, please do so. For example:<br><br>*Data cannot be shared publicly because of [XXX]. Data are available from the XXX Institutional Data Access / Ethics Committee (contact via XXX) for researchers who meet the criteria for access to confidential data.*<br><br>*The data underlying the results presented in the study are available from (include the name of the third party*	N/A

and contact information or URL).<br>• This text is appropriate if the data are owned by a third party and authors do not have permission to share the data.<br><br>* typeset	
Additional data availability information:	

Manuscript Click here to access/download;Manuscript;Manuscript.docx

Valorization of Various Lignocellulosic Wastes to *Ganoderma lucidum* (Reishi Mushroom) Cultivation and Their FT-IR Assessments

Caglar Akcay[1,2] , Recai Arslan[2,*] , Faik Ceylan[2]

[1] Düzce University, Forestry Vocational School, Department of Forestry, Konuralp Campus, Düzce, Türkiye

[2] Düzce University, Recycling of Agricultural Wastes to Industry Application and Research Center (DUTAGAM), Konuralp Campus, Düzce, Türkiye

*Corresponding author: recaiarslan@duzce.edu.tr (RA)

Abstract

This study explores the valorization of various lignocellulosic wastes for cultivating Reishi (*Ganoderma lucidum*) mushrooms, highlighting its potential contributions to sustainable agriculture and waste management. *G. lucidum* has significant pharmacological benefits, and optimizing its cultivation on diverse substrates enhances its commercial viability. In this research, mushrooms were cultivated using hazelnut branches (HB), hazelnut husk (HH), wheat straw (WS), rhododendron branches (RD), oak wood (OW), beech wood (BW), corn husk (CH), and spent coffee grounds (CG). The biodegradation properties of the mushrooms on the selected

substrates were also investigated. HB and RD materials were used for the first time to cultivate and assess the biodegradation properties of *G. lucidum*. Composts were prepared for cultivation at varying compositions (91% substrate + 9% wheat bran (WB) and 75% HB + 25% other substrates). The nutritional properties of the mushrooms and substrates, the chemical composition (holocellulose, alpha cellulose, extractives, and ash) of the substrates, and Fourier Transform Infrared Spectroscopy (FT-IR) assessments before and after cultivation were analyzed. Among the substrates, OW 91% + WB 9% yielded the highest mushroom production (46 g/kg), whereas CH (18.3 g/kg) resulted in the lowest yield, with no significant difference compared to WS (18.5 g/kg). Following mushroom cultivation, the holocellulose content and pH values of the substrates decreased proportionally, while alpha-cellulose, extractives, and ash content increased. FT-IR spectra indicated that this method could effectively elucidate the biodegradation properties of *G. lucidum* on lignocellulosic materials before and after cultivation.

Keywords: Reishi, *Ganoderma lucidum*, Lignocellulosic wastes, Waste recovery, Biodegradation, FT-IR

1. Introduction

G. lucidum (Reishi), also known as Ling Chu, Ling Zhi, or Mannetake in China, Korea, and Japan, is a medicinal mushroom that has been widely used in traditional medicine for approximately 2000 years in Eastern countries [1,2]. It is renowned for its pharmacological activities, including antiviral, antibacterial, antifungal, anticancer/cytotoxic, anti-aging, antiallergic, immunomodulating, antidepressant, anti-inflammatory, antioxidative, antihyperlipidemic, antidiabetic, anti-ulcer, hepatoprotective, neuroprotective, nephroprotective,

osteoprotective, and hypotensive effects [3,4]. Due to its extensive medicinal properties, *G. lucidum* has been traditionally believed to contribute to longevity. It has been reported to be effective in treating various diseases, such as cancer, hepatitis, allergies, bronchitis, gastric ulcers, arthritis, chronic hepatitis, hyperglycemia, hypertension, insomnia, and inflammation [3].

Today, several commercial products derived from *G. lucidum* are available on the market in various forms, including powder, capsules, tablets, coffee, tea, syrups, beverages, nutritional supplements, lotions, and toothpaste. Thousands of tons of *G. lucidum* are produced and consumed globally, with China being the largest producer, accounting for approximately 70% of the world's total production [5,6]. The global annual production of *G. lucidum* is estimated to be around 4700 tons, with 3700 tons produced in China alone [6].

G. lucidum is classified as a white-rot fungus due to its ability to degrade lignocellulosic materials efficiently. It plays a significant role in the decomposition and recycling of lignocellulosic compounds in nature. Consequently, it is cultivated on various agricultural and forestry residues, which serve as suitable substrates for its growth [2]. Globally, approximately 998 million tons of agricultural waste are generated annually [7]. Lignocellulosic wastes, primarily composed of cellulose, hemicellulose, and lignin, can be utilized as substrates for mushroom cultivation. Despite an estimated 6×10^9 tons of lignocellulosic waste being produced worldwide each year, only 20% is converted into value-added products such as energy or food [8]. Agricultural and lignocellulosic wastes originate from industries such as pulp and paper, food processing, agriculture, and forestry. In many regions, these wastes are often burned after harvesting, contributing to environmental pollution [9]. Various methods have been explored to mitigate this issue, and mushroom cultivation has emerged as one of the most environmentally friendly approaches for converting lignocellulosic materials into high-value products.

A wide range of lignocellulosic materials have been investigated for the cultivation of edible and medicinal *G. lucidum.* Previous studies have evaluated substrates such as tea waste, eggshells, olive oil extraction residues, soybean meal, date palm leaves, wheat bran, banana peels, sugarcane bagasse, and coffee grounds [10–12]. The choice of substrate significantly affects the nutritional composition, including macro- and microelements, and bioactive compounds of the cultivated mushrooms [2]. However, the availability and cost of these lignocellulosic materials vary across different countries and regions [13]. Hazelnut branch pruning waste is generated during the hazelnut harvest season as a renewable resource, yet it is not widely utilized in industrial applications. In Türkiye, local farmers commonly burn these residues for heating. It has been reported that 1.7 million tons of hazelnut branch pruning waste are generated annually from 0.65 million tons of hazelnut production [14,15]. Since hazelnut branches contain high amounts of cellulose, hemicellulose, and lignin, they hold potential as an alternative substrate for mushroom cultivation. Hazelnut branches have been previously used to cultivate oyster mushrooms (*Pleurotus ostreatus*) [16]. However, no prior research has been conducted on the cultivation and biodegradation properties of *G. lucidum* using hazelnut branches and rhododendron waste as substrates.

This study aimed to cultivate the medicinal mushroom *G. lucidum* on various lignocellulosic wastes, including hazelnut branches, hazelnut husks, wheat straw, rhododendron branches, oak wood, beech wood, corn husks, and spent coffee grounds. Additionally, the biodegradation properties of *G. lucidum* were assessed through chemical analysis and Fourier Transform Infrared Spectroscopy (FT-IR) after cultivation.

2. Material and Method

2.1. Preparation of Composts from Wastes Materials

In this study, hazelnut branches (*Corylus avellana L.)*, hazelnut husks, wheat straw, spent coffee grounds, Rhododendron sp., beech wood (*Fagus orientalis*), oak wood (*Quercus sp.)*, and corn husk wastes were used as substrates for *G. lucidum* cultivation. Hazelnut branches (HB), hazelnut husks (HH), corn husks (CH), *Rhododendron sp.*, and wheat straw (WS) were obtained from local growers, while beech wood (*Fagus orientalis*) and oak wood (*Quercus sp.*) sawdust were sourced from wood processing factories. Spent coffee grounds (CG) were collected from a local coffee company in Düzce, Türkiye.

Italicize

The substrates were processed into suitable sizes: HB and HH were ground using a Wiley mill to 1 cm, *Rhododendron sp.* was cut to 2 cm, WS was prepared at a size of 5–6 cm, and CG was sieved to 0.3–0.5 mm. Beech and oak wood sawdust were sieved to 1.4 mm before use.

How composting was done? For how long the composting process done? How C: N ratio was maintained? This step is fundamentally crucial and needs explanation!

A [illegible] gnocellulosic materials in homogen [illegible] were used as the main component. The mixtures were wetted until their moisture content reached approximately 65% [10]. Each combination was weighed to 1 kg, and 1% $CaCO_3$ was added to adjust the pH. The mixtures were then placed into autoclavable polypropylene bags (28 × 42 cm) and sterilized in an autoclave at 121°C and 1.1 atm pressure for 90 minutes. At least six replicates were prepared for each combination. After sterilization, the pH and moisture content of each mixture were measured, and the composts were allowed to cool to 24°C before inoculation.

The *G. lucidum* spawn used in the study was obtained from the Atatürk Horticultural Central Research Institute, Yalova, Türkiye. The spawn was inoculated into the compost at a rate of 3% (w/w) of the dry compost weight insi [illegible]. The inoculated composts were mixed homogeneously, tightly sealed, and incubated in a dark environment.

For how long??

2.2. Incubation and Mushroom Harvesting

The inoculated composts were transferred to a dark inc[illegible] and 70% relative humidity. Once full mycelial col[illegible] temperature was increased to 28–30°C, and the humidity was adjusted to 80–90%. Carbon dioxide (CO_2) levels in the chamber were maintained below 1000 ppm.

How was CO2 level maintained? For how long the inoculated stubstrate incubated at 24oC?

When primordium/pin formation appeared on the myceliu[illegible] introduced to the incubation chamber to stimulate fruit body deve[illegible] bodies were harvested and weighed using a precision scale.

How the intensity of light was monitored? Was there casing material? If yes, what medium was used as casing material?

A total of three harvesting cycles w[illegible]ach compost combination. The mushroom yield and biological efficiency (B[illegible]sing the following formulas:

What other growth/yield parameters were measured ?

Yield (g/kg) = (Total weight of fresh mushrooms harvested g/1 kg substrate) (1)

BE (%) = (Total weight of fresh mushrooms harvested/ dry matter content of the substrate) × 100 (2)

Table 1. Substrate codes and ratios used in the study.

**Substrates**	**Substrate code**	**Substra[illegible]**
Hazelnut Branch + Wheat Bran	HB+WB	9[illegible]
Rhododendron + Wheat Bran	RD+ WB	9[illegible]
Beech Wood + Wheat Bran	BW +WB	9[illegible]
Oak Wood + Wheat Bran	OW+ WB	9[illegible]
Hazelnut Husk + Wheat Bran	HH +WB	91 [illegible]
Coffee Ground + Wheat Bran	CG +WB	91 + 9

Why the proportion was kept constant at 91:9/75:25? Why not 70:30/65:35/45:55 or 25:75 or other proportions ????? Actually, many possible combinations could have been done!! This is a serious limitation/gap in this work!

Wheat Straw + Wheat Bran	WS+ WB	91 + 9
Corn Husk + Wheat Bran	CH+ WB	91 + 9
Hazelnut Branch+ Hazelnut Husk	HB + HH	75 + 25
Hazelnut Branch+ Rhododendron	HB + RD	75 + 25
Hazelnut Branch + Wheat Straw	HB + WS	75 + 25
Hazelnut Branch + Beech Wood	HB + BW	75 + 25
Hazelnut Branch + Oak Wood	HB + OW	75 + 25
Hazelnut Branch + Coffee Ground	HB + CG	75 + 25
Hazelnut Branch + Corn Husk	HB + CH	75 + 25

2.3. Chemical Analysis of the Substrates

Extractives [17], ho[illegible]ntent and pH [20] values of the substrates were dete[illegible]eferences described by previous

For which substrate the analysis was done (shredded ones? composted ones?). Clarification is needed for this experiment!

2.4. Proximate composition of fruiting bodies

1

2.4. Total Phenolic Contents

The total phenolic content (TPC) of *G. lucidum* fruit bodies was determined using the Folin-Ciocalteu method [21]. First, 0.5 g of dried mushroom powder was mixed with 10 mL methanol and filtered. Then, 800 µL Folin-Ciocalteu reagent and 1 mL Na_2CO_3 solution were added. The solution was incubated in the dark for 30 minutes, and absorbance values were recorded at 720 nm using a spectrophotometer. The results were expressed as mg gallic acid equivalent per gram (mg GAE/g) of the sample.

2.5. Elemental analysis

Elemental analysis was performed using Inductively Coupled Plasma Optical Emission Spectrometry (ICP-OES), following the ISO 22036 (2024) [22] standard protocol for environmental solid matrices. Certified reference materials (Certipur, Merck, Germany) were used for ICP-OES calibration. Prior to analysis, mushroom compost and fruit body samples were digested in a microwave oven using a [illegible]drogen peroxide (H_2O_2).

Why only phenol and metals? What about other minerals and nutrients??

2.6. FT-IR Measurements

FT-IR analysis was conducted at the Düzce University Scientific and Technological Research Laboratory. The substrate samples were first ground and dried at 103 ± 2°C for 12 hours. FT-IR spectra were recorded in the fingerprint region (600–1800 cm^{-1}) using a resolution of 4 cm^{-1} for 32 scans.

2.7. Statically assessments

Statistical analyses were carried out using SPSS [illegible]orp., Armonk, NY, USA). The averages were evaluated using a one-way analysis of variance (ANOVA). Duncan's multiple range test was applied at the level of α= 0.005 to determine the significant differences among the paired comparisons (compost contents and mushroom quality properties).

This is too old version! Why not version 28 or beyond?

3. Results and discussion

Was there procedures for spawn run in the Materials and Methods section? What ingredients were used in spawn preparation? If not, how this is presented in the Results section??

[illegible]arliness, Yield, Biological Efficiency and Dry Matter

Spawn run time, earliness (days to first harvest), yield, BE and dry matter values of the substrates used for the mushrooms harvested in the study were given Table 2. All tested substrates were successfully colonized by *G. lucidum* mycelium; however, significant differences ($p < 0.05$) were observed in the spawn run time across the different substrates. The shortest spawn run time (5 days) was recorded in the RD 91% + WB 9% mixture, while the longest colonization period (14 days) was observed in HH 91% + WB 9% and CH 91% + WB 9% substrates.

Some studies have used oak wood (OW) as a basal substrate or control for the cultivation of *G. lucidum*. The spawn run time for OW was found to be 7 days in the current study, which was shorter than the spawn run time (18 days) reported by Atila, Ogutcu [2]. The mycelium used, the species of oak wood, and the environmental conditions can be considered factors affecting the spawn run time. The longer spawn run time in HH 91% + WB 9% substrates may be related to the high nitrogen or lignin content in the HH material [16].

Significant differences were found between mean earliness values across substrates ($p<0.05$). The lowest earliness (43 days) was detected in the OW 91% + WB 9% substrate. The earliness value for OW was much shorter than the reported values by Atila, Ogutcu [2], Gurung, Budathoki [23], and Roy, Jahan [24]. HB material was used for the first time in this study for cultivation with various agricultural wastes, and its mean earliness was found to be 47.2 days, which was not significantly different from OW (43 days), RD (43.5 days), and BW (51 days). When other agricultural wastes were supplemented by 25% to the HB, the mean earliness did not significantly differ ($p<0.05$). Although all substrates were colonized with mycelium, primordia

p=?? Wrong p value was given for the absence of significant difference!

Could this be considered the highest yield?

and fruit bodies did not occur in the HH 91% + WB 9%, CG 91% + WB 9%, and HB 75% + CG 25% substrates.

When yield values were examined, the highest yield (46 g/kg) was achieved in the substrate prepared from OW 91% + WB 9% (significantly different from the other substrates, $p<0.05$), while the lowest yield was found in the substrates prepared with WS 91% + WB 9% and CH 91% + WB 9%, at 18.8 and 18.5 g/kg, respectively. Since no fruit body formation occurred in the substrates prepared with HH 91% + WB 9%, CG 91% + WB 9%, and HB 75% + CG 25%, the yield value and BE were not calculated. HH and CG materials were reported by Akcay, Ceylan [16] and Ozcelik and Peksen [25] as suitable cultivation materials for *Pleurotus ostreatus* (Oyster mushroom) and *Lentinus edodes* (Shiitake), both of which are edible and medicinal mushroom species. However, some studies have reported that increasing the level of spent coffee grounds in sawdust substrates delayed mycelium growth and prevented fruiting during mushroom cultivation Carrasco-Cabrera, Bell [26]. A similar issue occurred in the cultivation of *G. lucidum* in the current study. The yield of the substrate prepared with HB material, which was used for the first time in *G. lucidum* cultivation, was 28.3 g/kg. The RD material, also used for the first time, had a yield of 36.3 g/kg. When HB and RD materials were mixed with other lignocellulosic materials at a ratio of 25%, the yield values were not significantly different ($p<0.05$). It seems that the supplementation of lignocellulosic materials (at 25%) to HB did not negatively affect the yield. Thus, the lignocellulosic materials used in this study could be promising alternative substrates for *G. lucidum* cultivation. When comparing our results with previous studies, the yield determined for OW 91% + WB 9% (46 g/kg) in the current study was similar to the results reported by Yakupoglu [27] and Peksen and Yakupoglu [10], who investigated the yields of substrates prepared by mixing sawdust and wood chips of oak and hornbeam with tea wastes (42 g/kg). However, the yield values of other substrates were lower than those in their results across

all combinations. The differences and lower yields could be attributed to variations in substrate combinations, pH values, and cultivation conditions. BE values showed similar trends to yield values. The highest BE value (9.20%) was found in substrates prepared with OW 91% + WB 9%, while the lowest (5.39%) was found in the BW 91% + WB 9% substrate.

Significant differences were detected between substrates in terms of dry matter in mushroom fruit bodies ($p<0.05$). The highest dry matter content was found to be 28.2%, while the lowest was 22.6%, among those grown on different lignocellulosic materials at a ratio of 91% and WB 9%. When dry matter values from the HB 75% and different lignocellulosic material substrates with 25% were examined, the highest dry matter (32.09%) was achieved in the HB 75% + HH 25% substrate. Dry matter content in *G. lucidum* mushroom fruit bodies is an important characteristic, as it is valued when dried in the market [5].

Table 2. Spawn run time, earliness, yield, biological efficiency and dry matter of mushroom fruit body.

Substrates	Spawn run time (days)	First promordium (days)	Earliness (days)	Yield g/kg	BE (%)	Dry matter %
HB 91% + 9% WB	7^{b}	21.7^{abc}	47.2^{ab}	28.3^{bc}	5.65^{ab}	28.2^{bc}
RD 91% + 9% WB	5^{a}	21^{ab}	43.5^{a}	36.3^{c}	7.25^{ab}	27.0^{b}
BW 91% + 9% WB	11^{c}	23.42^{cd}	51^{bc}	27^{b}	5.39^{a}	26.4^{b}
OW 91% + 9% WB	7^{b}	21.1^{ab}	43^{a}	46^{d}	9.20^{c}	22.6^{a}
HH 91% + 9% WB	14^{d}	*	*	*	*	*
CG 91% + 9% WB	7^{b}	*	*	*	*	*
WS 91% + 9% WB	11^{c}	23.6^{cd}	53^{cd}	18.8^{a}	7.54^{bc}	27.10^{b}

CH 91% + 9% WB	14^{d}	24.6^{e}	55.1^{d}	18.5^{a}	7.4^{bc}	32.03^{de}
HB 75 % + HH 25%	7^{b}	24.1^{e}	49.4^{bc}	29.4^{bc}	5.88^{ab}	32.09^{e}
HB 75 % + RD 25%	7^{b}	20.4^{a}	44.8^{a}	33.4^{bc}	6.68^{ab}	26.56^{b}
HB 75 % + WS 25%	8^{c}	20.5^{a}	49.1^{bc}	29.2^{bc}	5.85^{ab}	29.64^{bcd}
HB 75 % + BW 25%	7^{b}	23.1^{bcd}	51.8^{cd}	34.3^{bc}	6.86^{ab}	30.88^{cde}
HB 75 % + OW 25%	7^{b}	20.8^{ab}	49.8^{bc}	28.5^{bc}	5.70^{ab}	29.41^{bcd}
HB 75 % + CG 25%	7^{b}	*	*	*	*	*
HB 75 % + CH 25%	[illegible]	[illegible]	[illegible]	[illegible]	6.81^{ab}	29.20^{bcd}

*not occurred mush[illegible]

Did the author/s/ have procedures for OC or nitrogen or C:N ratio determination? If not, why it is mentioned here?? The work has a fundamental problem!

3.2. Total Organic Carbon (TOC), Phenolic Content (TPC) and Elemental Composition

The total organic carbon (TOC), nitrogen (N), C/N ratio, and phenolic content of the substrates, as well as the phenolic content of the cultivated mushrooms, are shown in Table 3. When examining Table 3, the highest TOC was found in RD at 21.89%, while the lowest was in HH. However, TOC values were generally similar. When the nitrogen contents of the substrates were analyzed, the highest N content was observed in CG (2.52%), followed by HH (1.51%). The lowest nitrogen content was found in OW and BW (0.41% and 0.44%, respectively). The C/N ratio is a critically important indicator in mushroom production. In this study, the lowest C/N ratios were found in CG and HH substrates. It can be suggested that the low C/N ratio in these substrates plays an active role in the formation of mushroom caps. The highest C/N ratio was observed in the OW substrate at 50.68%, followed by RD and BW. Yield values were also high in substrates with high C/N ratios. Similar results were reported by Peksen and Yakupoglu [10],

who found a strong positive correlation between the C/N ratio of substrates and mushroom yield. Atila [28] also found that yield was strongly positively correlated with C/N ratio, cellulose, and lignin content, while it was negatively correlated with the nitrogen content of the substrates.

When the total phenolic content (TPC) was examined, the highest TPC among the substrates was found in RD, with a value of 14.872 mg GAE/g. The highest TPC in mushroom fruit bodies was recorded in BW at 3.156 mg GAE/g, while the lowest TPC among mushroom fruit bodies was found in mushrooms cultivated in WS, with a value of 1.687 mg GAE/g. Modi, Shah [29] reported that the TPC values of *G. lucidum* extracts collected from nature varied between 33.833 and 83.674 mg/g dry extract, depending on the solvent used in their study. Atila, Ogutcu [2] found TPC values ranging from 20.36 to 22.60 mg GAE/g in *G. lucidum* mushrooms cultivated on various phenolic-rich forest and agro-food waste substrates. Similarly, Demirci, Sözeri [30] reported that the TPC values of five different commercially available *G. lucidum* samples ranged between 2.35 and 10.46 mg GAE/g. It appears that the TPC values of the mushrooms in our study were considerably lower than those reported by Modi, Shah [29] and Atila, Ogutcu [2], but similar to the values reported by Demirci, Sözeri [30]. The TPC values of mushrooms can vary depending on the solvent used for extraction, such as methanol, ethanol, hexane, or ether.

Table 3. Total organic carbon and phenolic content of raw materials and fruit body cultivated.

**Substrate type**	**Average TOC (%)**	**N**	**C/N**	**Total phenolic content (mg GAE/g sample)**	
	**Substrates**	**Substrates**	**Substrates**	**Substrates**	**Fruit body**

**HB**	20.02 ± 0.110^{ab}	1.23 ± 0.07^{a}	16,27 ± 0.97^{ab}	1.751 ± 0.001^{a}	2.191 ± 0.001^{a}
**RD**	21.82 ± 0.445^{d}	0.5 ± 0.08^{b}	43,64 ± 4.1^{d}	14.872 ± 0.001^{b}	2.619 ± 0.000^{b}
**BW**	19.77 ± 0.790^{ab}	0.44 ± 0.04 ^{b}	44,93 ± 9.8^{d}	14.083 ± 0.003^{c}	3.156 ± 0.005^{c}
**OW**	20.78 ± 0.160^{c}	0.41 ± 0.08^{b}	50,68 ± 5.4^{d}	2.083 ± 0.001^{d}	2.689 ± 0.000^{d}
**HH**	19.40 ± 0.065^{a}	1.51 ± 0.08^{c}	12,84 ± 0.66^{a}	4.423 ± 0.005^{e}	*
**CG**	21.69 ± 0.145^{d}	2.52 ± 0.11^{d}	8,60 ± 0.35^{a}	3.199 ± 0.001^{f}	*
**WS**	20.00 ± 0.510^{ab}	0.68 ± 0.06^{e}	29,41 ± 4.5^{c}	2.513 ± 0.001^{g}	1.687 ± 0.001^{e}
**CH**	20.20 ± 0.375^{bc}	0.83 ± 0.06^{f}	24,33 ± 1.6^{bc}	1.787 ± 0.001^{h}	2.909 ± 0.005^{f}

[*]Mushroom fruit body was not occurred

The elemental analysis of substrates and cultivated mushroom fruit bodies in this study is presented in Table 4. Upon examining Table 4, significant differences were observed among the substrates in terms of mineral composition. Essential minerals such as Fe, Cu, Zn, Mn, Mg, and Ca are required for fungal growth. Peksen and Yakupoglu [10] found that yield and biological efficiency (BE) were positively and significantly correlated with K, Fe, and Mn in *G. lucidum* production. Similarly, Ozcelik and Peksen [25] reported that the P and Zn content of substrates was strongly correlated with yield and BE in *Lentinus edodes* cultivation. When the mineral contents of the cultivated mushroom fruit bodies were analyzed, significant differences were observed. The levels of Fe, Cu, Mg, K, and Ca were higher in OW-FB fruit bodies compared to those grown on other substrates. On the other hand, Zn, Mn, and Na levels were higher in CH-FB fruit bodies than in others.

Table 4. Elemental analysis of substrates and fruit bodies cultivated.

Sample	N (wt%)	P (wt%)	Fe (mg/kg)	Cu (mg/kg)	Zn (mg/kg)	Mn (mg/kg)	Na (mg/kg)	Mg (mg/kg)	K (mg/kg)	Ca (mg/kg)
HB	1.23	0.1	80.4	38.6	33.3	28.6	159.1	2006.0	8531.8	1468.9
RD	0.5	0.0	87.2	31.9	41.4	133.6	229.5	1382.7	12575.0	1570.0
BW	0.44	0.0	217.8	57.7	4.2	88.2	239.5	1649.9	10282.1	2470.0
OW	0.41	0.0	117.2	299.1	2.4	48.9	133.6	554.3	1043.0	2896.3
HH	1.51	0.1	145.8	10.6	43.7	65.4	172.1	884.6	7917.6	971.2
CG	2.52	0.1	150.1	55.6	3.5	19.5	167.0	746.9	5128.8	5479.6
WS	0.68	0.0	95.6	7.6	95.7	155.3	232.5	1269.4	14708.5	616.6
CH	0.83	0.1	186.0	10.8	74.6	138.6	359.5	1647.4	19819.3	782.3
HB-FB*	2.9	0.4	148.6	35.5	3.0	31.2	119.8	116.9	996.5	6106.7
RD (FB)	3.77	0.5	90.7	264.0	13.3	36.8	274.5	914.6	9098.1	5385.9
BW (FB)	3.71	0.3	101.2	15.1	68.3	131.1	277.7	1272.9	13907.3	993.4
OW (FB)	3.58	0.4	1351.2	1207.2	14.1	65.9	246.9	1953.0	22297.6	12172.6
HH (FB)**										
CG (FB)**										
WS (FB)	3.92	0.5	91.3	79.9	2.0	38.4	202.8	457.5	1328.4	4992.0
CH (FB)	3.5	0.5	153.4	14.6	91.9	168.6	300.8	1412.9	20094.3	735.5

What is this??

HB-FB*Fruit body (FB) cultivated from hazelnut branches (HB), the others were coded similarly

**Mushroom fruit body was not occurred

3.3. FT-IR Spectroscopy and Biodegradation Properties

The FT-IR spectra in the fingerprint region (1800-600 cm^{-1}) showing the changes in the structure after the fungal attack of hazelnut branch (HB), beech sawdust (BS), oak sawdust (OS), rhododendron sawdust (RS), corn stalk (CS), wheat straw (WS), hazelnut husk (HH), and coffee ground (CG) compared to the control (before fungal attack) are given in Figs 1-8. The bands corresponding to vibrations in this region, as well as the changes in band intensities of the samples at different wavenumbers following fungal attack, are summarized in Table 5.

Fig 1. FT-IR results. FT-IR spectra of HB-C and HB-F before and after fungal degradation.

Fig 2. FT-IR results. FT-IR spectra of BW-C and BW-F before and after fungal degradation.

Fig 3. FT-IR results. FT-IR spectra of OW-C and OW-F before and after fungal degradation.

Fig 4. FT-IR results. FT-IR spectra of RD-C and RD-F before and after fungal degradation.

Fig 5. FT-IR results. FT-IR spectra of WS-C and WS-F before and after fungal degradation.

Fig 6. FT-IR results. FT-IR spectra of CS-C and CS-F before and after fungal degradation.

Fig 7. FT-IR results. FT-IR spectra of HH-C and HH-F before and after fungal degradation.

Fig 8. FT-IR results. FT-IR spectra of CG-C and CG-F before and after fungal degradation.

The bands observed between 1750 and 1720 cm^{-1} are attributed to hemicellulose degradation in lignocellulosic materials. This band indicates unconjugated C=O vibrations of acetyl, carboxylic acid, and uronic ester groups within the hemicellulose structure [31,32]. In this study, this band was observed at 1732 cm^{-1} in BW-C and RD-C samples and shifted to 1722 and 1737 cm^{-1}, respectively, after fungal attack. No significant change was observed in this band for HB, OW, WS, CH, and HH samples. The degradation of hemicellulose in RD and CH samples following fungal attack was higher than in other samples, leading to increased band intensity.

The strong band observed at 1741 cm^{-1} in the CG-C sample is attributed to the C=O stretching vibrations of ester groups from kinic acid and lipids [33]. After the fungal attack, this band disappeared. The bands between 1650 and 1590 cm^{-1} correspond to lignin degradation in lignocellulosic materials [31]. In this region, the deformation of O-H groups in the lignin structure (1640 cm^{-1}), C=C and C=O stretching in the lignin aromatic chain (1630 cm^{-1}), and C-O stretching vibrations in the lignin skeletal structure (1630 cm^{-1}) overlapped, forming a broad band [16]. As a result of lignin degradation by *G. lucidum*, the band intensity in this region increased in HB, BW, OW, RD, WS, and HH samples, while no change was observed in WS and CH

samples. This indicates that *G. lucidum* mycelia degrade lignin in perennial plants (HB, BW, OW, and RD) more effectively than in annual plants (CH and WS).

The broad band observed at 1645 cm^{-1} in the CG-C sample is attributed to C=C stretching vibrations of lipids and fatty acids, C=O stretching vibrations of caffeine, and C=C, C=O, and C-O stretching vibrations of the lignin aromatic chain [34]. After the fungal attack, the intensity of this band decreased.

The bands between 1520 and 1500 cm^{-1} correspond to the aromatic C-O stretching vibrations of lignin [35]. Following a fungal attack, band intensity increased in HB, RD, and CH samples, remained unchanged in BW, OW, and WS samples, and decreased in HH and CG samples. In contrast, [16], reported that *Pleurotus ostreatus* increased the visibility of the 1652 cm^{-1} band due to lignin degradation in coffee. This suggests that *G. lucidum* has a weaker effect on lignin in coffee grounds compared to *Pleurotus ostreatus*.

The bands between 1460–1450 cm^{-1} and 1420–1415 cm^{-1} are attributed to C-H deformations of CH_2 and CH_3 groups in lignin and hemicellulose, as well as chlorogenic acids in coffee, and CH_2 in-plane bending vibrations in cellulose and lignin, respectively [36–38]. After the fungal attack, the first band increased in HB, RD, and CH samples, remained unchanged in BW, OW, WS, and HH samples, and decreased in CG samples. The second band increased in HB, BW, OW, RD, and CH samples but decreased in WS, HH, and CG samples. A similar result was observed for CG in *Pleurotus ostreatus* mycelia [16].

The bands between 1375–1365 cm^{-1} and 1325–1315 cm^{-1} correspond to symmetric and asymmetric C-H deformation vibrations of cellulose and hemicellulose, O-H deformation of kinic acid, CH_2 in-plane bending vibrations at the sixth carbon of crystalline cellulose, and C-H deformation of kinic acid, respectively [35,39]. After the fungal attack, the first band increased in HB, BW, OW, RD, and CH samples but decreased in WS, HH, and CG samples. The second

band increased in HB, OW, RD, CH, and HH samples but decreased in WS and CG samples. This result aligns with the increased visibility of C-H vibrations in cellulose, hemicellulose, and lignin observed in the 1420–1415 cm^{-1} band.

The bands between 1245–1230 cm^{-1} correspond to C-O stretching vibrations of hemicellulose, lignin, and kinic acid (in CG samples) [39]. After the fungal attack, this band decreased in HH and CG samples and increased in all other samples.

The bands observed between 1160 and 1145 cm^{-1} and 1035 and 1025 cm^{-1} are attributed to C–O–C vibrations of cellulose and hemicelluloses (glycosidic bonds), as well as quinic acid (CG sample) and C–OH bending, respectively [33,34,40]. After the fungal attack, the intensity of both bands decreased for the HH and CG samples but increased for all other samples. The decrease in the HH and CG samples is similar to the results observed in the 1245–1230 cm^{-1} band, where the visibility of cellulose and hemicellulose was reduced. In the study by Akcay, Ceylan [16], it was observed that the *Pleurotus ostreatus* fungus degraded hazelnut husk and coffee ground materials, facilitating mushroom formation. However, in this study, it was found that the *G. lucidum* fungus did not degrade the hazelnut husk and coffee ground materials.

The weak bands observed between 900 and 700 cm^{-1} are attributed to C_1–O–C_4 β-(1–4) glycosidic bonds [40,41]. Similarly, the intensity of these bands decreased for the HH and CG samples but increased for all other samples. When all the results are evaluated together, *G. lucidum* was found to affect cellulose, hemicellulose, lignin, polysaccharides, and chlorogenic acids (in coffee grounds) in the lignocellulosic structure. *G. lucidum* degraded the HH and CG samples to a lesser extent than the other samples.

Table 5. Assignments of bands between 1800 and 600 cm^{-1} and variations in band intensities of the samples following fungal attack in FT-IR spectra.

Wavenumber (cm^{-1})	Band assignment	Band intensity							
		HB	BS	OS	RS	CS	WS	HH	CG
1750-1720	C=O vibration	(o)	(o)	(o)	(+)	(+)	(o)	(o)	(-)
1650-1590	C=C, C=O, C-O stretching<br>O-H deformation	(+)	(+)	(+)	(+)	(o)	(o)	(+)	(-)
1520-1500	C-O stretching	(+)	(o)	(o)	(+)	(+)	(o)	(-)	(-)
1460-1450	C-H deformation	(+)	(o)	(o)	(+)	(+)	(o)	(o)	(-)
1420-1415	C-H bending	(+)	(+)	(+)	(+)	(+)	(-)	(-)	(-)
1375-1365	C-H, O-H deformation	(+)	(+)	(+)	(+)	(+)	(-)	(-)	(-)
1325-1315	C-H bending<br>C-O deformation	(+)	(o)	(+)	(+)	(+)	(-)	(+)	(-)
1230-1245	C-O stretching<br>C-O-C absorption	(+)	(+)	(+)	(+)	(+)	(+)	(-)	(-)
1160-1145	C-O-C vibration<br>(glycosidic bond)	(+)	(+)	(+)	(+)	(+)	(+)	(-)	(-)
1035-1025	C-O stretching<br>C-OH bending	(+)	(+)	(+)	(+)	(+)	(+)	(-)	(-)
900-700	C_1-O-C_4 glycosidic bond	(+)	(+)	(+)	(+)	(+)	(+)	(-)	(-)

$^{(+)}$increased

$^{(-)}$decreased

$^{(o)}$unchanged

The FT-IR spectra (3600-600 cm^{-1}) of *G. lucidum* mushrooms produced with hazelnut branch (HB), beech sawdust (BS), oak sawdust (OS), rhododendron sawdust (RS), corn stalk (CS), and wheat straw (WS) substrates are given in Fig 9.

Fig 9. FT-IR results. FT-IR spectra of all mushroom samples. HB-M: Mushroom fruit bodies cultivated from HB, other mushroom fruit bodies were coded similarly

The broad bands observed between 3500 and 3000 cm^{-1} are attributed to O–H stretching vibrations due to intermolecular hydrogen bonds and humidity [42,43]. The bands observed at 2922 and 2854 cm^{-1} are attributed to CH_2 asymmetric and symmetric stretching vibrations of lipids in *G. lucidum* mushrooms. These bands are associated with the structural features of triterpenoids, which are characteristic of *G. lucidum* mushrooms [42,44]. Similarly, the two bands observed at 1455 and 1373 cm^{-1} are attributed to CH_3 in-plane and symmetric bending vibrations in the aliphatic structure of triterpene compounds. While these vibrations are primarily attributed to the structural properties of triterpenoids, it has also been suggested that protein and polysaccharide structures can influence these bands [43].

[31] reported that CH_2 bending vibrations in polysaccharides occur at 1455 cm^{-1}, while [42] mentioned that there was the presence of β-glucan in the band at 1373 cm^{-1}. The bands at 1634, 1539, and 1244 cm^{-1} are attributed to amide I (C=O stretching), amide II (C–N, N–H stretching), and amide III (C–N, N–H stretching) in the protein structure of *G. lucidum* mushrooms, respectively [31,44,45].

The bands observed at 1150 and 1030 cm^{-1} are attributed to C-O stretching vibrations of triterpene compounds and β-glucan of polysaccharides in the *G. lucidum,* as well as –OH and C–O–C stretching vibrations of sugar and polysaccharide structures (arabinan) [44]. The weak band observed at 889 cm^{-1} is corresponding to the anomeric configuration of polysaccharides. This band is associated to the presence of 1,3 1,3-β-D-glucan as well as C–H deformation in polysaccharide structures [31].

When all *G. lucidum* mushrooms were examined together, it was observed that the lipid, protein, and polysaccharide components in the structure of the mushroom remained largely unchanged regardless of the substrate type (HB, BW, OW, RD, CH, WS). However, only the band intensities in the FT-IR spectra varied depending on the substrate type. In this study, *G.lucidum* mushroom could not be grown on HH and CG substrates, whereas Akcay, Ceylan [16] observed that *Pleurotus ostreatus* mushrooms successfully grew on both substrates.

3.4. Chemical Analysis of The Substrates Before and After Cultivation

Chemical analysis of the substrates before and after cultivation is shown in Table 6. When Table 6 was examined, it was found that holocellulose and pH values decreased while α–cellulose, extractives, and ash content values proportionately increased compared with previous values after cultivation. Significant differences were found in substrate contents. Among the holocellulose content of the substrates control, the highest amount was found in OW-C (80.71%), while the lowest was in HH-C (47.2%). Since OW contains high holocellulose, it can be said that OW-C gave a high mushroom yield. It can be said that the lack of mushroom fruit body formation in HH material is due to low cellulose content. The decrease in the amount of holocellulose after mushroom cultivation is due to the degradation activity of fungal enzymes of *G. lucidum*. The proportional increase in the amount of α-cellulose after fungal degradation (after mushroom cultivation) shows that it is due to the higher degradation of hemicellulose structures. Fungal enzymes act selectively in lignocellulosic structures, and that causes the structures to degrade at different rates. The proportional increases in the amount of extractive and ash show the proportional increase in the structure as a result of the degradation of organic structures and the

inorganic structures not being degraded. Similar results have been determined in previous studies conducted by Zhang, Li [46], Akcay, Arslan [47], and Akcay, Ceylan [16]. When extractive content was examined it was found that significant differences was detected between substrates ($P<0.05$). The highest extractive content was detected in CG-C (12.08%) substrate while the lowest in OW-C (0.41%) but not statically signifi[illegible]B-C (1.14%).

Table 6. Chemical analysis of the substrates be[illegible]vation.

Substrates	Holocellulose (%)	α –Cellulose (%)	E[illegible]	[illegible]sh (%)	pH
**HB-C**	64.76 ± 1.26^{f}	47.83 ± 1.27^{b}	1.14 ± 0.76^{ab}	2.54 ± 0.27^{d}	7.01 ± 0.11^{h}
**HB-F**	64.49 ± 0.67^{f}	66.42 ± 3.07^{g}	2.99 ± 0.11^{efgh}	3.7 ± 0.12^{f}	4.66 ± 0.07^{bc}
**RD-C**	76.4 ± 0.51^{i}	49.33 ± 0.35^{bc}	3.56 ± 0.54^{fghi}	1.61 ± 0.22^{b}	4.68 ± 0.13^{bc}
**RD-F**	67.06 ± 0.43^{g}	54.58 ± 3.86^{de}	3.64 ± 0.52^{ghi}	2.12 ± 0.15^{c}	4.35 ± 0.06^{a}
**BW-C**	71.35 ± 0.24^{i}	55.48 ± 0.07^{de}	2.70 ± 0.33^{def}	1.65 ± 0.15^{b}	5.03 ± 0.20^{d}
**BW-F**	66.6 ± 0.24^{g}	58.9 ± 0.7^{ef}	3 ± 0.19^{efgh}	4.1 ± 0.16^{f}	4.18 ± 0.20^{a}
**OW-C**	80.71 ± 0.35^{j}	53.68 ± 0.32^{cd}	0.41 ± 0.16^{a}	1.15 ± 0.08^{a}	5.53 ± 0.03^{e}
**OW-F**	69.89 ± 0.76^{h}	55.05 ± 0.85^{de}	1.44 ± 0.59^{bc}	3 ± 0.12^{e}	4.55 ± 0.06^{b}
**HH-C**	47.2 ± 0.32^{a}	49.36 ± 0.42^{bc}	2.56 ± 0.35^{de}	9.37 ± 0.22^{g}	5.94 ± 0.11^{f}
**HH-F**	49.6 ± 0.36^{b}	58.96 ± 8.58^{ef}	4.06 ± 0.85^{ii}	10.5 ± 0.05^{h}	5.41 ± 0.09^{e}
**CG-C**	61.72 ± [illegible]	[illegible]	12.8 ± 0.09^{j}	1.56 ± 0.17^{b}	6.54 ± 0.15^{g}
**CG-F**	51.85 ± 0.40^{c}	37.8 ± 0.39^{a}	2.74 ± 0.19^{def}	2.96 ± 0.21^{e}	5.07 ± 0.06^{d}
**WS-C**	75.32 ± 1.38^{i}	52.93 ± 0.24^{cd}	1.99 ± 0.66^{cd}	4.21 ± 0.29^{g}	7.12 ± 0.12^{h}
**WS-F**	66.76 ± 0.54^{g}	60.48 ± 0.94^{f}	4.12 ± 0.38^{i}	8.55 ± 0.05^{f}	4.76 ± 0.14^{c}
**CH-C**	76.46 ± 0.5^{i}	45.61 ± 0.20^{b}	3.77 ± 0.73^{hi}	3.1 ± 0.66^{e}	5.18 ± 0.10^{d}
**CH-F**	60.4 ± 0.4^{d}	54.2 ± 1.03^{de}	2.84 ± 0.37^{defg}	5.88 ± 0.12^{h}	5.13 ± 0.07^{d}

4. Conclusion

In this study, *G. lucidum* mushroom was cultivated from various agriculture, forest and lignocellulosic waste materials. HB and RD were used for the first time to cultivate *G. lucidum* and to determine its biodegradation properties. At the end of the study, HH and CG wastes did not produce mushroom fruit bodies, although mycelium colonization was completed in these substrates. Among the substrates, the C/N ratio was also found to be lower in HH and CG. The highest yield (46 g/kg) was achieved in the OW substrate, while the lowest yield was recorded in CH (18.3 g/kg), although this was not significantly different from WS (18.5 g/kg). Among the mushroom fruit bodies cultivated from different substrates, the highest TPC in mushroom fruit bodies was found in BW (3.156 mg GAE/g), while the lowest was in WS (1.687 mg GAE/g). The mineral content analysis showed that Fe, Cu, Mg, K, and Ca levels were higher in OW-FB fruit bodies, while Zn, Mn, and Na levels were higher in CH-FB fruit bodies. After cultivation, holocellulose, and pH values of the substrates proportionately decreased, while α-cellulose, extractives, and ash content increased. FT-IR analysis revealed that *G. lucidum* affected cellulose, hemicellulose, lignin, polysaccharides, and chlorogenic acids (in coffee grounds) within the lignocellulosic structure. The degradation of HH and CG substrates by *G. lucidum* was lower compared to other substrates. FT-IR assessments of mushrooms showed that lipid, protein, and polysaccharide components in the mushroom structure remained largely unchanged regardless of substrate type (HB, BW, OW, RD, CH, and WS), with only variations in band intensities in the FT-IR spectra. In conclusion, this study highlights the potential valorization of recycling hazelnut branch, Rhododendron, corn husk, beech wood, and oak wood sawdust waste for the production of *G. lucidum*.

References

1. Unlu A, Nayir E, Kirca O, Ozdogan M. Ganoderma Lucidum (Reishi Mushroom) and cancer. J BUON. 2016;21: 792–798.
2. Atila F, Ogutcu H, Bilginoglu E, Kazankaya A, Kumar P, Fayssal SA. Effect of Phenolic-rich Forest and Agri-food Wastes on Yield, Antioxidant, and Antimicrobial Activities of Ganoderma lucidum. Biomass Convers Biorefinery. 2023;14: 25811–25821. doi:10.1007/s13399-023-04708-6
3. Sliva D. Cellular and Physiological Effects of Ganoderma lucidum (Reishi). Mini-Reviews Med Chem. 2012;4: 873–879. doi:10.2174/1389557043403323
4. Venturella G, Ferraro V, Cirlincione F, Gargano ML. Medicinal mushrooms: Bioactive compounds, use, and clinical trials. Int J Mol Sci. 2021;22: 1–31. doi:10.3390/ijms22020634
5. Lai T, Gao Y, Zhou S. Global Marketing of Medicinal Ling Zhi Mushroom Ganoderma lucidum (W.Curt.:Fr.) Lloyd (Aphyllophoromycetideae) Products and Safety Concerns. Int J Med Mushrooms. 2004;6: 189–194.
6. Li S, Dong C, Wen H, Liu X. Development of Ling-zhi industry in China – emanated from the artificial cultivation in the Institute of Microbiology, Chinese Academy of Sciences (IMCAS). Mycology. 2016;7: 74–80. doi:10.1080/21501203.2016.1171805
7. Khalife E, Sabouri M, Kaveh M, Szymanek M. Recent Advances in the Application of Agricultural Waste in Construction. Appl Sci. 2024;14. doi:10.3390/app14062355
8. Mapayi TT, Oluduro AO, Omoboye OO, Taiwo AM. Medicinal plant assisted cultivation of Pleurotus florida using different lignocellulosic waste substrates. Vegetos. 2021;34: 485–494. doi:10.1007/s42535-021-00225-x
9. Senthilguru K, George TS, Vasanthi NS. Ethanol production from lignocellulosic waste. World J Sci Technol. 2011;1: 12–16.

10. Peksen A, Yakupoglu G. Tea waste as a supplement for the cultivation of Ganoderma lucidum. World J Microbiol Biotechnol. 2009;25: 611–618. doi:10.1007/s11274-008-9931-z

11. Kurd-Anjaraki S, Ramezan D, Ramezani S, Samzadeh-Kermani A, Pirnia M, Shahi BY. Potential of waste reduction of agro-biomasses through Reishi medicinal mushroom (Ganoderma lucidum) production using different substrates and techniques. Acta Ecol Sin. 2022;42: 90–101. doi:10.1016/j.chnaes.2021.04.010

12. Khoo SC, Ma NL, Peng WX, Ng KK, Goh MS, Chen HL, et al. Valorisation of biomass and diaper waste into a sustainable production of the medical mushroom Lingzhi Ganoderma lucidum. Chemosphere. 2022;286: 131477. doi:10.1016/j.chemosphere.2021.131477

13. Yildiz S, Yildiz ÜC, Gezer ED, Temiz A. Some lignocellulosic wastes used as raw material in cultivation of the Pleurotus ostreatus culture mushroom. Process Biochem. 2002;38: 301–306. doi:10.1016/S0032-9592(02)00040-7

14. Alkaya E, Akarsoy Altay T, Başak Atak A, Ölmez Çakar S, Durtaş P. Tarımsal atıklardan yüksek katma değerli biyoürün üretimi. Ankara, Türkiye; 2010.

15. Surek E, Buyukkileci AO. Production of xylooligosaccharides by autohydrolysis of hazelnut (Corylus avellana L.) shell. Carbohydr Polym. 2017;174: 565–571. doi:10.1016/j.carbpol.2017.06.109

16. Akcay C, Ceylan F, Arslan R. Production of oyster mushroom (Pleurotus ostreatus) from some waste lignocellulosic materials and FTIR characterization of structural changes. Sci Rep. 2023;13: 1–13. doi:10.1038/s41598-023-40200-x

17. Akcay C, Yalcin M. Morphological and chemical analysis of Hylotrupes bajulus (old house borer) larvae-damaged wood and its FTIR characterization. Cellulose. 2021;28:

1295–1310. doi:10.1007/s10570-020-03633-5

18. Wise LE, Jahn EC. Wood chemistry. New York: Reinhold Publishing Cooperation, Vol. 2; 1952.

19. Alpha-, beta- and gamma-cellulose in pulp. TAPPI T203 cm-09 TAPPI Press, TAPPI T203 cm-09; 2009.

20. Kacar B. Chemical analysis of plant and soil: III, Soil Analysis. Ankara: Ankara University Faculty of Agriculture, Education Res. Extension Found. Publications No. 3; 1994.

21. L. Singleton V, Orthofer R, Lamuela-Revantos RM. Analysis of total phenols and other oxidation substrates and antioxidants by means of folin-ciocalteu reagent. Methods Enzym. 1999;299: 152–178. doi:10.1016/S0076-6879(99)99017-1

22. Environmental solid matrices- Determination of elements using inductively coupled plasma optical emission spectrometry (ICP-OES). ISO 22036 ISO 22036; 2024.

23. Gurung OK, Budathoki U, Parajuli G. Effect of Different Substrates on the Production of Ganoderma lucidum (Curt.:Fr.) Karst. Our Nat. 2013;10: 191–198. doi:10.3126/on.v10i1.7781

24. Roy S, Jahan MAA, Das KK, Munshi SK, Noor R. Artificial Cultivation of Ganoderma lucidum (Reishi Medicinal Mushroom) Using Different Sawdusts as Substrates. Am J Biosci. 2015;3: 178–182. doi:10.11648/j.ajbio.20150305.13

25. Özçelik E, Pekşen A. Hazelnut husk as a substrate for the cultivation of shiitake mushroom (Lentinula edodes). Bioresour Technol. 2007;98: 2652–2658. doi:10.1016/j.biortech.2006.09.020

26. Carrasco-Cabrera CP, Bell TL, Kertesz MA. Caffeine metabolism during cultivation of oyster mushroom (Pleurotus ostreatus) with spent coffee grounds. Appl Microbiol

Biotechnol. 2019;103: 5831–5841. doi:10.1007/s00253-019-09883-z
27. Yakupoglu G. The effects of different substrates on yield and quality of Ganoderma lucidum mushroom. Onokuz Mayis University, Samsun, Turkey. 2007.
28. Atila F. Comparative study on the mycelial growth and yield of Ganoderma lucidum (Curt.: Fr.) Karst. On different lignocellulosic wastes. Acta Ecol Sin. 2020;40: 153–157. doi:10.1016/J.CHNAES.2018.11.007
29. Modi HA, Shah P, Shukla MD, Lahiri SK. Determination of Total Phenolic Content and Antioxidant Activity of Ganoderma lucidum Collected from Dang District of Gujarat, India. Nat Prod An Indian J. 2014;10: 75–83. Available: http://link.springer.com/10.1007/978-3-642-16483-5_3977
30. Demirci AŞ, Sözeri Atik D, Palabiyik İ, Gülcü M. Bioactive properties of commercial reishi mushroom products in powder form. J Tekirdag Agric Fac. 2021;18: 273–281. doi:10.33462/jotaf.754131
31. Puliga F, Leonardi P, Minutella F, Zambonelli A, Francioso O. Valorization of Hazelnut Shells as Growing Substrate for Edible and Medicinal Mushrooms. Horticulturae. 2022;8. doi:10.3390/horticulturae8030214
32. Zhang W, Chen X, Liu G, Jin G, Li Y, Li G, et al. Study on the chemical changes of Quercus acuttisima by Ganoderma lucidum cultivation after different years by FTIR analysis. Spectrochim Acta - Part A Mol Biomol Spectrosc. 2022;266. doi:10.1016/j.saa.2021.120443
33. Munyendo L, Njoroge D, Hitzmann B. The Potential of Spectroscopic Techniques in Coffee Analysis—A Review. Processes. 2022;10: 1–25. doi:10.3390/pr10010071
34. Craig AP, Botelho BG, Oliveira LS, Franca AS. Mid infrared spectroscopy and chemometrics as tools for the classification of roasted coffees by cup quality. Food Chem.

2018;245: 1052–1061. doi:10.1016/j.foodchem.2017.11.066
35. Pujol D, Liu C, Gominho J, Olivella MÀ, Fiol N, Villaescusa I, et al. The chemical composition of exhausted coffee waste. Ind Crops Prod. 2013;50: 423–429. doi:10.1016/j.indcrop.2013.07.056
36. Adapa PK, Schonenau LG, Canam T, Dumonceaux T. Quantitative Analysis of Lignocellulosic Components of Non-Treated and Steam Exploded Barley, Canola, Oat and Wheat Straw Using Fourier Transform Infrared Spectroscopy. J Agric Sci Technol. 2011;B 1: 177–188. Available: http://works.bepress.com/thomas_canam/2%5Cnhttp://works.bepress.com/cgi/viewcontent.cgi?article=1003&context=thomas_canam%5Cnhttp://works.bepress.com/thomas_canam/2/
37. Craig AP, Franca AS, Oliveira LS. Evaluation of the potential of FTIR and chemometrics for separation between defective and non-defective coffees. Food Chem. 2012;132: 1368–1374. doi:10.1016/j.foodchem.2011.11.121
38. Belchior V, Botelho BG, Casal S, Oliveira LS, Franca AS. FTIR and Chemometrics as Effective Tools in Predicting the Quality of Specialty Coffees. Food Anal Methods. 2020;13: 275–283. doi:10.1007/s12161-019-01619-z
39. Bari E, Nazarnezhad N, Kazemi SM, Tajick Ghanbary MA, Mohebby B, Schmidt O, et al. Comparison between degradation capabilities of the white rot fungi Pleurotus ostreatus and Trametes versicolor in beech wood. Int Biodeterior Biodegrad. 2015;104: 231–237. doi:10.1016/j.ibiod.2015.03.033
40. Lun LW, Gunny AAN, Kasim FH, Arbain D. Fourier transform infrared spectroscopy (FTIR) analysis of paddy straw pulp treated using deep eutectic solvent. AIP Conf Proc. 2017;1835. doi:10.1063/1.4981871

41. Reis N, Botelho BG, Franca AS, Oliveira LS. Simultaneous Detection of Multiple Adulterants in Ground Roasted Coffee by ATR-FTIR Spectroscopy and Data Fusion. Food Anal Methods. 2017;10: 2700–2709. doi:10.1007/s12161-017-0832-3

42. Wickramasinghe MA, Nadeeshani H, Sewwandi SM, Rathnayake I, Kananke TC, Liyanage R. Comparison of nutritional composition, bioactivities, and FTIR- ATR microstructural properties of commercially grown four mushroom species in Sri Lanka; Agaricus bisporus, Pleurotus ostreatus, Calocybe sp. (MK-white), Ganoderma lucidum. Food Prod Process Nutr. 2023;5. doi:10.1186/s43014-023-00158-9

43. Synytsya A, Bleha R, Skrynnikova A, Babayeva T, Čopíková J, Kvasnička F, et al. Mid-Infrared Spectroscopic Study of Cultivating Medicinal Fungi Ganoderma: Composition, Development, and Strain Variability of Basidiocarps. J Fungi. 2024;10. doi:10.3390/jof10010023

44. Zhu Y, Tan ATL. Discrimination of Wild-Grown and Cultivated *Ganoderma lucidum* by Fourier Transform Infrared Spectroscopy and Chemometric Methods. Am J Anal Chem. 2015;6: 480–491. doi:10.4236/ajac.2015.65047

45. Koutrotsios G, Patsou M, Mitsou EK, Bekiaris G, Kotsou M, Tarantilis PA, et al. Valorization of olive by-products as substrates for the cultivation of ganoderma lucidum and pleurotus ostreatus mushrooms with enhanced functional and prebiotic properties. Catalysts. 2019;9. doi:10.3390/catal9060537

46. Zhang R, Li X, Fadel JG. Oyster mushroom cultivation with rice and wheat straw. Bioresour Technol. 2002;82: 277–284. doi:10.1016/S0960-8524(01)00188-2

47. Akcay C, Arslan R, Ceylan F. Valorization of hazelnut branch pruning wastes to medicinal mushroom (Reishi-Ganoderma lucidum) cultivation and nutritional quality properties. Düzce Univ Fac For J For. 2024;20: 119–131. doi:10.58816/duzceod.1597211

Click here to access/download;Figure;Fig1.tif

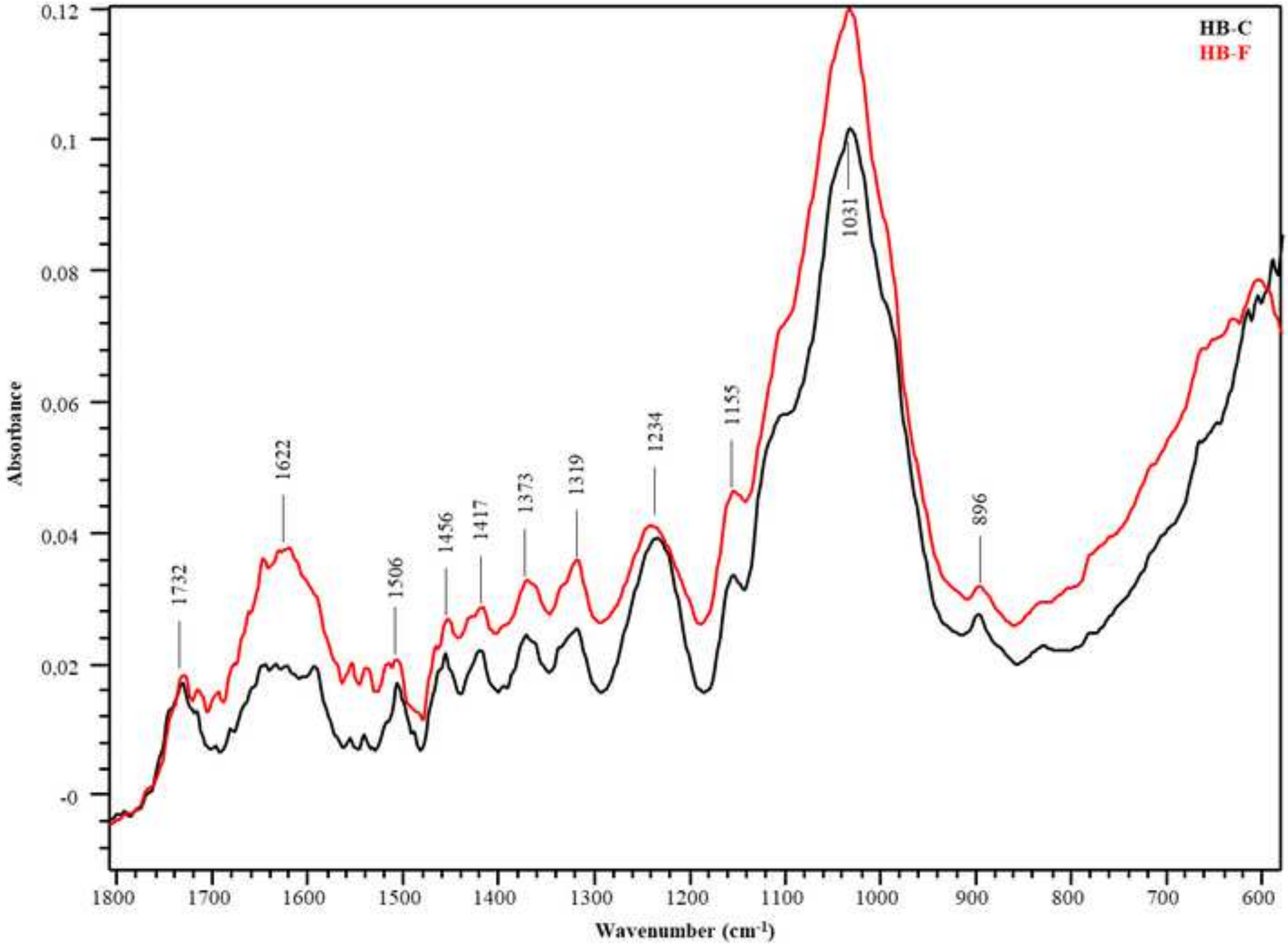

Fig2

Click here to access/download;Figure;Fig2.tif

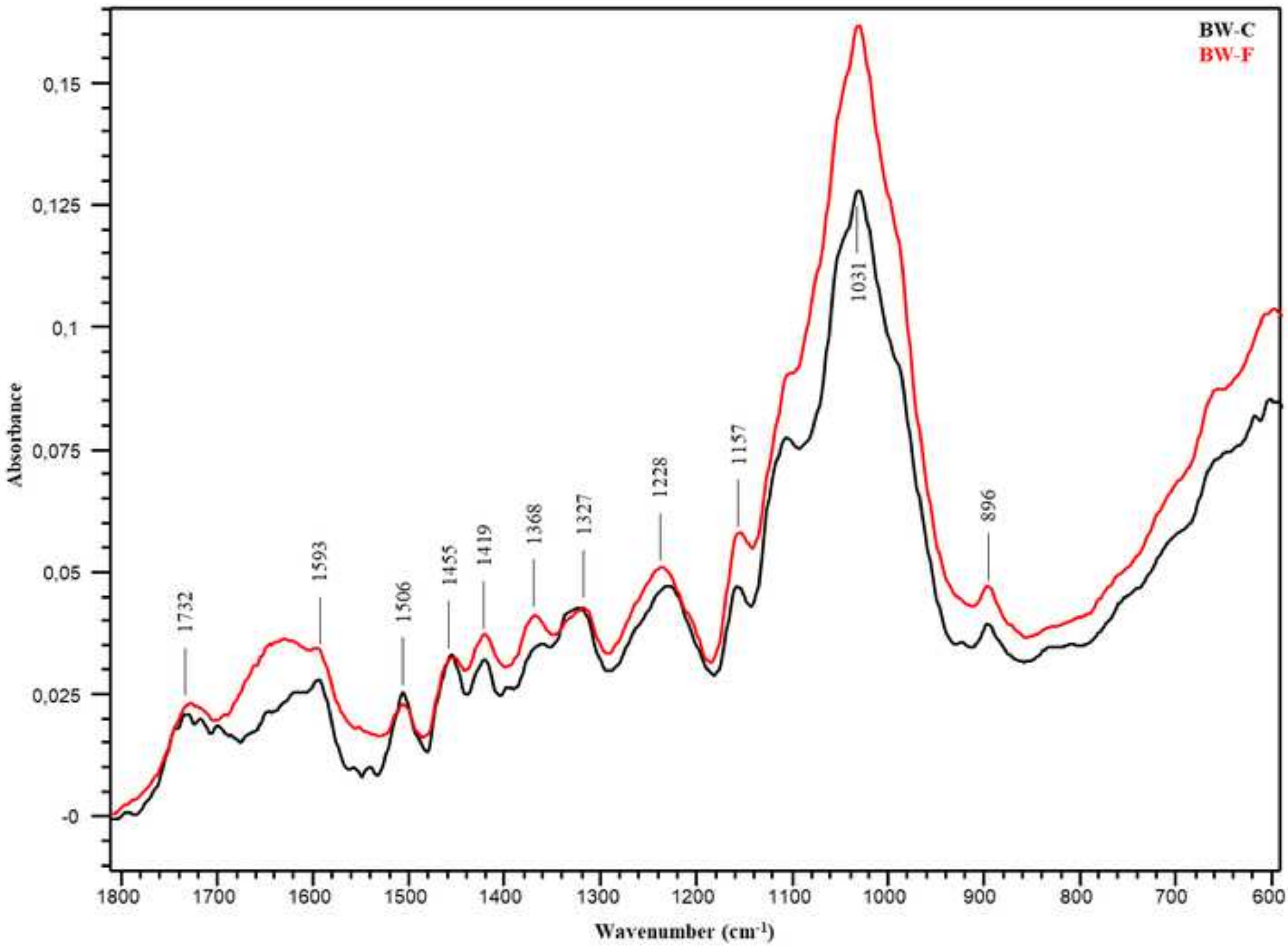

Click here to access/download;Figure;Fig3.tif

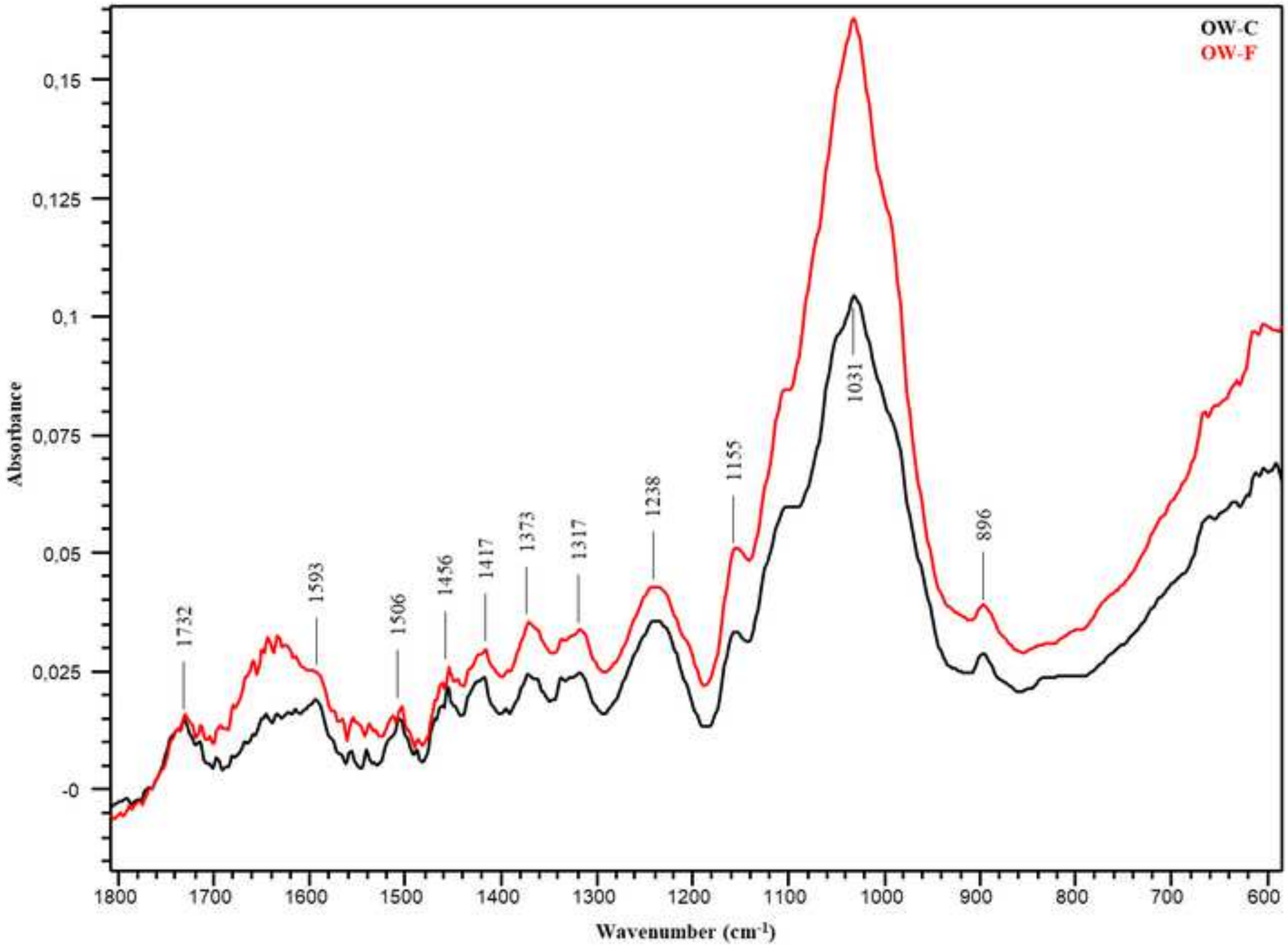

Fig4

Click here to access/download;Figure;Fig4.tif

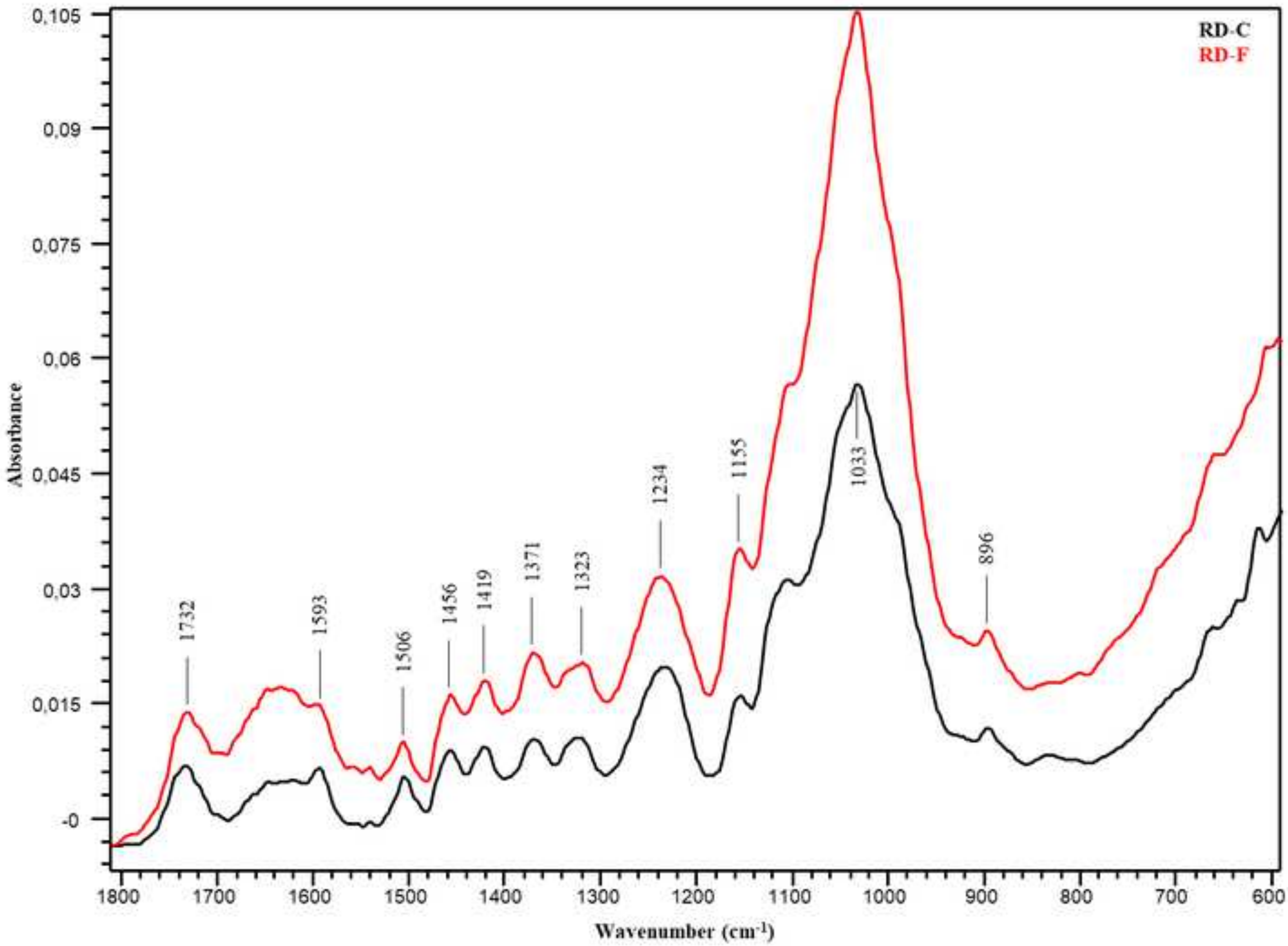

Click here to access/download;Figure;Fig5.tif

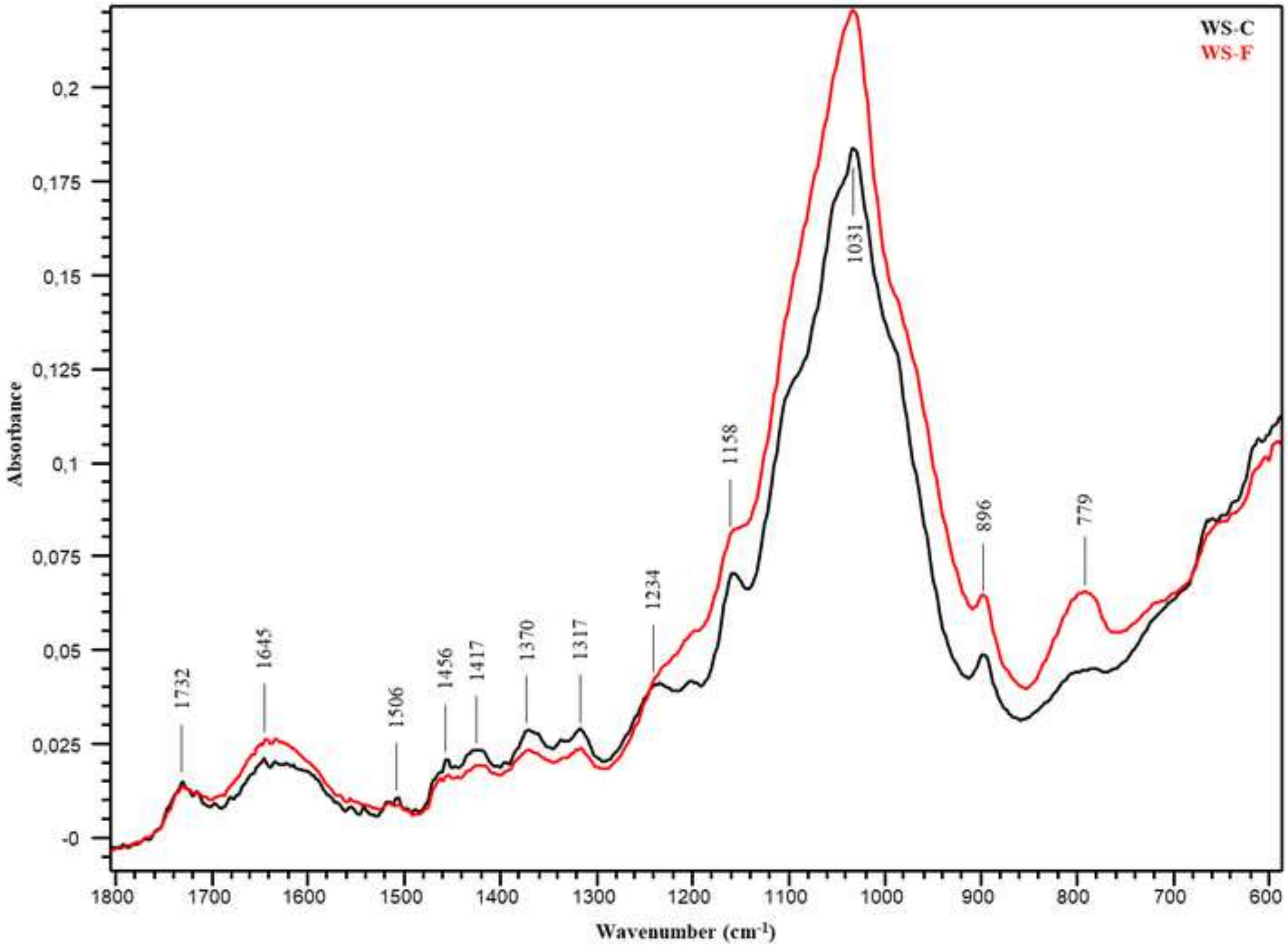

 Click here to access/download;Figure;Fig6.tif

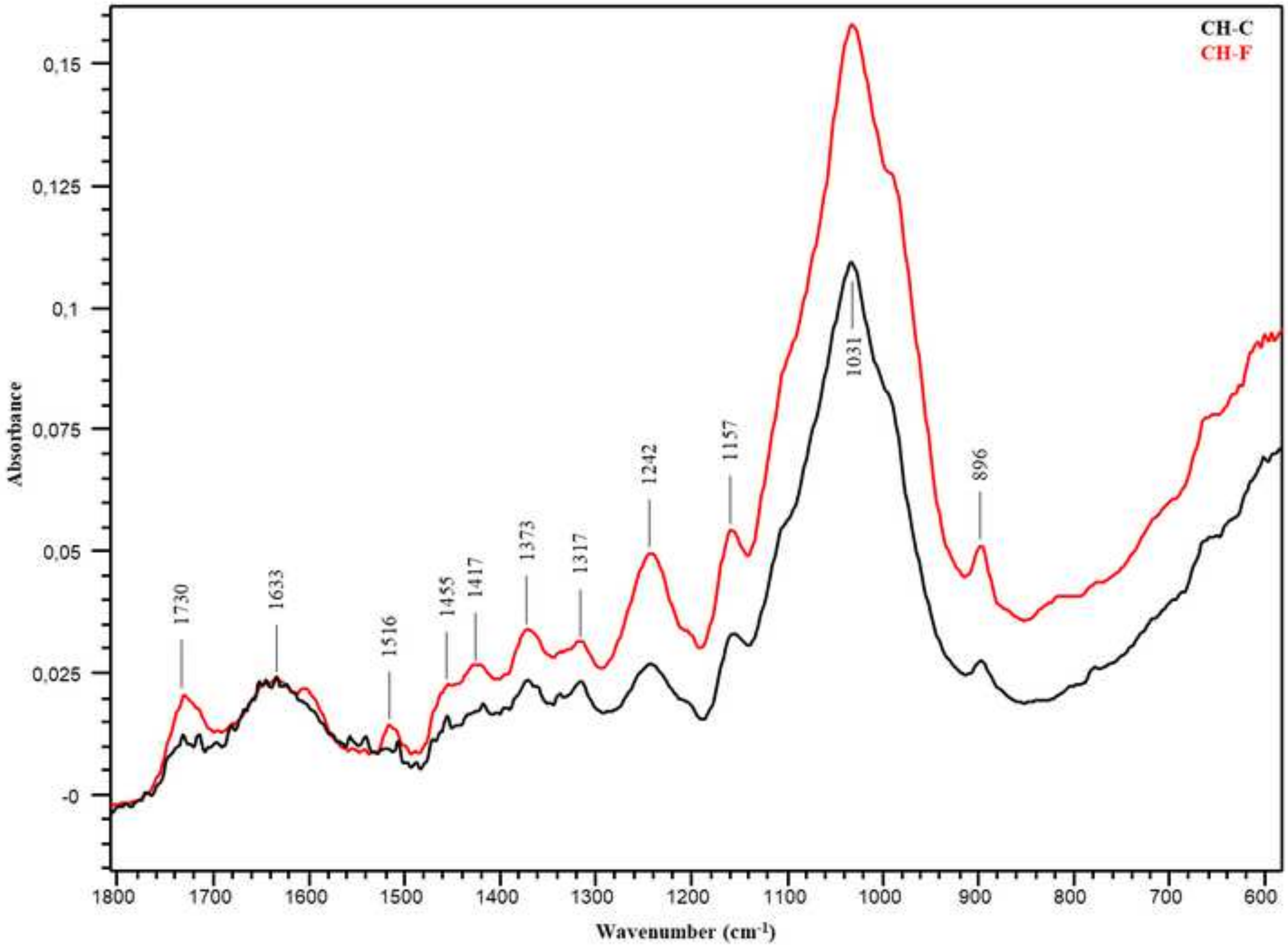

Fig7

Click here to access/download;Figure;Fig7.tif

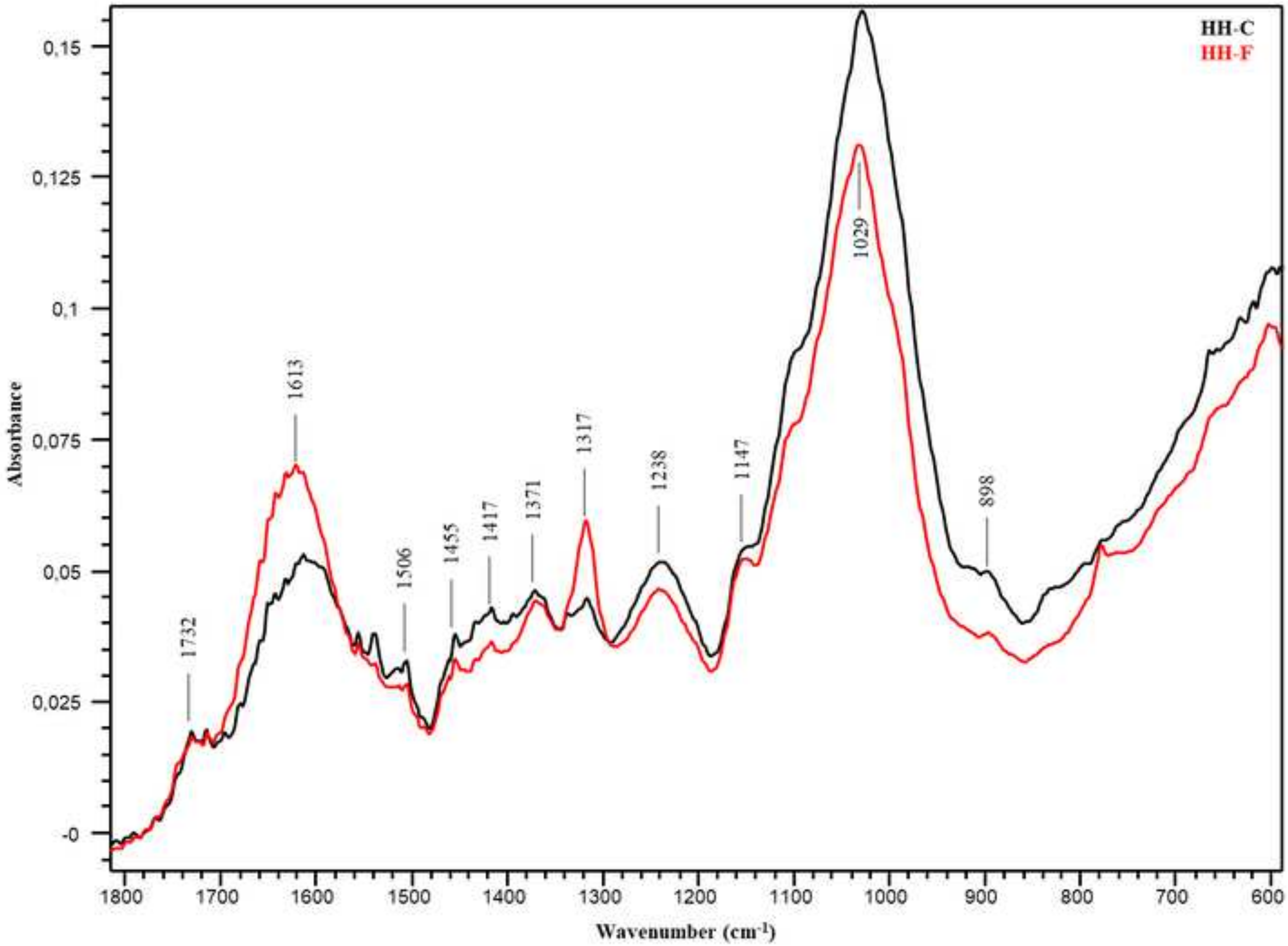

 Click here to access/download;Figure;Fig8.tif

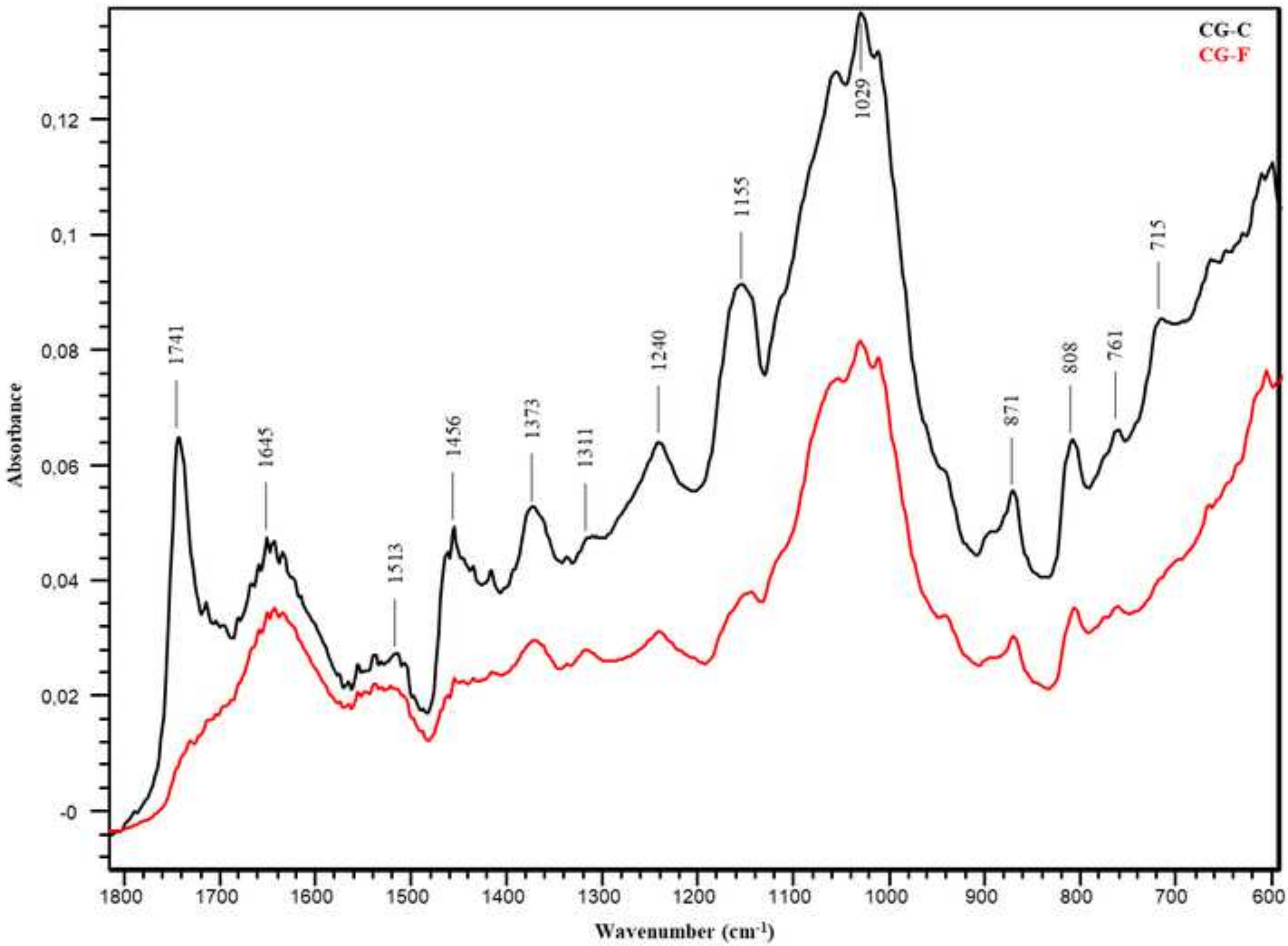

Fig9

Click here to access/download;Figure;Fig9.tif

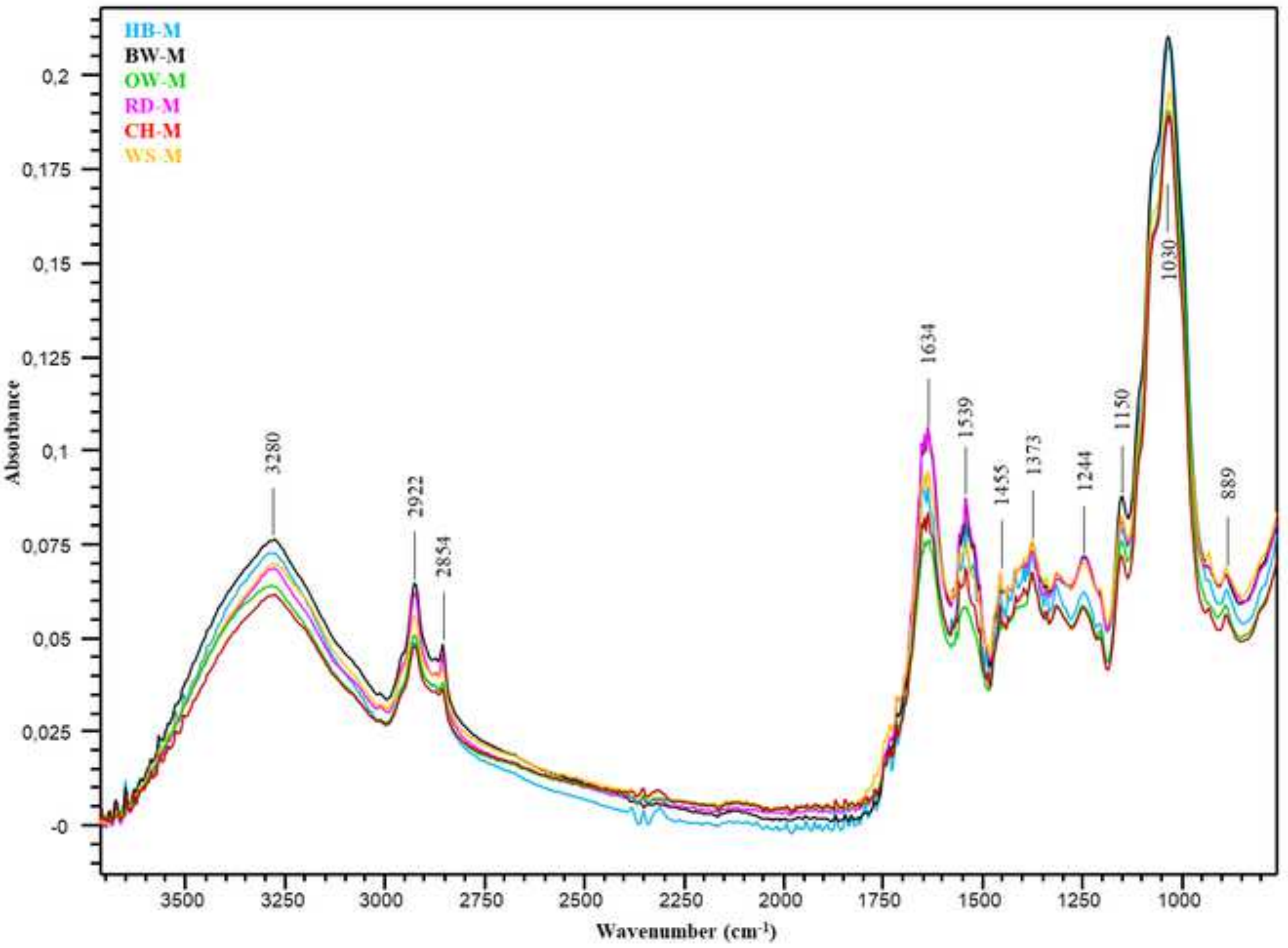

Supplement: Attachment — Submitted filename: PONE-D-25-12794_reviewer 19 March 2025.pdf [file pone.0328732.s003.pdf]
