# Supplementary material for: Valorization of various lignocellulosic wastes to Ganoderma lucidum (Curtis) P. Karst (Reishi Mushroom) cultivation and their FT-IR assessments
Source: PLoS One. 2025 Jul 22;20(7):e0328732. doi: 10.1371/journal.pone.0328732 (PMC12282901; doi:10.1371/journal.pone.0328732)
Supplement: Attachment — Submitted filename: PONE-D-25-14749_reviewer 18 April 2025 (1)N.pdf [file pone.0328732.s005.pdf]

PLOS ONE

Isolation and Characterization of Actinomycin D from Streptomyces kunmingensis BH111 Isolated from Beehives

--Manuscript Draft--

Manuscript Number:	PONE-D-25-14749
Article Type:	Research Article
Full Title:	Isolation and Characterization of Actinomycin D from Streptomyces kunmingensis BH111 Isolated from Beehives
Short Title:	Actinomycin D from Streptomyces kunmingensis BH111 Isolated from Beehives
Corresponding Author:	Thongchai Taechowisan, Ph.D.<br>Silpakorn University - Sanam Chandra Palace Campus<br>Nakhon Pathom, Nakhon pathom THAILAND
Keywords:	actinomycin D; antibacterial activity; anticancer activity; beehive materials; Streptomyces kunmingensis BH111
Abstract:	This study investigated the antibacterial potential of actinomycetes isolated from the beehive of Apis florea bees. Twenty isolates were obtained and screened for antibacterial activity against test bacteria (Bacillus cereus TISTR687, Bacillus subtilis TISTR008, Staphylococcus aureus TISTR885, Staphylococcus epidermidis TISTR518, methicillin-resistant Staphylococcus aureus Sp3) using the soft-agar overlay method. Isolate BH111 exhibited the strongest antibacterial effect. Morphological, chemotaxonomic, and 16S rDNA gene sequence analysis identified BH111 as Streptomyces kunmingensis with 99.40% similarity to known strains. Furthermore, a bioactive metabolite isolated from the culture medium of BH111 was characterized as actinomycin D, with a production yield of 39.86 mg g-1 of crude extract. This represents the first report of such a high yield of actinomycin D from this particular Streptomyces strain. The isolated actinomycin D exhibited broad-spectrum antibacterial activity against the tested bacterial strains. The minimum inhibitory concentration (MIC) and minimum bactericidal concentration (MBC) values ranged from 16–32 µg mL-1 and 128–256 µg mL-1, respectively. Additionally, the study explored the anticancer potential of the isolated actinomycin D, demonstrating effectiveness against human breast cancer (MDA-MB-231) and cervical cancer (HeLa) cells with IC50 values of 7.85 µg mL-1 and 3.20 µg mL-1, respectively. However, it also exhibited moderate cytotoxicity on non-cancerous Vero cells (IC50 = 8.46 µg mL-1). These findings highlight the potential of beehive-associated Streptomyces kunmingensis BH111 as a source of the valuable antibiotic actinomycin D, with promising applications against various bacteria and cancer cells.
Order of Authors:	Thongchai Taechowisan, Ph.D.
	Thanaporn Chuen-Im
	Benjamart Ruangrote
	Waya Sengpracha Phutdhawong
Additional Information:	
**Question**	**Response**
**Financial Disclosure**<br><br>Enter a financial disclosure statement that describes the sources of funding for the work included in this submission. Review the submission guidelines for detailed requirements. View published research articles from *PLOS ONE* for specific examples.	Yes

Powered by Editorial Manager® and ProduXion Manager® from Aries Systems Corporation

This statement is required for submission and **will appear in the published article** if the submission is accepted. Please make sure it is accurate.<br><br>**Funded studies**<br>Enter a statement with the following details:<br>• Initials of the authors who received each award<br>• Grant numbers awarded to each author<br>• The full name of each funder<br>• URL of each funder website<br>• Did the sponsors or funders play any role in the study design, data collection and analysis, decision to publish, or preparation of the manuscript?<br><br>Did you receive funding for this work?	
Please add funding details.<br>as follow-up to "**Financial Disclosure**<br><br>Enter a financial disclosure statement that describes the sources of funding for the work included in this submission. Review the submission guidelines for detailed requirements. View published research articles from *PLOS ONE* for specific examples.<br><br>This statement is required for submission and **will appear in the published article** if the submission is accepted. Please make sure it is accurate.<br><br>**Funded studies**<br>Enter a statement with the following details:<br>• Initials of the authors who received each award<br>• Grant numbers awarded to each author<br>• The full name of each funder<br>• URL of each funder website<br>• Did the sponsors or funders play any role in the study design, data collection and analysis, decision to publish, or preparation of the manuscript?	Seed Grant SRIF-JRG-2568-08 from the Faculty of Science, Silpakorn University, Nakhon Pathom, Thailand, provided funding for this project.

Powered by Editorial Manager® and ProduXion Manager® from Aries Systems Corporation

Did you receive funding for this work?"

Please select the country of your main research funder (please select carefully as in some cases this is used in fee calculation).<br>as follow-up to "**Financial Disclosure**<br><br>Enter a financial disclosure statement that describes the sources of funding for the work included in this submission. Review the submission guidelines for detailed requirements. View published research articles from *PLOS ONE* for specific examples.<br><br>This statement is required for submission and **will appear in the published article** if the submission is accepted. Please make sure it is accurate.<br><br>**Funded studies**<br>Enter a statement with the following details:<br>• Initials of the authors who received each award<br>• Grant numbers awarded to each author<br>• The full name of each funder<br>• URL of each funder website<br>• Did the sponsors or funders play any role in the study design, data collection and analysis, decision to publish, or preparation of the manuscript?<br><br>Did you receive funding for this work?"	THAILAND - TH
**Competing Interests**<br><br>Use the instructions below to enter a competing interest statement for this submission. On behalf of all authors, disclose any competing interests that could be perceived to bias this work—acknowledging all financial support and any other relevant financial or non-financial competing interests.<br><br>This statement is required for submission	The authors have declared that no competing interest exists.

and **will appear in the published article** if the submission is accepted. Please make sure it is accurate and that any funding sources listed in your Funding Information later in the submission form are also declared in your Financial Disclosure statement.<br><br>View published research articles from *PLOS ONE* for specific examples.<br><br>**NO authors have competing interests**<br><br>Enter: *The authors have declared that no competing interests exist.*<br><br>**Authors with competing interests**<br><br>Enter competing interest details beginning with this statement:<br><br>*I have read the journal's policy and the authors of this manuscript have the following competing interests: [insert competing interests here]*<br><br>* typeset	
**Ethics Statement**<br><br>Enter an ethics statement for this submission. This statement is required if the study involved:<br><br>• Human participants<br>• Human specimens or tissue<br>• Vertebrate animals or cephalopods<br>• Vertebrate embryos or tissues<br>• Field research<br><br>Write "N/A" if the submission does not require an ethics statement.<br><br>General guidance is provided below. Consult the submission guidelines for detailed instructions. **Make sure that all information entered here is included in the Methods section of the manuscript.**	The field permit number is SRIF-JRG-2568-08 from the Faculty of Science, Silpakorn University, Nakhon Pathom, Thailand.

Powered by Editorial Manager® and ProduXion Manager® from Aries Systems Corporation

**Format for specific study types**<br><br>**Human Subject Research (involving human participants and/or tissue)**<br>• Give the name of the institutional review board or ethics committee that approved the study<br>• Include the approval number and/or a statement indicating approval of this research<br>• Indicate the form of consent obtained (written/oral) or the reason that consent was not obtained (e.g. the data were analyzed anonymously)<br><br>**Animal Research (involving vertebrate animals, embryos or tissues)**<br>• Provide the name of the Institutional Animal Care and Use Committee (IACUC) or other relevant ethics board that reviewed the study protocol, and indicate whether they approved this research or granted a formal waiver of ethical approval<br>• Include an approval number if one was obtained<br>• If the study involved *non-human primates*, add *additional details* about animal welfare and steps taken to ameliorate suffering<br>• If anesthesia, euthanasia, or any kind of animal sacrifice is part of the study, include briefly which substances and/or methods were applied<br><br>**Field Research**<br><br>Include the following details if this study involves the collection of plant, animal, or other materials from a natural setting:<br>• Field permit number<br>• Name of the institution or relevant body that granted permission	
**Data Availability**<br><br>Authors are required to make all data underlying the findings described fully available, without restriction, and from the time of publication. PLOS allows rare exceptions to address legal and ethical concerns. See the PLOS Data Policy and FAQ for detailed information.	Yes - all data are fully available without restriction

A Data Availability Statement describing where the data can be found is required at submission. Your answers to this question constitute the Data Availability Statement and **will be published in the article**, if accepted.<br><br>**Important:** Stating 'data available on request from the author' is not sufficient. If your data are only available upon request, select 'No' for the first question and explain your exceptional situation in the text box.<br><br>Do the authors confirm that all data underlying the findings described in their manuscript are fully available without restriction?	
**Describe where the data may be found in full sentences. If you are copying our sample text, replace any instances of XXX with the appropriate details.**<br><br>• If the data are **held or will be held in a public repository**, include URLs, accession numbers or DOIs. If this information will only be available after acceptance, indicate this by ticking the box below. For example: *All XXX files are available from the XXX database (accession number(s) XXX, XXX.*).<br>• If the data are all contained **within the manuscript and/or Supporting Information files**, enter the following: *All relevant data are within the manuscript and its Supporting Information files.*<br>• If neither of these applies but you are able to provide **details of access elsewhere**, with or without limitations, please do so. For example:<br><br>*Data cannot be shared publicly because of [XXX]. Data are available from the XXX Institutional Data Access / Ethics Committee (contact via XXX) for researchers who meet the criteria for access to confidential data.*<br><br>*The data underlying the results presented in the study are available from (include the name of the third party*	Data are available in GenBank under the accession number PP753780 and PQ231004.

and contact information or URL).<br>• This text is appropriate if the data are owned by a third party and authors do not have permission to share the data.<br><br>* typeset	
Additional data availability information:	Tick here if the URLs/accession numbers/DOIs will be available only after acceptance of the manuscript for publication so that we can ensure their inclusion before publication.

Powered by Editorial Manager® and ProduXion Manager® from Aries Systems Corporation

Manuscript Click here to access/download;Manuscript;PLOS ONE-Act_D_beehive.docx

~~Isolation and~~ Characterization of Actinomycin D from *Streptomyces kunmingensis* BH111 Isolated from Beehives

Formatted: Font color: Red, Strikethrough

Thongchai Taechowisan[1*], Thanaporn Chuen-Im[1], Benjamart Ruangrote[2], Waya S. Phutdhawong[2]

[1] Department of Microbiology, Faculty of Science, Silpakorn University, Nakhon Pathom 73000, Thailand.

[2] Department of Chemistry, Faculty of Science, Silpakorn University, Nakhon Pathom 73000, Thailand.

*Corresponding Author

E-mail: tewson84@hotmail.com (TT)

Author Contributions

Designed the experiments: TT. Performed the experiments: TT TC and BR. Analyzed the data: WSP. Wrote the paper: TT BR.

Abstract

This study investigated the antibacterial potential of actinomycetes isolated from the beehive of *Apis florea* bees. Twenty isolates were obtained and screened for antibacterial activity against the test bacteria (*Bacillus cereus* TISTR687, *Bacillus subtilis* TISTR008, *Staphylococcus aureus* TISTR885, *Staphylococcus epidermidis* TISTR518, methicillin-resistant *Staphylococcus aureus* Sp3) using the soft-agar overlay method. Isolate BH111 exhibited the strongest antibacterial effect. Morphological, chemotaxonomic, and 16S rDNA gene sequence analysis identified BH111 as *Streptomyces kunmingensis* with 99.40% similarity to known strains. Furthermore, a bioactive metabolite isolated from the culture medium of BH111 was characterized as actinomycin D, with a production yield of 39.86 mg g^{-1} of crude extract. This represents the first report of such a high yield of actinomycin D from this particular *Streptomyces* strain. The isolated actinomycin D exhibited broad-spectrum antibacterial activity against the tested bacterial strains. The minimum inhibitory concentration (MIC) and minimum bactericidal concentration (MBC) values ranged from 16–32 µg mL^{-1} and 128–256 µg mL^{-1}, respectively. Additionally, the study explored the anticancer potential of the isolated actinomycin D, demonstrating effectiveness against human breast cancer (MDA-MB-231) and cervical cancer (HeLa) cells with IC_{50} values of 7.85 µg mL^{-1} and 3.20 µg mL^{-1}, respectively. However, it also exhibited moderate cytotoxicity on non-cancerous Vero cells (IC_{50} = 8.46 µg mL^{-1}). These findings highlight the potential of beehive-associated *Streptomyces kunmingensis* BH111 as a source of the valuable antibiotic actinomycin D, with promising applications against various bacteria and cancer cells.

Abstract

Lacks brief methods and keywords. Quantitative data of the major findings not indicated. Finally, statistical analysis for the obtained findings not given.

Formatted: Font color: Red, Strikethrough

Formatted: Highlight

Formatted: Font: 11 pt

Formatted: List Paragraph, Line spacing: single, No bullets or numbering, Tab stops: Not at 1.07 cm

Formatted: Font color: Red

Formatted: List Paragraph, Line spacing: single, No bullets or numbering, Tab stops: Not at 1.07 cm

Formatted: Font color: Red

44

45

Introduction

Actinomycetales, nature's microbial masters of adaptation, conquer diverse habitats from common soil to insect guts [Needs rewriting]. In soil, they break down organic matter, fueling the ecosystem. They even form partnerships with plants, acting as nitrogen fixers in exchange for plant-produced sugars. ~~Unafraid o~~f Actinomycetes are common in salty environments or freshwater frontiers, ~~actinomycetes~~ and thrive in seabeds, salt marshes, lakes, and rivers. They even venture into the internal world, residing in animal guts and aiding digestion for their insect hosts [Re-write!]. This remarkable habitat diversity highlights the ecological significance of actinomycetes, showcasing their adaptability and their ~~essential role~~ importance in the health of various ecosystems [1]. Actinomycetes have been found in beehives, including in worker bee tissue, brood cells, and hive materials [2–5]. The presence of actinomycetes in beehives could benefit the bees in several ways, including their ability to produce bioactive molecules. Actinomycetes are known to produce a variety of antimicrobial compounds, including antibiotics. These compounds could help to protect the bees from bacterial pathogens [4, 6]. Actinomycetes are known to produce a wide range of bioactive compounds, including antibiotics, enzymes, and antifungals. These compounds are believed to play a role in helping actinomycetes compete with other microbes and survive in their environment [7]. Given the presence of these diverse compounds in other actinomycetes, it's possible that similar compounds are produced by those found in beehives. However, research specifically isolating and identifying compounds from beehive actinomycetes is still developing. Recently, *Streptomyces* sp. AmelAP-1 was isolated from pollen stores of beehives which could stop the growth of *Paenibacillus larvae*. A specific compound produced by this *Streptomyces* strain was elucidated as piceamycin. This piceamycin

Formatted: Highlight

Formatted: Font color: Red

Formatted: Font color: Red, Strikethrough

Formatted: Font color: Red

Formatted: Font color: Red, Strikethrough

Formatted: Font color: Red

Formatted: Highlight

Formatted: Font color: Red

Formatted: Font color: Red, Strikethrough

Formatted: Font color: Red

appears to be the secret weapon against the entomopathogenic bacteria [4].

In this study, Actinomycetes were isolated from beehives of *Apis florea*, and their antibacterial activity was investigated. ~~The BH111 strain exhibited the most potent antibacterial activity. It was identified based on morphological, physiological, and chemotaxonomic characterizations. Additionally, the chemical structure of the major compound isolated from the BH111 strain was elucidated. We also investigated the antibacterial properties of the major compound against reference strains and a clinical isolate of MRSA, and evaluated its cytotoxicity using the MTT assay on African green monkey kidney cells (Vero). The compound's effects were assessed on human breast carcinoma cells (MDA-MB-231) and human cervical carcinoma cells (HeLa), using the MTT assay to evaluate cytotoxicity~~. It better to show clearly the knowledge gap/s/ rather than presenting redundant ideas that otherwise can be addressed in the results section.

Formatted: Font color: Red, Strikethrough

Formatted: Font color: Red, Highlight

Formatted: Not Strikethrough, Highlight

Formatted: Font color: Red, Highlight

Materials & Methods

Sample collection and actinomycetes isolation

Two beehives of *Apis florea* were collected from the Circle House Kanchanaburi (14.02762 N, 99.51710 E), Thailand during June – July 2023 by beekeepers. This project received approval for a field experiment from the Faculty of Science, Silpakorn University, with approval number SRIF-JRG-2567-04. The collected samples were immediately tightly sealed in to plastic bags to prevent contamination and transported to our facilities at Silpakorn University, where 10 grams of the samples were added 90 mL of phosphate buffer saline (PBS) in stomacher bag, the samples were then mechanically homogenized for 10 min. One hundred microliters of samples were spread onto humic-acid vitamin agar containing cycloheximide (50 µg mL^{-1}) and nalidixic acid (20 µg mL^{-1}), and incubated at 32°C for 2 weeks. Purified colonies were visually identified as actinomycetes based on their morphology and transferred to International *Streptomyces* Project

medium 2 (ISP-2) [8].

Queries: 1) What selection criteria were used to choose the study site? 2) How the sample size was determined? 3) Is this sample size statistically acceptable? 4) What was the basis for choosing 10 g to 90? Why not 9 g to 90? The former is uncommon approach in Food Microbiology!

Formatted: Font color: Red

Formatted: Font color: Red

Screening of antibacterial actinomycetes and extraction of bioactive compounds

In total, 20 actinomycete isolates were tested for their antimicrobial properties against *Bacillus cereus* TISTR687, *Bacillus subtilis* TISTR008, *Staphylococcus aureus* TISTR885, *Staphylococcus epidermidis* TISTR518, and the clinical isolate of methicillin-resistant *Staphylococcus aureus* (MRSA) Sp3 described by Taechowisan *et al*. [9]. The Antimicrobial activity of actinomycete strains was carried out using soft-agar overlay method [10]. The inhibition zones were observed and measured. The experiment was conducted in triplicate. Among the 20 actinomycete isolates, the BH111 exhibited the most potent antibacterial property. It was identified based on morphological, physiological, and chemotaxonomic characterizations in accordance with the methods developed by Taechowisan and Lumyong [11]. The strain BH111 was cultured on ISP-2 agar (500 Petri dishes) for 21 days at 32°C. The metabolites were extracted using the methods described by Taechowisan *et al*. [12]. The culture medium was cut into small pieces[References needed!] that were extracted with ethyl acetate (3 x 5 L). This organic solvent was pooled and then taken to dryness under rotary evaporation to obtain a dark brown solid (9.50 g). Thin-layer chromatography-direct bioautography (TLC-DB) was carried out according to the methods described by Jesionek *et al*. [13]. The crude extracts were divided to two parts, part 1 was resuspended in 5 mL of sterile dimethyl sulfoxide (DMSO) to obtain an initial concentration of 10.24 mg mL^{-1}, and stored at -20°C until testing for antibacterial and anticancer activities. Part 2 was re-suspended in dichloromethane (CH_2Cl_2) to carry out compound purification and characterisation.

Queries: How the isolates inoculated? What was the inoculum size? How inoculum size determined? What incubation conditions maintained? For how long the inoculated plates were incubated? What standard antibiotic was used as positive control? Why only 20 isolates? Why the author/s/ failed to go for a large number of isolates; may be 100 or more?

Formatted: Highlight

Formatted: Highlight

Formatted: Font color: Red

Formatted: Font: 11 pt

Formatted: List Paragraph, Line spacing: single, No bullets or numbering, Tab stops: Not at 1.28 cm

Formatted: Font color: Red

Formatted: Font color: Red

Formatted: Font color: Red

Identification of the selected strain

The strain BH111 was selected for identification. Morphological characteristics, presence of aerial mycelium, spore mass colour, distinctive reverse colony colour, diffusible pigment,

sporophore and spore chain morphology were recorded after 15 days incubation on ISP-2 medium. Diaminopimelic acid isomers and sugars from whole-cell extract were analysed for chemotaxonomic studies [14, 15].

Scanning electron microscopy of the selected strain

Scanning electron microscopy (SEM) was used to observe the morphology of the selected strain. Sample was prepared according to the methods described by Castillo *et al*. [16]. The resulting preparations were dried at the critical point of liquid CO_2 (Quorum K850, United Kingdom), sputter coated with gold (Safematic CCU-010HV, Switzerland), and examined with a scanning electron microscope (TESCAN Mira3, Czech Republic). Spore chain morphology and spore surface ornamentation of the selected isolate were recorded.

16S rDNA, *gyrB* gene sequencing and phylogenetic analysis of the selected strain

The selected isolate that showed the highest potential antibacterial activity against the test bacteria was grown in 50 mL of ISP-2 broth and incubated in a shaker at 150 rpm, 32°C for 7 days. The cells were collected via centrifugation (6,000 rpm) for 20 min, and were washed three times using PBS. Genomic DNA was extracted, and the 16S rDNA and *gyrB* genes were amplified using the methods described by Taechowisan *et al*. [17], and Yamamoto and Harayama [18], respectively. The primers used for 16S rDNA gene amplification were A7-26f (5' - CCGTCGACGAGCTCAGAGTTTGATCCTGGCTCAG-3')andB1523-1504r(5'-CCCGGGTACCAAGCTTAAGGAGGTGATCCAGCCGCA-3'), while the following primers were used for *gyrB* gene amplification: UP1f (5' – GAAGTCATCATGACCGTTCTGCAYGCNGGNGGNAARTTYGA-3') and UP2r (5' – AGCAGGGTACGGATGTGCGAGCCRTCNACRTCNGCRTCNGTCAT-3'. The PCR products were purified using a QIAquick gel extraction kit (Qiagen, Germany) according to the

manufacturer's instructions. Purified PCR products were sequenced by the Sanger method at 1st BASE, Singapore. The NCBI BLAST tool (https://blast.ncbi.nlm.nih.gov/Blast.cgi) was employed to confirm the sequence similarity of the actinomycete isolates to a reference species. The sequences of closely related type strains retrieved from the GenBank database were multiple aligned using Clustal W, then a phylogenetic tree was constructed using neighbor joining method in MEGA 11 software [19]. The 16S rDNA and *gyrB* gene sequences of *Microbispora corallina* JCM 10267 and *Microbispora corallina* JCM 10266 were used as outgroup, respectively.

Queries: How the qualities of genomic DNA and PCR products determined?

Determination of the minimum inhibitory concentration (MIC) and minimum bactericidal concentrations (MBC)

The antibacterial activity of the crude extract and the purified compound was assessed by determining their minimum inhibitory concentration (MIC) and minimum bactericidal concentration (MBC) against relevant bacterial strains. Established protocols outlined by Pfaller *et al*. [20] were employed to ensure consistent and reliable testing procedures. The commercial actinomycin D and chloramphenicol (Thermo Fisher Scientific, Massachusetts, USA) were utilized as positive controls, and 5% methanol was used as the solvent control. All experiments were performed in triplicate.

Determination of the cytotoxicity activity of the crude extract and purified compound

The *in vitro* cytotoxicity of the crude extract and the purified compound was evaluated using the MTT assay [21]. Both the crude extract and the purified compound were tested against two cancer cell lines [human cervical carcinoma (HeLa), and human breast carcinoma cells (MDA-MB-231)] at concentrations ranging from 1 to 512 µg mL^{-1}. To assess the selectivity of the compound for cancer cells, a non-cancerous cell line (Vero) was included. Selectivity indices (SI)

Formatted: Font: 10.5 pt

Formatted: List Paragraph, Line spacing: single, No bullets or numbering, Tab stops: Not at 1.28 cm

Formatted: Font color: Red

were calculated as the ratio of the IC_{50} (concentration required for 50% cell growth inhibition) in

the Vero cell line to the IC_{50} in the cancer cell lines. Higher SI values indicate greater selectivity of the compound for targeting cancer cells with minimal cytotoxicity towards healthy cells. Positive controls for cytotoxicity were established using commercially available actinomycin D and doxorubicin hydrochloride (Thermo Fisher Scientific, Massachusetts, USA).

Compound purification and characterisation

The crude extract (9.21 g) was fractionated on a column of ODS (700 g) eluting with CH_2Cl_2/MeOH (17:3, 18:2, and 19:1) MeOH to give four fractions of 3M, 2M, and 1M. Fraction 1M was separated by HPLC using a Zorbax SB-C18 column (250 x 9.4 mm, 5 µm; mobile phase 95% MeOH; flow rate 0.8 mL/min; detection wavelength: 440 nm) to give compound 1 (367.1 mg, t_R 25 min), and partially purified fraction 1M (11.3 mg, t_R 20 min). The compound 1 (red amorphous powder) was identified to be actinomycin D. This compound had a melting point of 258-260°C, electrospray ionization mass spectrometry (ESI-HRMS) m/z (rel. int.) : $(M + Na)^+$ (100%), calcd. for $C_{62}H_{86}N_{12}O_{16}Na$, 1277.6182; found, 1277.6315 , $(M + H)^+$ (55%) calcd. for $C_{62}H_{87}N_{12}O_{16}$, 1255.6363; found, 1255.6508 (S1 Fig). Its ^1H- and ^{13}C-NMR spectral data (S2 and S3 Figs) were identical with those of actinomycin D previously reported [22, 23, 24, 25, 26] (S4 Fig and S1 Table). The partially purified fraction 1M and other fractions will be reported elsewhere.

Formatted: Highlight

Statistical analyses

Data were expressed as means ± standard deviations (SD). Statistical analyses were performed using SPSS for Windows version 11.01 (SPSS Inc., Chicago, IL, USA). Treatment effects were analyzed using one-way ANOVA, followed by Tukey multiple comparisons. A p value < 0.05 was used to indicate significance. What version of SPSS? What about the means separation?

Formatted: Font color: Red

Results

A total of 20 ~~strains~~ isolates were collected from 2 beehives of *Apis florea* (S5 Fig) and further purified using the ISP-2 growth media (Figure/Table---?). Based on screening for antimicrobial activity using the soft-agar overlay method, one actinomycete isolate (BH111) showed potent activity (5.0 to 7.4 cm diameter inhibition zones) against the tested bacteria (S6 Fig).

Strain BH111 was cultured on ISP-2 growth media and presented cream-coloured colonies, white aerial mycelium, and orange pigment production. BH111 strain's cell wall was composed of the meso-isomer of 2,6-diaminopimelic acid, and no diagnostic sugars were detected in whole-cell hydrolysates, with only glucose and ribose being detected (Figure/Table---?). Scanning electron microscopic examination showed the strain possessed typical *Streptomyces* morphology (Figure/Table---?). The substrate mycelia were well developed, branched, did not fragment into bacillary elements or coccoid forms, and characterized by the flexuous hyphae with smooth surfaces (Fig 1 and S7 Fig). The 16S rDNA (1485 nt) sequence analysis using BLAST showed that the BH111 strain is closely related to *S. kunmingensis* strain DSD1764 and strain DSD250 with 99.40% identity. Phylogenetic tree of the 16S rDNA sequence shared a cluster with *Streptomyces kunmingensis* (Fig 2a). A phylogenetic tree based on *gyrB* gene sequences was also reconstructed (Fig 2b). The *gyrB* sequence similarity between the BH111 strain and *Streptomyces kunmingensis* NRRL B-16240 was 99.52%. This high similarity, along with the similar 16S rDNA sequence, strongly suggests that BH111 is closely ~~related to~~related to *Streptomyces kunmingensis*. Therefore, we designated BH111 as *Streptomyces kunmingensis* BH111. The 16S rDNA and *gyrB* gene sequences are accessible in GenBank under the accession number PP753780 and PQ231004, respectively. TLC-DB of a fraction of the extract

Formatted: Font color: Red, Strikethrough

Formatted: Font color: Red

Formatted: Font color: Red

Formatted: Font color: Red

eluted with CH_2CL_2/MeOH (9:1) tested against the bacterial strains revealed that there were at least two compounds with antibacterial activity. After isolating the compounds in the crude

extract, we obtained compound 1 as the major component, along with a few small quantities of partially purified fractions. There was a sufficient amount of compound 1 for further studies. The ^{1}H-NMR, ^{13}C-NMR and mass spectral data of compound 1 were identical with those of actinomycin D (S1—S4 Figs and S1 Table). It was found that the isolate BH111 could produce actinomycin D at 39.86 mg g^{-1} of crude extract. The further study about the partially purified fractions will be reported elsewhere.

Table 1 summarizes the antibacterial activity of the crude extract and purified compound, as determined by MIC and MBC values. The purified compound exhibited promising antibacterial activity. It inhibited the growth of *S. epidermidis* and *S. aureus*, including the clinical isolate MRSA Sp3. The MIC ranged from 16 to 32 µg mL^{-1}, while the MBC ranged from 128 to 256 µg mL^{-1}.

Table 1. MBC and MIC values of crude extract and purified actinomycin D against the test bacterial strains. How inhibition was reported? What selection criteria were used to target the tested strains? Why fungal pathogens excluded?

Test substances	MIC (µg mL^{-1})					MBC (µg mL^{-1})				
	**B.c.[a]**	**B.s.**	**S.a.**	**S.e.**	**MRSA Sp3**	**B.c.[a]**	**B.s.**	**S.a.**	**S.e.**	**MRSA Sp3**
**Crude extract**	16	64	32	16	64	128	256	256	128	256
**Purified actinomycin D**	16	32	32	16	32	256	256	128	128	256
**Chloramphenicol**	2	2	1	2	8	4	4	2	4	16

[a]B.c.; *Bacillus cereus* TISTR687, B.s.; *Bacillus subtilis* TISTR008, S.a.; *Staphylococcus aureus* TISTR885, S.e.; *Staphylococcus epidermidis* TISTR518, MRSA; methicillin-resistant *Staphylococcus aureus* Sp3 (the clinical isolates).

It exhibited a moderate antibacterial activity against *B. subtilis* and *B. cereus*. The crude

Formatted: Font color: Red

Formatted: Font color: Red

Formatted: Font color: Red

extract and purified compound were assessed for cytotoxicity using Vero and two cancer cells

(HeLa and MDA-MB-231). They exhibited high cytotoxicity, with IC_{50} values ranged from 3.20 — 12.30 µg mL^{-1} against two cancer cell lines (HeLa and MDA-MB-231; Figure/Table---?). The crude extract and purified compound showed moderate cytotoxicity against non-cancerous cell line (Vero), with IC_{50} values of 12.52 and 8.46 µg mL^{-1}, respectively. However, the selectivity indices (SI) of crude extract and purified compound against the cancer cell lines were significantly (p=?) lower compared to doxorubicin hydrochloride (Table 2).

Formatted: Font color: Red

Table 2. IC_{50} values and selectivity indices (SI) of crude extract and purified actinomycin D against cancer cell lines.

Test substances	MDA-MB-231 cells[a]		HeLa cells		Vero cells
	IC_{50} (µg mL^{-1})	SI	IC_{50} (µg mL^{-1})	SI	IC_{50} (µg mL^{-1})
**Crude extract**	12.30 ± 3.22[b]	1.02	3.93 ± 0.47	3.19	12.52 ± 2.64
**Purified actinomycin D**	7.85 ± 2.77	1.08	3.20 ± 0.58	2.64	8.46± 0.82
**Commercial actinomycin D**	6.62 ± 3.35	1.12	2.45 ± 0.13	3.03	7.43± 0.75
**Doxorubicin hydrochloride**	6.25 ± 1.14	15.92	1.95 ± 0.20	51.02	99.48± 10.46

[a]MDA-MB-231 cells; human breast cancer cell line, HeLa cells; human cervical carcinoma cell line, Vero cells; African green monkey kidney cell line.

[b]Values are expressed as mean ± standard deviation of the three replicates.

Therefore, it is interesting to note that actinomycin D, a major compound from *Streptomyces kunmingensis* BH111 isolated from beehive, showed toxicity against tested bacteria and hasd moderate anticancer properties with moderate toxicity in normal cells which imply that the beehives act as a promising resource for actinomycete isolation of valuable bioactive compound production.

Formatted: Font color: Red

Discussion

Here, we identified a known antibiotic; actinomycin D produced by a *Streptomyces kunmingensis* BH111 isolated from the beehive. The compound was confirmed based on ESI-HRMS and NMR data as well as its bioactivity. Actinomycin D is one of over 30 chromopeptide lactone antibiotics. All naturally occurring actinomycins share a common phenoxazone core structure but differ in their amino acid composition within the peptide side chains [27].

These antibiotics are produced by various species of *Streptomyces*[28] and *Micromonospora* [29] as part of a complex mixture of actinomycins [30, 31]. Actinomycin D yield varies significantly among different *Streptomyces* species. For instance, *Streptomyces parvulus*, *S. felleus*, and *S. regensis* produce 152, 20, and 12 mg mL^{-1} of actinomycin D, respectively. Notably, *Streptomyces parvulus* primarily produces actinomycin D [32, 33]. However, most can produce only small quantities of actinomycin D. This is the first report of isolation of *Streptomyces kunmingensis* BH111 from beehive, which can produce actinomycin D at 39.86 mg g^{-1} of crude extract which was a high-yield production under nonoptimized cultivation conditions. Based on the phylogenetic tree in Fig 2a, the isolate BH111 appears to be closely related to *S. kunmingensis* strains DSD1764 and DSD250. This close relationship is likely due to their shared origin from the same node, which typically indicates membership within the same genus. Previously, *S. kunmingensis* has never been reported to produce actinomycin D. However, *Streptomyces kunmingensis* YIM121234 has been reportedly isolated from soil, and could produce many bioactive compounds for example: endophenazine A and 5,6-dihydrouracil which exhibited cytotoxic activities towards human breast adenocarcinoma cell line MCF-7 with IC_{50} values of 20.23 ± 1.37 and 28.98 ± 1.58 µg mL^{-1} [34]. The phenoxazone chromophore, a key structural element of actinomycins, displayed a fascinating similarity to the phenazine skeleton found in

endophenazine. The isolation of a microbial strain that produces actinomycin D as its exclusive secondary metabolite would offer significant advantages for large-scale production. This approach presents a potentially more cost-effective and labor-efficient alternative to traditional methods for developing high-yielding strains. These methods often involve genetic manipulation techniques like mutation [35], DNA shuffling [36], or media optimization [37], followed by complex bioreactor design and operation [38]. A strain that naturally produces actinomycin D as a major compound would offer significant advantages, streamlining downstream processing and potentially simplifying bioreactor configurations. This translates to reduced production costs and labor requirements. This highlights the importance of screening and identifying such exclusive ~~producer strains~~producer strains for commercially relevant antibiotics like actinomycin D. In this study, *Streptomyces kunmingensis* BH111 was isolated from beehive which produced the known compound; actinomycin D as a major compound. Actinomycin D is a compound of interest, but its isolation is often hindered by its production as part of a mixture of actinomycins. This study investigated isolate BH111 as a potential source of actinomycin D production. TLC analysis revealed a favorable profile for isolate BH111. In contrast to the typical production of multiple actinomycins, isolate BH111 appears to primarily produce actinomycin D as the major compound. This finding suggests that isolate BH111 may be a suitable candidate for simplified purification of actinomycin D due to the minimal presence of interfering compounds. Actinomycin D obtained from the isolate BH111 possessed antibacterial and anticancer activities similar to commercial actinomycin D (Thermo Fisher Scientific, Massachusetts, USA).

Conclusions

The newly isolated strain BH111 was isolated from beehive of *Apis florea*. This isolate was identified as *Streptomyces kunmingensis* BH111 based on physiological, morphological

Formatted: Highlight

Formatted: Highlight

characteristics, and 16S rDNA gene analysis. The major bioactive metabolite isolated from the culture medium was characterized as actinomycin D. It showed high activity against test organisms such as *B. subtilis*, *B. cereus, S. aureus*, *S. epidermidis*, MRSA, and HeLa and MDA-MB-231 cells. Encouragingly, this study not only reinforces the understudied relationship between actinomycetes and antibiotic production but also expands the search for antibiotics towards complex microbial environments like beehive. Is it summary of the study or conclusion? Conclusion should focus on key aim/s/ of the study!

Formatted: Font color: Red, Not Highlight

Formatted: Font color: Red

Acknowledgements

We would like to thank Ms. Wenuka Rairak at 99/59 Moo 4, Thamakham Sub-district, Muang district, Kanchanaburi Province, 71000, Thailand for collection of beehives from The Circle House Kanchanaburi (14.02762 N, 99.51710 E), Thailand.

Author Contributions

Designed the experiments: TT. Performed the experiments: TT TC and BR. Analyzed the data: WSP. Wrote the paper: TT BR.

References

1. Selim MSM, Abdelhamid SA, Mohamed SS. Secondary metabolites and biodiversity of actinomycetes. J Genet Eng Biotechnol. 2021;19(1):72. doi: 10.1186/s43141-021-00156-9.
2. Promnuan Y, Kudo T, Chantawannakul P. Actinomycetes isolated from beehives in Thailand. World J Microbiol Biotechnol. 2009;25(9):1685–9. Doi: 10.1007/s11274-009-0051-1.
3. Khan KA, Al-Ghamdi AA, Ghramh HA, Ansari MJ, Ali H, Alamri SA, et al. Structural diversity and functional variability of gut microbial communities associated with honey bees. Microb Pathog. 2020;138:103793. doi: 10.1016/j.micpath.2019.103793.
4. Grubbs KJ, May DS, Sardina JA, Dermenjian RK, Wyche TP, Pinto-Tomás AA, et al. Pollen *Streptomyces* Produce Antibiotic That Inhibits the Honey Bee Pathogen *Paenibacillus larvae*. Front Microbiol. 2021;12:632637. doi: 10.3389/fmicb.2021.632637.

5. Santos-Beneit F, Ceniceros A, Nikolaou A, Salas JA, Gutierrez-Merino J. Identification of Antimicrobial Compounds in Two *Streptomyces* sp. Strains Isolated From Beehives. Front Microbiol. 2022;13:742168. doi: 10.3389/fmicb.2022.742168.
6. Promnuan Y, Promsai S, Pathom-Aree W, Meelai S. *Apis andreniformis* associated Actinomycetes show antimicrobial activity against black rot pathogen (*Xanthomonas campestris* pv. *campestris*). PeerJ. 2021;9:e12097. doi: 10.7717/peerj.12097.
7. De Simeis D, Serra S. *Actinomycetes*: A Never-Ending Source of Bioactive Compounds-An Overview on Antibiotics Production. Antibiotics. 2021;10(5):483. doi: 10.3390/antibiotics10050483.
8. Shirling EB, Gottlieb D. Methods for characterization of *Streptomyces* species. Int J Syst Bacteriol. 1966;16(3):313–40. doi: 10.1099/00207713-16-3-313.
9. Taechowisan T, Mungchukeatsakul N, Phutdhawong WS. Antimicrobial resistance pattern of *Staphylococcus aureus* strains isolated from clinical and hospital environment specimens and their correlation with PCR-based approaches. Res J Microbiol. 2018;13(2):100–18 doi: 10.3923/jm.2018.100.118.
10. Hockett KL, Baltrus DA. Use of the Soft-agar Overlay Technique to Screen for Bacterially Produced Inhibitory Compounds. J Vis Exp. 2017;119:e55064. doi: 10.3791/55064.
11. Taechowisan T, Lumyong S. Activity of endophytic actinomycetes from roots of *Zingiber officinale* and *Alpinia galanga* against phytopathogenic fungi. Ann Microbiol. 2003;53(3):291–8.
12. Taechowisan T, Puckdee W, Phutdhawong WS. *Streptomyces zerumbet*, a species from *Zingiber zerumbet* (L.) Smith and isolation of its bioactive compounds. Adv Microbiol. 2019;9(3):194–219. doi: 10.4236/aim.2019.93015.
13. Jesionek W, Móricz ÁM, Alberti Á, Ott PG, Kocsis B, Horváth G, et al. TLC-Direct Bioautography as a Bioassay Guided Method for Investigation of Antibacterial Compounds in Hypericum perforatum L. J AOAC Int. 2015;98(4):1013-20. doi: 10.5740/jaoacint.14-233.

14. Becker B, Lechevalier MP, Gordon RE, Lechevalier HA. Rapid differentiation between *Nocardia* and *Streptomyces* by paper chromatography of whole-cell hydrolysates. Appl Microbiol. 1964;12(5):421-3. doi: 10.1128/am.12.5.421-423.1964.

15. Boone CJ, Pine L. Rapid method for characterization of actinomycetes by cell wall composition. Appl Microbiol. 1968;16(2):279-84. doi: 10.1128/am.16.2.279-284.1968.

16. Castillo UF, Strobel GA, Ford EJ, Hess WM, Porter H, Jensen JB, et al. Munumbicins, wide-spectrum antibiotics produced by *Streptomyces* NRRL 30562, endophytic on *Kennedia nigriscans*. Microbiology. 2002;148(9):2675-85. doi: 10.1099/00221287-148-9-2675.

17. Taechowisan T, Chuen-Im T, Phutdhawong WS. Antioxidant and Antibacterial Properties of 1,3-Dihydroxy-,2', 2'-Dimethylpyrano-(5,6)-Xanthone from *Streptomyces* sp. SU84. Pak J Biol Sci. 2024;27(3):132-41. doi: 10.3923/pjbs.2024.132.141.

18. Yamamoto S, Harayama S. PCR Amplification and Direct Sequencing of gyrB Genes with Universal Primers and Their Application to the Detection and Taxonomic Analysis of Pseudomonas putida Strains. Appl Environ Microbiol. 1995;61(10):3768. doi: 10.1128/aem.61.10.3768-3768.1995.

19. Tamura K, Stecher G, Kumar S. MEGA11: Molecular Evolutionary Genetics Analysis Version 11. Mol Biol Evol. 2021;38(7):3022-7. doi: 10.1093/molbev/msab120.

20. Pfaller MA, Jones RN, Walter DH. Proposed quality control guidelines for National Committee for Clinical Laboratory Standards Susceptibility Tests using the veterinary antimicrobial agent tiamulin. Diagn Microbiol Infect Dis. 2001;40(1-2):67-70. doi: 10.1016/s0732-8893(01)00239-5.

21. van Meerloo J, Kaspers GJ, Cloos J. Cell sensitivity assays: the MTT assay. Methods Mol Biol. 2011;731:237-45. doi: 10.1007/978-1-61779-080-5_20.

22. Arison BH, Hoogsteen K. Nuclear magnetic resonance spectral studies on actinomycin D. Preliminary observations on the effect of complex formation with 5'-deoxyguanylic acid. Biochemistry. 1970;9(20):3976-83. doi: 10.1021/bi00822a016.

23. Lackner H. Zur sekundär- und tertiärstruktur der actinomycine, III selektiv im (α)- oder (β)-peptidlactonring deuterierte actinomycine C_1(D). Tetrahedron Letters. 1971; 12(24):2221–6 DOI 10.1016/S0040-4039(01)96825-X.

24. Hollstein U, Breitmaier E, Jung G. ^{13}C nuclear magnetic resonance study of actinomycin D. J Am Chem Soc. 1974;96(26):8036-40. doi: 10.1021/ja00833a033.

25. Praveen V, Tripathi CKM, Bihari V, Srivastava SC. Production of actinomycin-D by a new isolate, *Streptomyces sindenensis* . Ann Microbiol. 2008;58(1):109-14. doi: 10.1007/BF03179453.

26. Zhang X, Ye X, Chai W, Lian XY, Zhang Z. New Metabolites and Bioactive Actinomycins from Marine-Derived *Streptomyces* sp. ZZ338. Mar Drugs. 2016;14(10):181. doi: 10.3390/md14100181.

27. Mauger AB, Thomas WA. NMR studies of actinomycins varying at the proline sites. Organic Magnetic Resonance. 1981;17(3):186-90. doi: 10.1002/mrc.1270170311.

28. Kurosawa K, Bui VP, VanEssendelft JL, Willis LB, Lessard PA, Ghiviriga I, et al. Characterization of *Streptomyces* MITKK-103, a newly isolated actinomycin X2-producer. Appl Microbiol Biotechnol. 2006;72(1):145-54. doi: 10.1007/s00253-005-0240-2.

29. Wagman GH, Marquez JA, Watkins PD, Gentile F, Murawski A, Patel M, et al. A new actinomycin complex produced by a *Micromonospora* species: fermentation, isolation, and characterization. Antimicrob Agents Chemother. 1976;9(3):465-9. doi: 10.1128/AAC.9.3.465.

30. Korzybski Z, Kowszyk-Gindifer Z, Kurylowicz W. Antibiotics, Origin, Nature and Properties. Oxford-London-Edinburgh-New York-Toronto-Sydney-Paris-Braun-schweig: Pergamon Press, Warszawa: Polish Scientific Publishers, 1967, 2 Vols.

31. Praveen V, Tripathi CKM, Bihari V. Studies on optimum fermentation conditions for actinomycin-D production by two new strains of *Streptomyces* spp. *Med Chem Res*. 2008;17:114-22. doi: 10.1007/s00044-007-9042-7.

32. Williams WK, Katz E. Development of a chemically defined medium for the synthesis of actinomycin D by *Streptomyces parvulus*. Antimicrob Agents Chemother. 1977;11(2):281-90. doi: 10.1128/AAC.11.2.281.

33. Sousa MFVQ, Lopes CE, Pereira JrN. Development of a bioprocess for the production of actinomycin-D. Braz J Chem Eng.2002;19(3):277-85. doi: 10.1590/S0104-66322002000300002.

34. Wei HX, Fang XW, Xie XS, Zhang SP, Jiang Y, Wu SH. Secondary Metabolites of a Soil-Derived *Streptomyces kunmingensis*. Chem Nat Compd. 2017;53(4):794-6. doi: 10.1007/s10600-017-2125-x.

35. Praveen V, Tripathi CKM, Bihari V, Srivastava SC. Production of actinomycin-D by the mutant of a new isolate of *Streptomyces sindenensis*. Braz J Microbiol. 2008;39(4):689-92. doi: 10.1590/S1517-838220080004000017.

36. Crameri A, Raillard SA, Bermudez E, Stemmer WP. DNA shuffling of a family of genes from diverse species accelerates directed evolution. Nature. 1998;391(6664):288-91. doi: 10.1038/34663.

37. Praveen V, Tripathi D, Tripathi CKM, Bihari V. Nutritional regulation of actinomycin-D production by a new isolate of *Streptomyces sindenensis* using statistical methods. Indian J Exp Biol. 2008;46(2):138-44. PMID 18335813.

38. Dalili M, Chau PC.1988. Production of actinomycin D with immobilized *Streptomyces parvullus* under nitrogen and carbon starvation conditions. Biotechnol Lett. 1988;10(5):331-6. doi: 10.1007/BF01026160.

Supporting information

S1 Fig. Mass spectrometry analysis of the Actinomycin D. HRMS (ESI+) m/z (rel. int.) : $(M + Na)^+$ (100%), calcd. for $C_{62}H_{86}N_{12}O_{16}{}^{23}Na$, 1277.6182; found, 1277.6315 , $(M + H)^+$ (55%) calcd. for $C_{62}H_{87}N_{12}O_{16}$, 1255.6363; found, 1255.6508.

S2 Fig. ^{1}H NMR spectrum of Actinomycin D (in $CDCl_3$-*d*).

S3 Fig. ^{13}C NMR spectrum of Actinomycin D (in $CDCl_3$-*d*).

S4 Fig. Molecular structure of Actinomycin D.

S5 Fig. Beehives of *Apis florea* bees collected from the Circle House Kanchanaburi (14.02762 N, 99.51710 E), Thailand.

S6 Fig. Inhibitory effects of *Streptomyces kunmingensis* BH111. After 24 hours of incubation at 37°C, the inhibition zones were examined to check for antibacterial activity using the soft-agar overlay technique. *Staphylococcus aureus* TISTR885 (a) and *Bacillus cereus* TISTR687 (b) were added to the 7-day-old preculture of *Streptomyces kunmingensis* BH111 on ISP-2 medium.

S7 Fig. Morphological characteristics of *Streptomyces kunmingensis* BH111. After 21 days of growth on the ISP-2 agar at 32°C incubation; bar = 10 µm.

Figure 1

Click here to access/download;Figure;Fig1.png

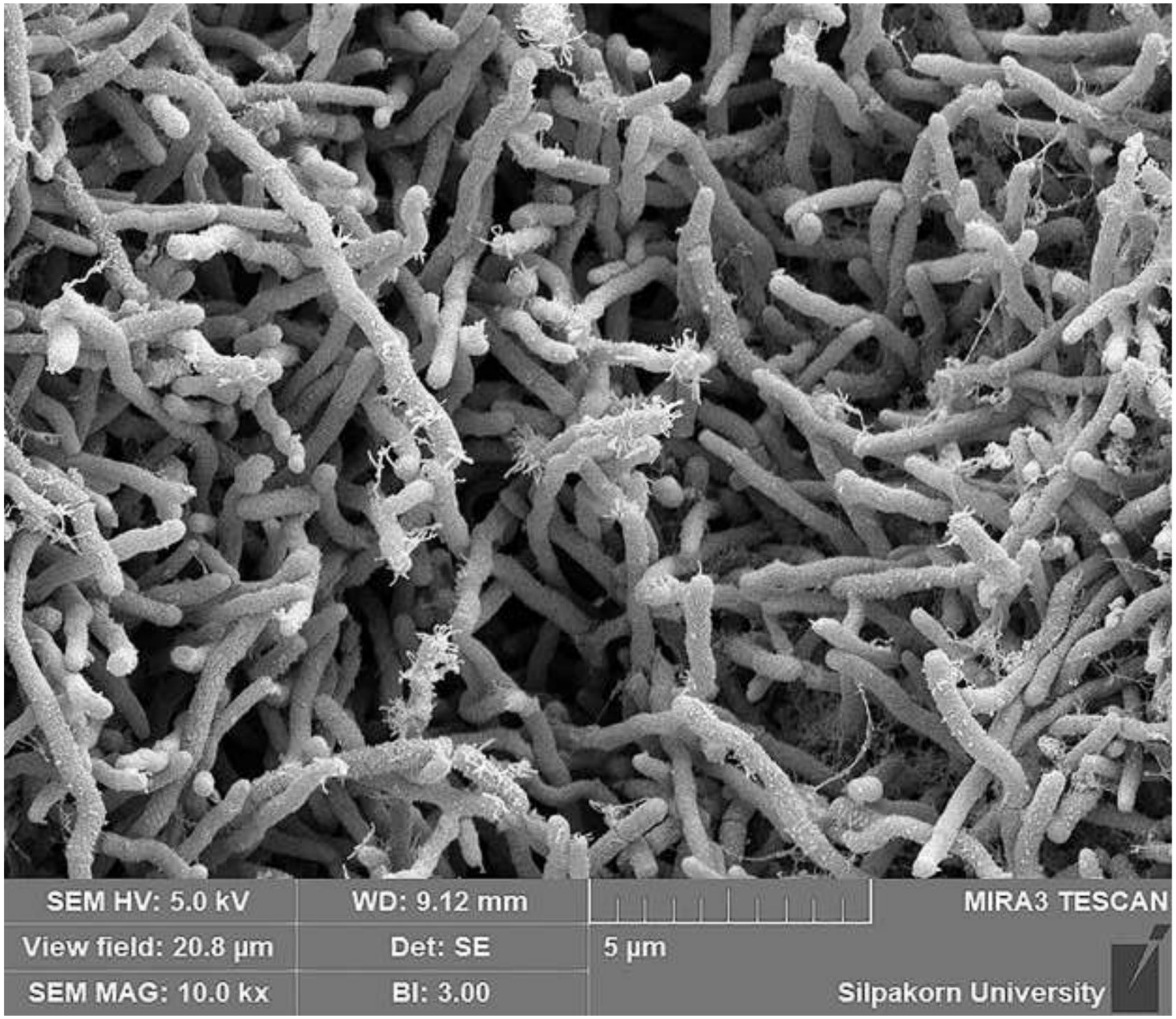

Figure 2a

Click here to access/download;Figure;Fig2a.png

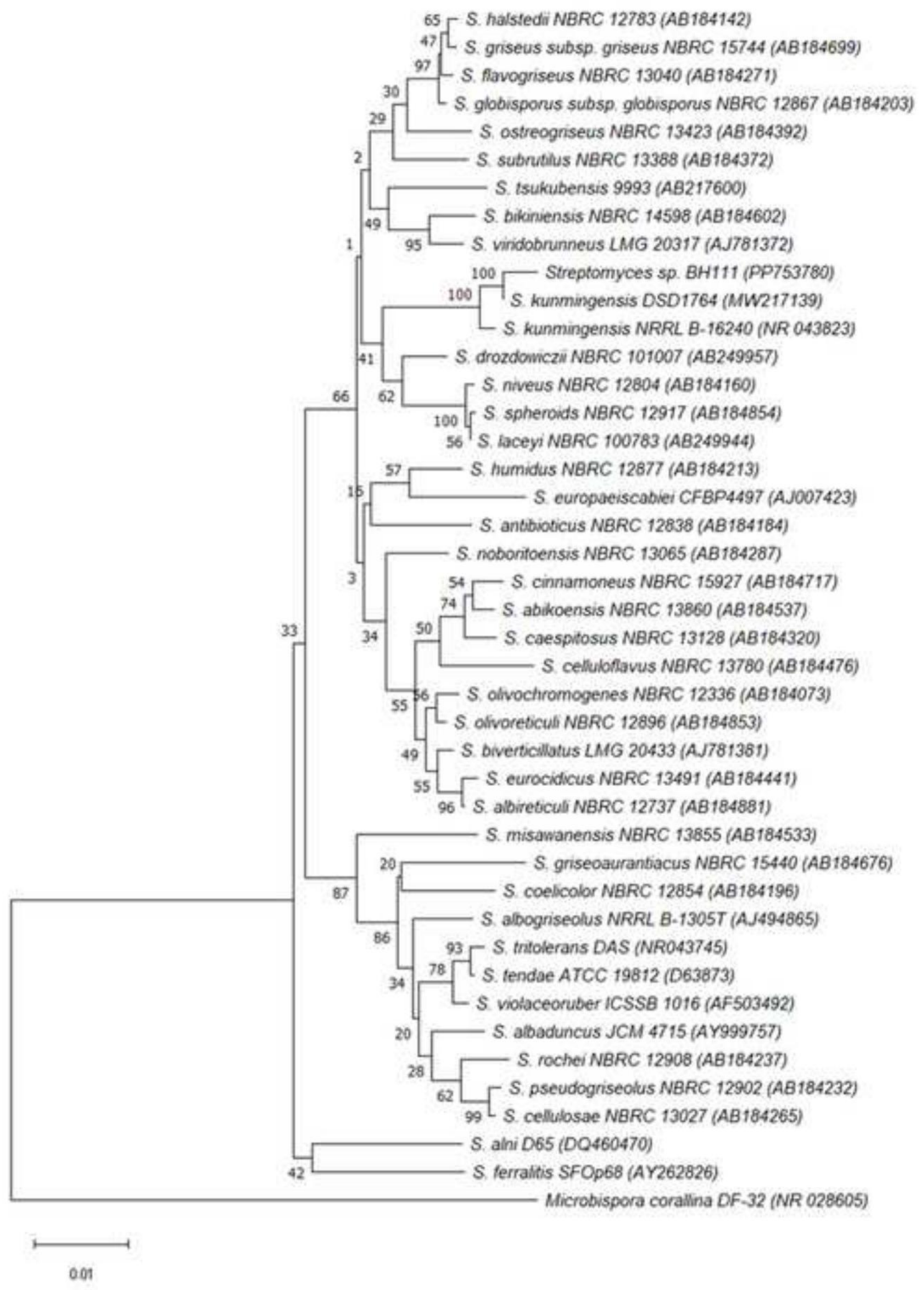

Figure 2b

Click here to access/download;Figure;Fig2b.png

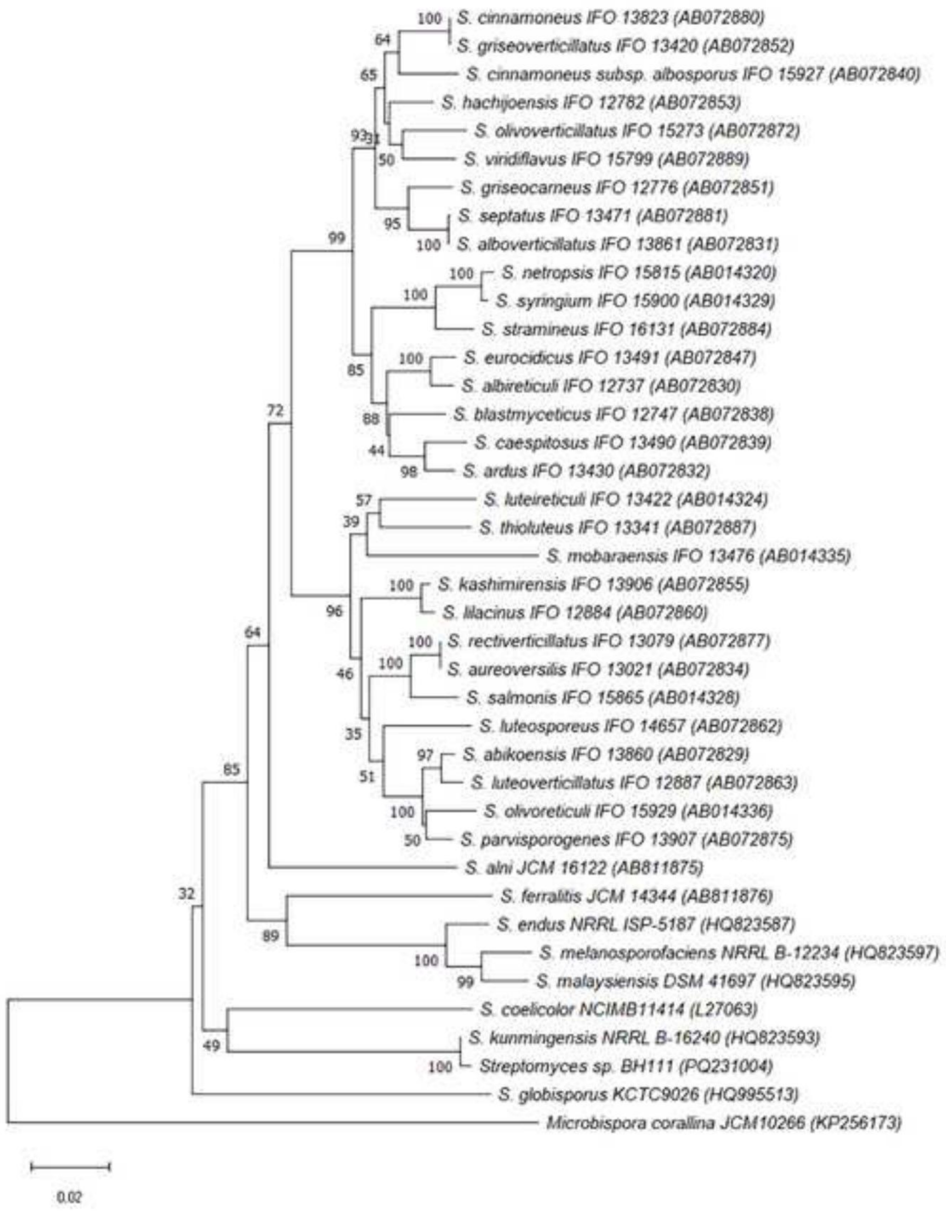

S1 Figure

Click here to access/download;Figure;S1__Fig.jpg

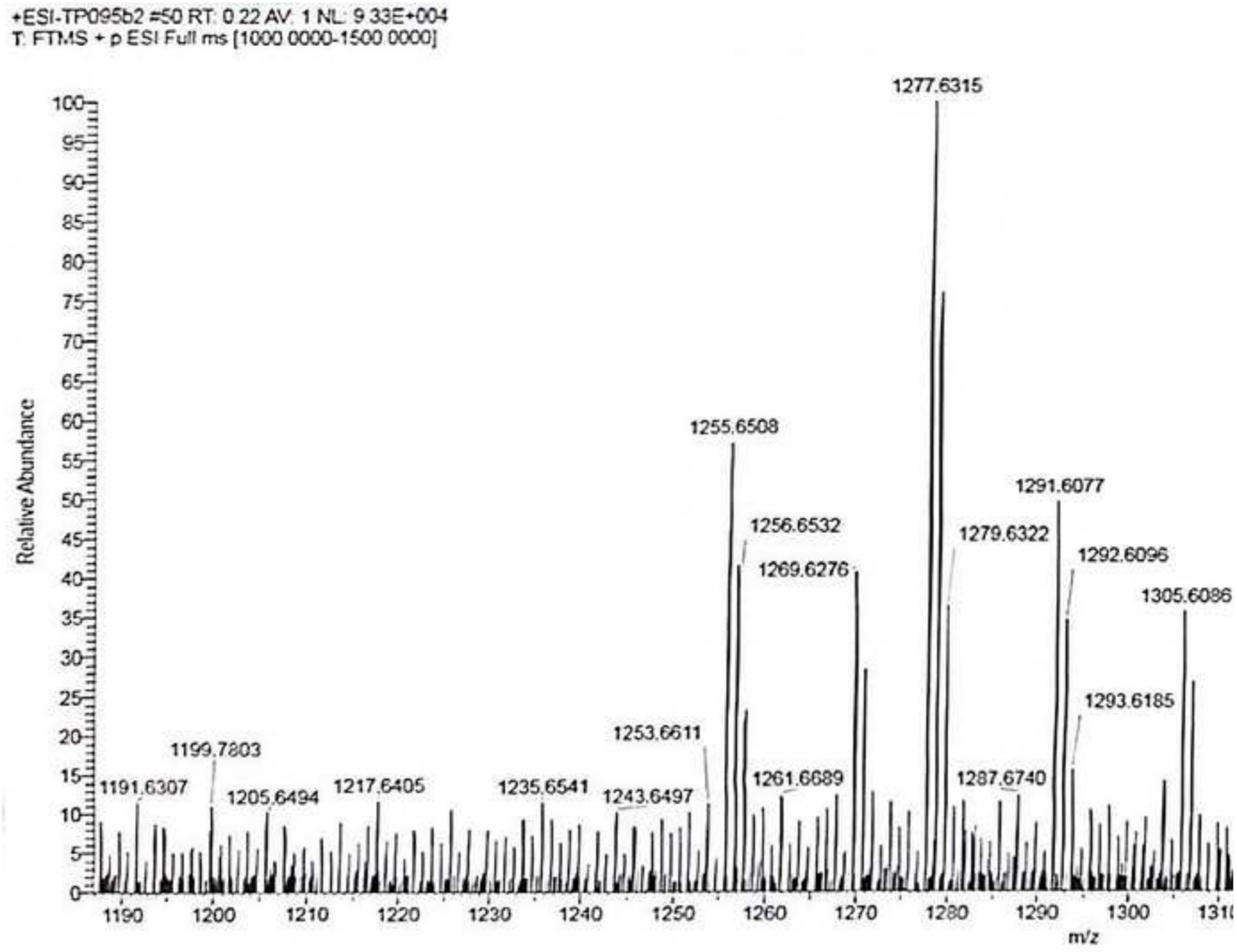

S2 Figure

Click here to access/download;Figure;S2__Fig.jpg

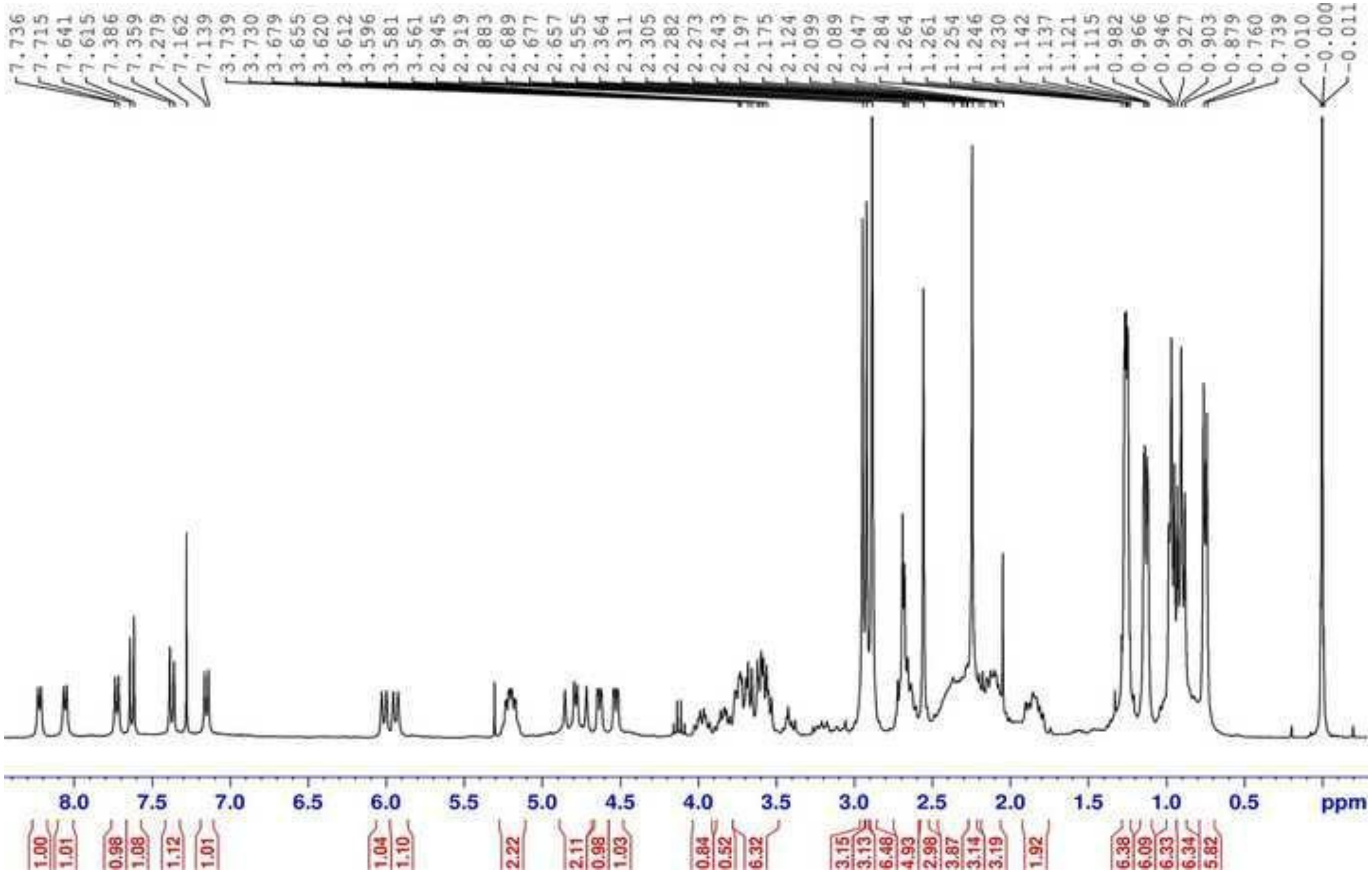

S3 Figure

Click here to access/download;Figure;S3__Fig.jpg

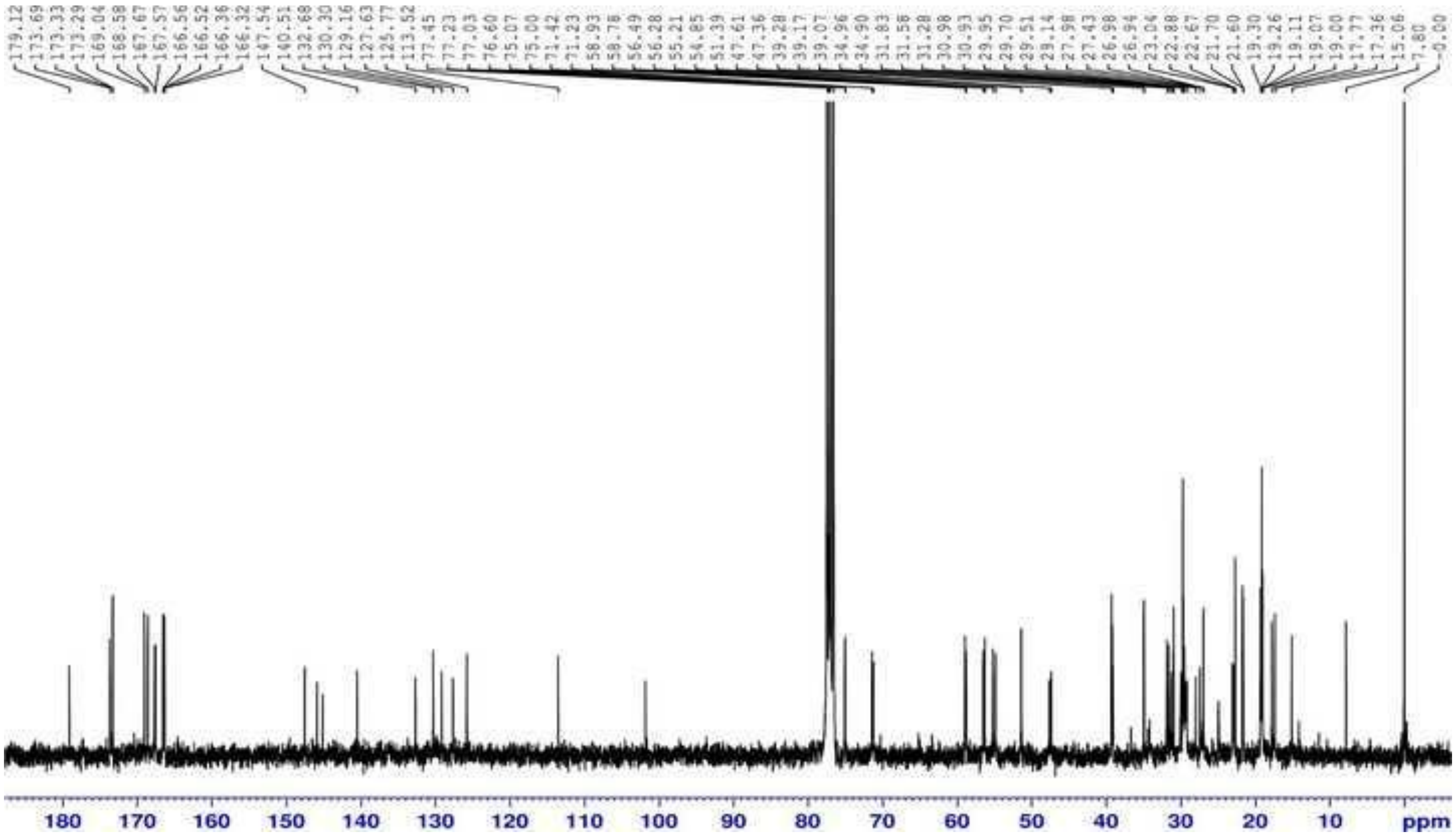

S4 Figure

Click here to access/download;Figure;S4__Fig.jpg

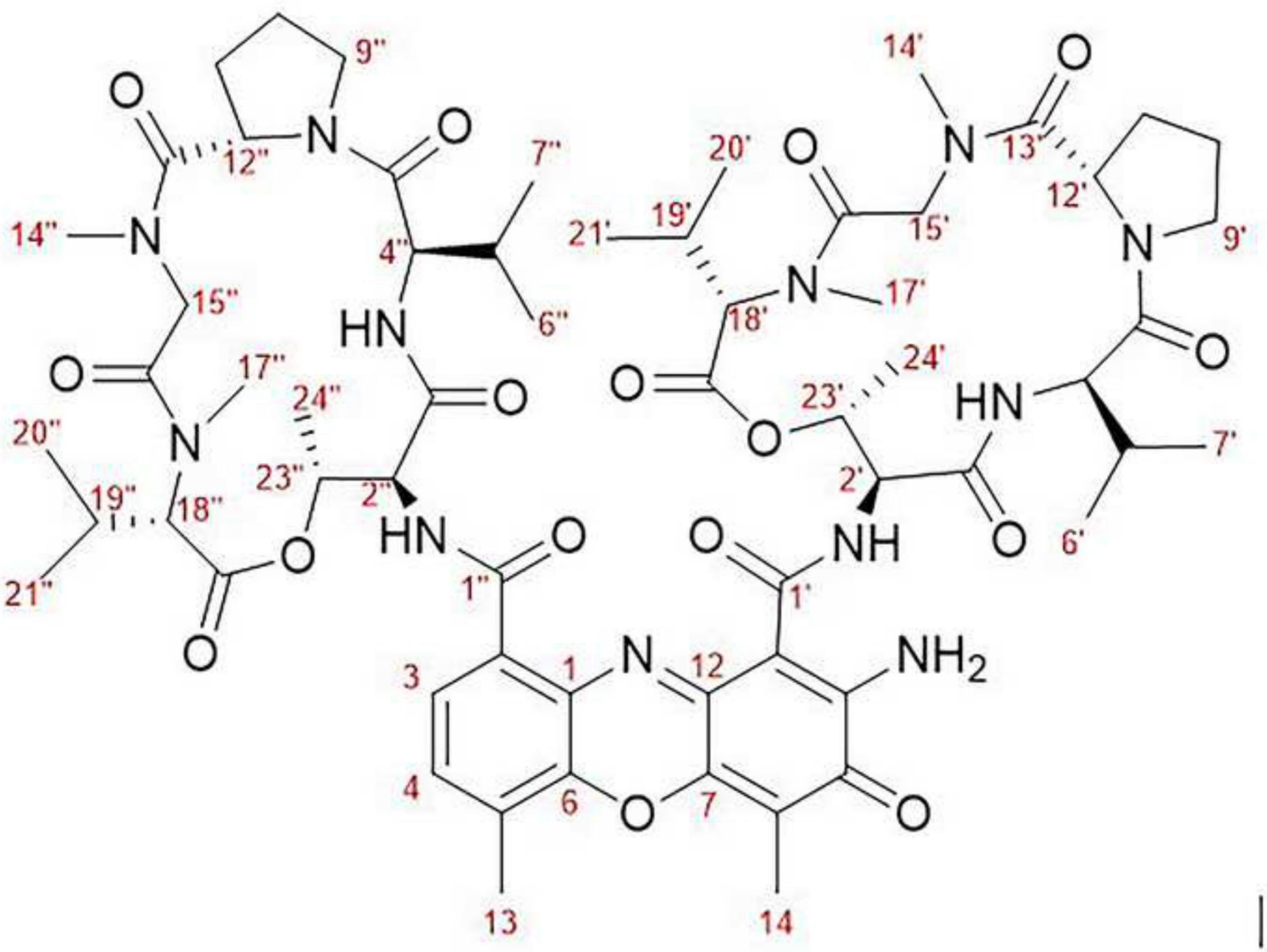

S5a Figure

Click here to access/download;Figure;S5_Fig_a.tif

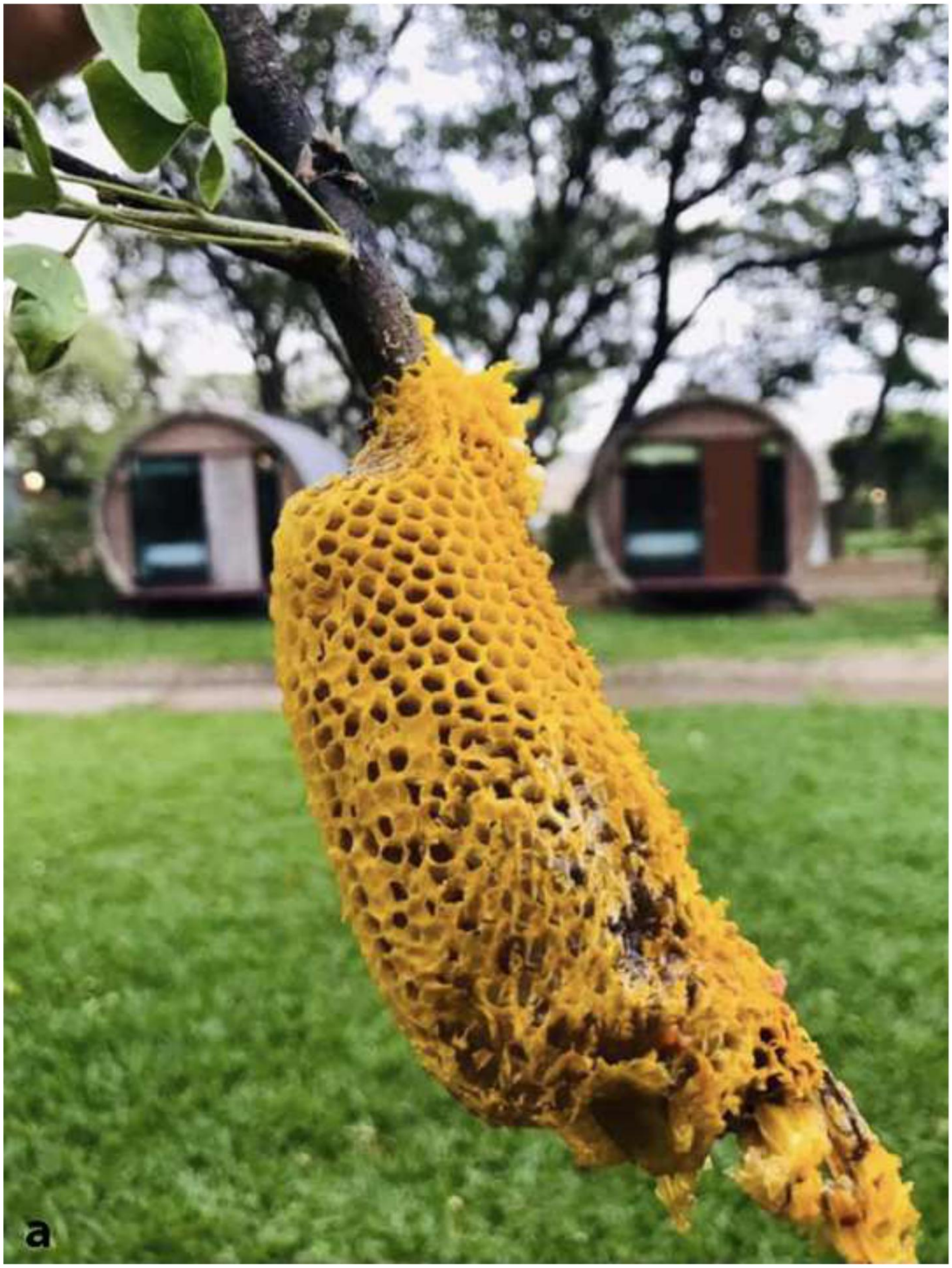

S5b Figure

Click here to access/download;Figure;S5_Fig_b.tif

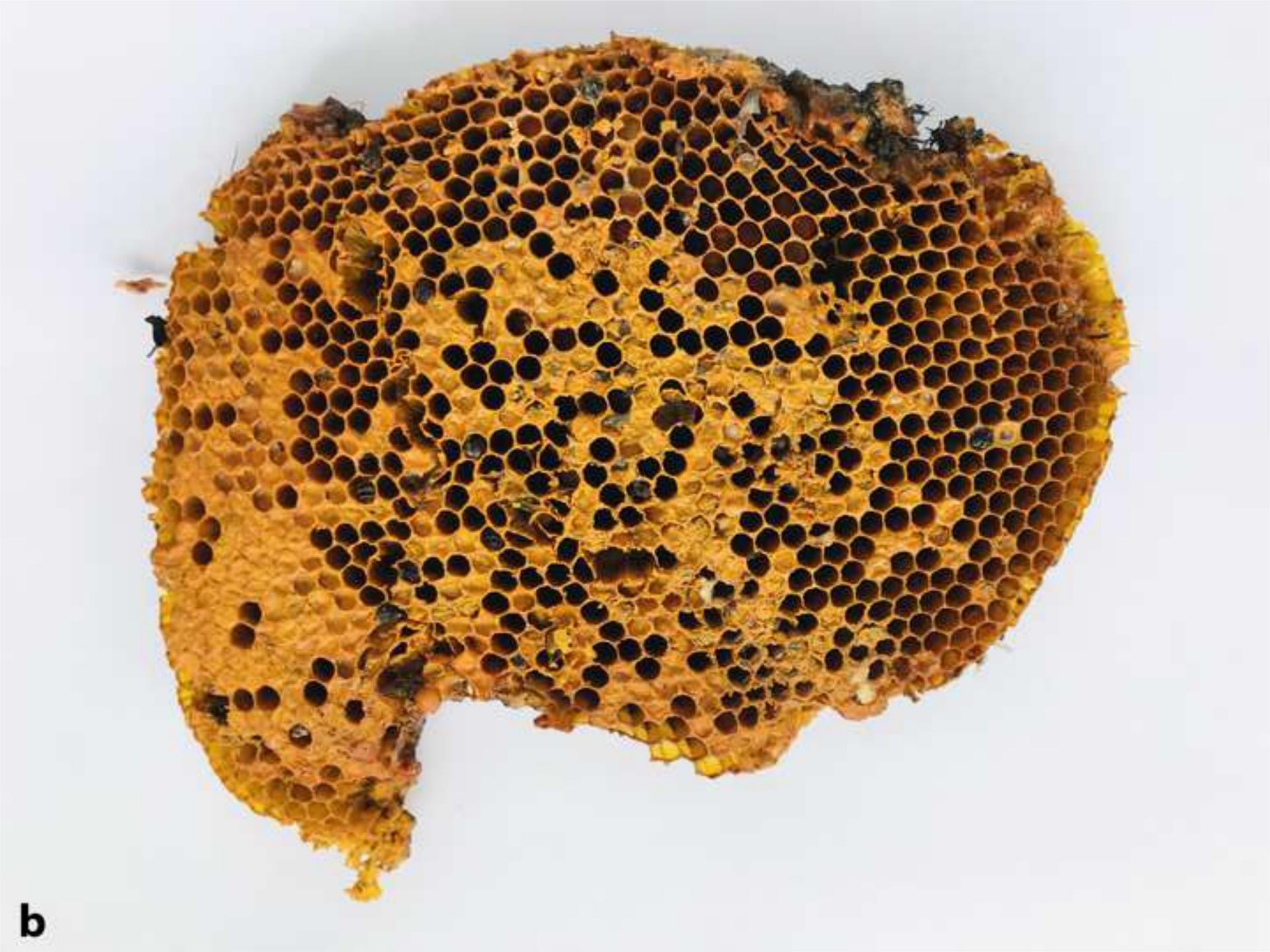

S6a Figure

Click here to access/download;Figure;S6_Fig_a.tif

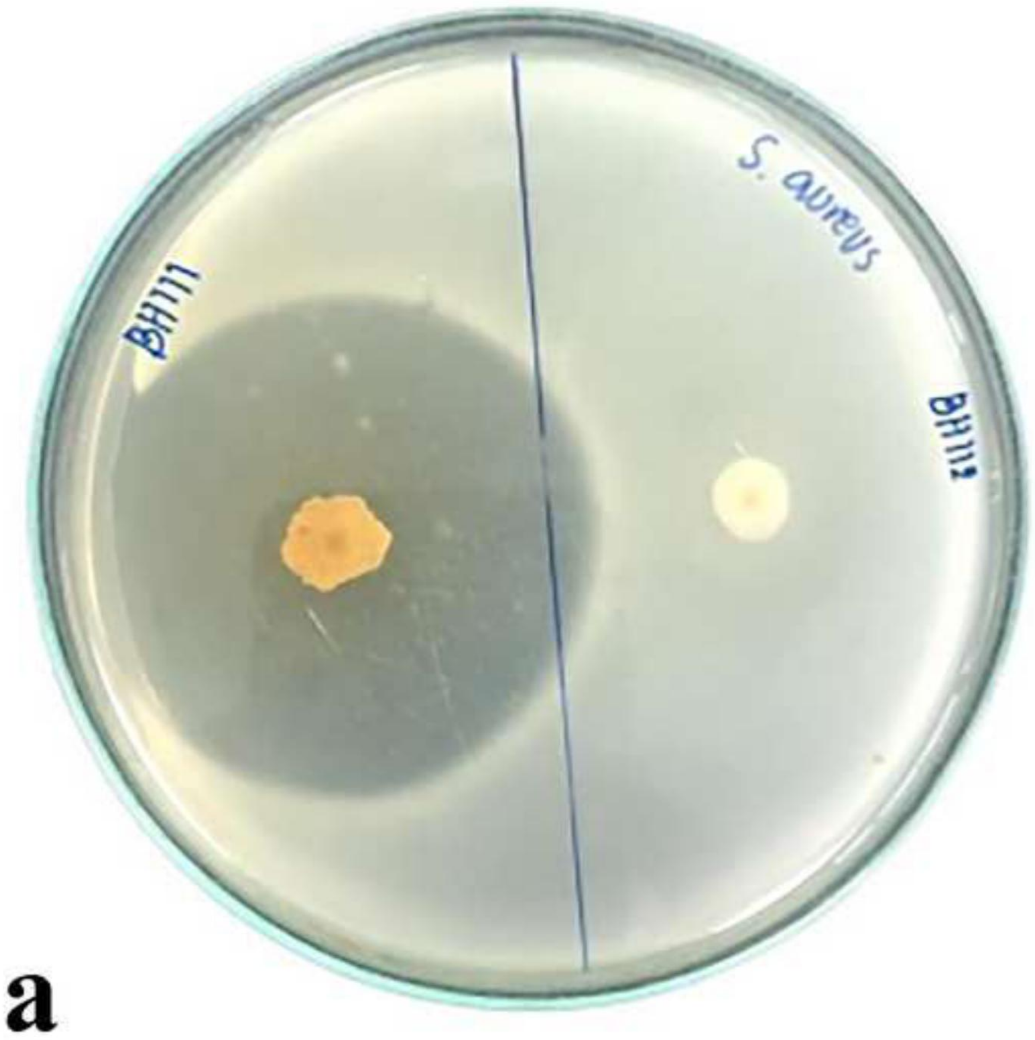

S6b Figure

Click here to access/download;Figure;S6_Fig_b.tif

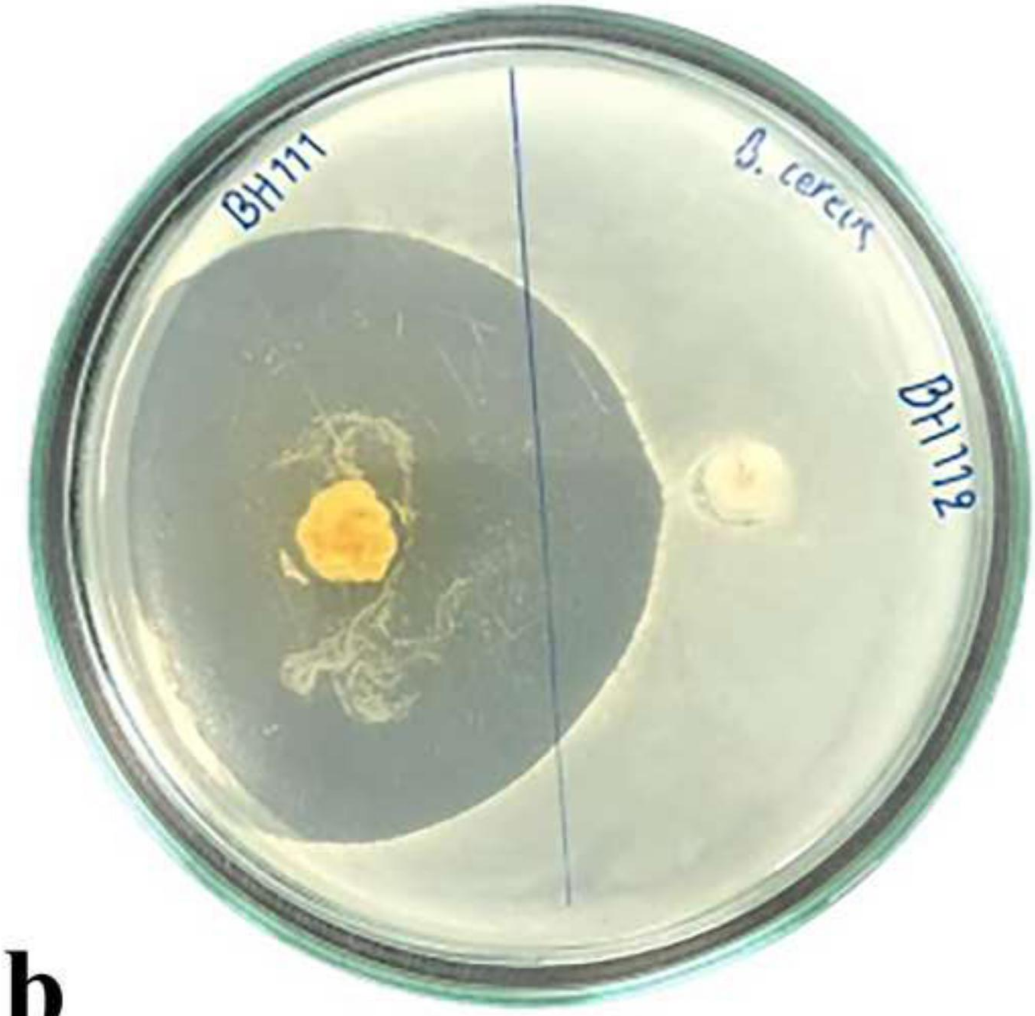

b

S7 Figure

Click here to access/download;Figure;S7_Fig.jpg

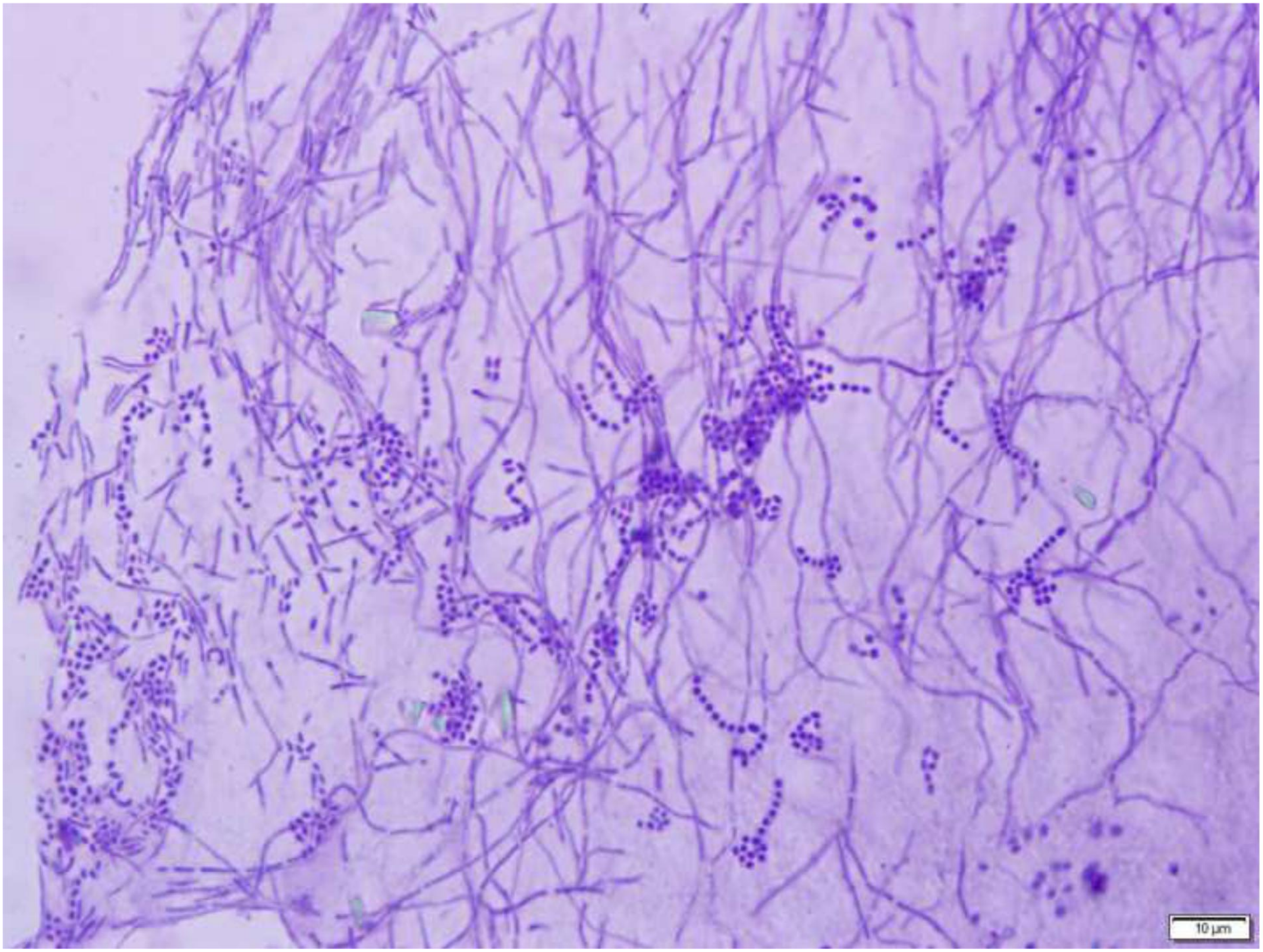

Supporting Information

Click here to access/download
Supporting Information
S1 Table.docx
