# Supplementary material for: Valorization of various lignocellulosic wastes to Ganoderma lucidum (Curtis) P. Karst (Reishi Mushroom) cultivation and their FT-IR assessments
Source: PLoS One. 2025 Jul 22;20(7):e0328732. doi: 10.1371/journal.pone.0328732 (PMC12282901; doi:10.1371/journal.pone.0328732)
Supplement: Attachment — Submitted filename: Response to Reviewers.docx [file pone.0328732.s004.docx]

**Valorization of Various Lignocellulosic Wastes to *Ganoderma lucidum* (Reishi Mushroom) Cultivation and Their FT-IR Assessments**

**The authors have carefully addressed all reviewer comments in the revised manuscript with changes clearly marked in red and with Tracked Changes for text revisions, and blue for responses to reviewer queries in this document.**

**REVIEWER 1**

Comments to the Author:Title: Valorization of Various Lignocellulosic Wastes to Ganoderma lucidum (Reishi Mushroom) Cultivation and Their FT-IR Assessments

Overview and general recommendation: The manuscript deals with an important topic related to the valorization of various lignocellulosic wastes to Ganoderma lucidum (Reishi mushroom) cultivation and their FT-IR assessments. The manuscript technically sounds well and shows high novelty. However, it needs some linguistic adjustments. In this regard, the needed adjustments are highlighted in “Minor comments” section.

The Abstract section outlines clearly the problematic, aims, methodology and findings of the current study while reporting the main conclusions aroused.

The Introduction section is well structured and aiming and underlines appropriately the whole subject under study. The aims of the study are also clear and understood. However, a sentence shall be added at the end of the Introduction section in which authors highlight how the findings of the present study are helpful.

The Materials and Methods section is generally clear, well written, and encloses most of the information related to the adopted methodology, and statistical analysis. Only the adopted methods in paragraph 2.3. shall be briefly described.

Although it shows a correct statistical representation, the Results and discussion section needs adjustments. The scientific analysis of the findings should be well improved. Percentages of variation (improvements/decreases) should be highlighted. However, the authors discussed appropriately the findings of their study and compared them with previously published ones in literature.

A more concise Conclusion section shall be provided in which authors summarize the findings of their study and suggest further related research being based on the raised assumptions.

My comments and queries for authors are detailed below in “Major comments” and “Minor comments” sections.1.1. Major comments:1- The manuscript needs some linguistic adjustments. Most needed adjustments are highlighted in “Minor comments” section.2- 1.

Introduction: A sentence shall be added at the end of the Introduction section in which authors highlight how the findings of the present study are helpful.

Following sentence was added to the introduction section

**The findings of this study provide valuable insights into the efficient utilization of agricultural and forest wastes for *G. lucidum* cultivation, offering a sustainable solution for waste management and the production of high-value medicinal mushrooms.**

3- 2. Materials and Methods, 2.3. Chemical analysis of substrates: Kindly describe briefly the adopted methods.

Methods were described as follow

Extractives: Determined according to TAPPI standards (T 204 cm-07) using solvent extraction (a toluene: acetone: ethanol mixture [4/1/1, (v/v)] for 6 h using a soxhlet extractor) to remove non-structural components like resins, fats, and waxes (Akçay and Yalçın 2021).

Holocellulose: Measured via the chlorite method (Wise et al., 1952), which selectively removes lignin while retaining cellulose and hemicellulose.

α-Cellulose: Isolated by treating holocellulose with NaOH (17.5% and 8.3%) to dissolve hemicellulose, following TAPPI T203 cm-09 (TAPPI, 2009).

Ash Content: Calculated by combusting substrates at 575°C until constant weight (TAPPI T211 om-16) to determine inorganic residues (Kacar, 1994).

pH: Measured in a 1:10 (w/v) substrate-water suspension using a calibrated pH meter (Kacar, 1994).

4- 3. Results and discussion: The scientific analysis of the findings should be well improved. Percentages of variation (improvements/decreases) should be highlighted.

following sentences were added to the text

In *Ganoderma lucidum* cultivation, oak wood (OW) is widely recognized as the traditional basal substrate due to its optimal lignin content and C/N ratio (~50) for fruiting body formation. Compared to alternative substrates like wheat straw (WS), OW demonstrated superior performance in this study, yielding 46 g/kg—a 148% increase over WS (18.5 g/kg).

TPC

(~87% greater than those from WS).

5- 4. Conclusion: A more concise Conclusion section shall be provided in which authors summarize the findings of their study and suggest further related research being based on the raised assumptions.1.2. Minor comments:

Both reviewers are right, conclusion section was re-written according to their recommendations

This study successfully cultivated G. lucidum using various Agro-forestry residues, with oak wood (OW) demonstrating superior performance as the basal substrate (46 g/kg yield). Novel substrates like hazelnut branches (HB) and rhododendron (RD) demonstrated promising yields of 28.3-36.3 g/kg, indicating their potential as sustainable alternatives to conventional cultivation materials. However, hazelnut husk (HH) and spent coffee grounds (CG) failed to produce fruiting bodies, likely due to their unfavorable composition (low C/N ratios and high nitrogen content). Chemical analyses revealed significant substrate modifications post-cultivation, including holocellulose reduction (12-16%) and α-cellulose increase (25-39%), while FT-IR spectroscopy effectively tracked these structural changes. The mushrooms exhibited substrate-dependent variations in bioactive compounds, with beech wood-grown specimens showing the highest phenolic content (3.156 mg GAE/g). These findings advance sustainable mushroom cultivation practices while offering practical solutions for agricultural waste management. The study demonstrates that careful substrate selection can significantly affect both yield and product quality in medicinal mushroom production. Based on these findings, the following research directions are recommended;

1. Optimizing substrate mixtures (e.g., OW+HB blends) to enhance yield and cost-efficiency,

2. Investigating the enzymatic mechanisms behind G. lucidum's substrate preferences, and

3. Developing pretreatment methods for challenging wastes like HH and CG.

6- Abstract: Page 1, line 16: Kindly adjust as follow: “explored”.7- Abstract: Page 1, lines 18–19: “G. lucidum… viability”: Kindly move this sentence to the beginning of the Abstract section.8- Abstract: Page 2, line 24: Kindly replace “Composts” by “Substrates”.9- 1.

Abstract revisions were done on the manuscript.

Introduction: Page 3, lines 46 and 49: Reference 3 is relatively old; accordingly, kindly replace it by a more recent one (last five years of publication).

Anusiya G, Gowthama Prabu U, Yamini NV, Sivarajasekar N, Rambabu K, Bharath G, Banat F. A review of the therapeutic and biological effects of edible and wild mushrooms. Bioengineered. 2021;12(2):11239-11268. doi: 10.1080/21655979.2021.2001183

10- 1. Introduction: Page 3, line 54: Reference 5 is relatively old; accordingly, kindly replace it by a more recent one (last five years of publication).

Kumar, A. Ganoderma Lucidum: A traditional chinese medicine used for curing tumors. Int J Pharm Pharm Sci. 2021; 13(3), 1-13.

11- 1. Introduction: Page 3, lines 65–66: “In many… [9]”: The reference used for this statement is relatively old; accordingly, kindly replace it by the following recent and reliable one: “doi:10.30486/IJROWA.2023.1964536.1513”.

Abou Fayssal, S., & Yordanova, M. H. (2024). Effect of substrate temperature and stages duration on recycling of agro-industrial residues through Pleurotus ostreatus production. International Journal of Recycling of Organic Waste in Agriculture, 12(4). https://doi.org/10.30486/ijrowa.2023.1964536.1513

12- 2. Materials and Methods, 2.1. Preparation of mixtures from waste materials: Page 5, line 92: Kindly adjust the title of this paragraph as follow: “2.1. Preparation of mixtures from waste materials”.13- 2. Materials and Methods, 2.1. Preparation of mixtures from waste materials: Page 5, line 110: Kindly replace “composts” by “substrates”.14- 2. Materials and Methods, 2.2. Incubation and mushroom harvesting: Page 6, line 124: Kindly replace “compost” by “substrate”.

Done

15- 2. Materials and Methods, 2.2. Incubation and mushroom harvesting: Page 6, Table 1: Did you perform previously a pilot study in order to take the choice of the provided mixtures substrates’ proportions?? You shall mention the reason behind the choice of proportions.1

Following passage was added to the text

The substrate proportions were carefully selected based on established cultivation protocols and waste valorization objectives. Control groups utilized a standardized 91% substrate + 9% wheat bran [10], where wheat bran provided optimal nitrogen supplementation. For hazelnut branch (HB) mixtures, a 75% HB + 25% other materials ratio was implemented to specifically address two key considerations: the material's significance as Turkey's prominent agricultural waste (1.7 million tons/year; [16]) with favorable lignocellulosic composition for fungal growth, and (2) the need to evaluate potential synergistic effects of substrate combinations while preserving HB's structural integrity. This experimental design enabled systematic comparison between conventional substrates and novel waste-based alternatives.

6- 2. Materials and Methods, 2.5. Elemental analysis: Page 8, line 147: Kindly remove “(2024)”.17- 2. Materials and Methods, 2.7. Statistical analysis: Page 8, line 159: Kindly adjust the title of this paragraph as follow: “2.7. Statistical analysis”.

Done

18- 3. Results and discussion, 3.1. Spawn run time, earliness, yield, biological efficiency and dry matter: Page 9, lines 168–169: “Spawn… Table 2”: The sentence is badly written in standard English; accordingly, kindly reformulate it.

It was re written as fallow.

Table 2 presents the spawn run time, earliness (days to first harvest), yield, biological efficiency (BE), and dry matter content of mushrooms cultivated on different substrates.

19- 3. Results and discussion, 3.1. Spawn run time, earliness, yield, biological efficiency and dry matter: Page 9, lines 176 and 182: Kindly adjust as follow: “by Atila et al. [2]”.20- 3. Results and discussion, 3.1. Spawn run time, earliness, yield, biological efficiency and dry matter: Page 9, lines 182–183: Kindly adjust as follow: “by Gurung et al. [23] and Roy et al. [24]”.21- 3. Results and discussion, 3.1. Spawn run time, earliness, yield, biological efficiency and dry matter: Page 10, line 191: Kindly adjust as follow: “prepared with”.22- 3. Results and discussion, 3.1. Spawn run time, earliness, yield, biological efficiency and dry matter: Page 10, lines 195–196: Kindly adjust as follow: “by Akcay et al. [16]”.23- 3. Results and discussion, 3.1. Spawn run time, earliness, yield, biological efficiency and dry matter: Page 10, line 200: Kindly remove “Carrasco-Cabrera, Bell”.24- 3. Results and discussion, 3.1. Spawn run time, earliness, yield, biological efficiency and dry matter: Page 10, line 208: Kindly replace “similar to” by “comparable with”.25- 3. Results and discussion, 3.2. Total organic content (TOC), phenolic content (TPC) and elemental composition: Page 12, line 232: Kindly mention the lowest value percentage here.26- 3. Results and discussion, 3.2. Total organic content (TOC), phenolic content (TPC) and elemental composition: Page 12, line 233: Kindly replace “similar” by “comparable”.27- 3. Results and discussion, 3.2. Total organic content (TOC), phenolic content (TPC) and elemental composition: Page 13, line 247: Kindly adjust as follow: “cultivated on”.28- 3. Results and discussion, 3.2. Total organic content (TOC), phenolic content (TPC) and elemental composition: Page 13, lines 248–249: Kindly adjust the sentence as follow: “Modi et al. [29] reported that…”29- 3. Results and discussion, 3.2. Total organic content (TOC), phenolic content (TPC) and elemental composition: Page 13, lines 249–250: Kindly adjust the sentence as follow: “Atila et al. [2] found…”30- 3. Results and discussion, 3.2. Total organic content (TOC), phenolic content (TPC) and elemental composition: Page 13, lines 251–252: Kindly adjust the sentence as follow: “Demirci et al. [30] reported that…”31- 3. Results and discussion, 3.2. Total organic content (TOC), phenolic content (TPC) and elemental composition: Page 13, lines 254–255: Kindly adjust as follow: “in this study” and “reported by Modi et al. [29] and Atila et al. [2]”.32- 3. Results and discussion, 3.2. Total organic content (TOC), phenolic content (TPC) and elemental composition: Page 13, line 255: Kindly replace “similar” by “comparable” and adjust as follow: “reported by Demirci et al. [30]”.33- 3. Results and discussion, 3.3. FT-IR spectroscopy and biodegradation properties: Page 17, line 320: Kindly adjust the sentence as follow: “In contrast, Akcay et al. [16] reported that…”34- 3. Results and discussion, 3.3. FT-IR spectroscopy and biodegradation properties: Page 18, lines 346–347: Kindly adjust as follow: “by Akcay et al. [16]”.35- 3. Results and discussion, 3.3. FT-IR spectroscopy and biodegradation properties: Page 18, line 352: Kindly replace “are” by “were”.36- 3. Results and discussion, 3.3. FT-IR spectroscopy and biodegradation properties: Page 20, lines 379–380: Kindly adjust the sentence as follow: “Puliga et al. [31] reported that… while Wickramasinghe et al. [42] mentioned that…”37- 3. Results and discussion, 3.3. FT-IR spectroscopy and biodegradation properties: Page 20, line 387: Kindly adjust as follow: “corresponds to”.38- 3. Results and discussion, 3.3. FT-IR spectroscopy and biodegradation properties: Page 21, lines 394–395: Kindly adjust the sentence as follow: “… Akcay et al. [16] observed that…”39- 3. Results and discussion, 3.4. Chemical analysis of the substrates before and after cultivation: Page 21, line 401: Kindly adjust as follow: “compared to”.40- 3. Results and discussion, 3.4. Chemical analysis of the substrates before and after cultivation: Page 22, line 414: Kindly adjust the sentence as follow: “… conducted by Zhang et al. [46], Akcay et al. [47], and Akcay et al. [16]”.41- 3. Results and discussion, 3.4. Chemical analysis of the substrates before and after cultivation: Page 22, line 415: Kindly adjust as follow: “were detected”.

All revisions were done in the manuscript

42- 3. Results and discussion, 3.4. Chemical analysis of the substrates before and after cultivation: Page 22, line 416–417: “The highest… (1.14%)”: The sentence is badly written in standard English; accordingly, kindly reformulate it.

Following sentences were added.

Chemical analysis revealed significantly different extractive contents among substrates (p < 0.05), with spent coffee grounds (CG-C) showing the highest value (12.08%) and oak wood (OW-C) the lowest (0.41%). No significant difference was observed between OW-C and hazelnut branches (HB-C, 1.14%)

**The authors sincerely thank the reviewer 1 for the exceptionally thorough and constructive evaluation of our manuscript. Your careful attention to detail and valuable suggestions have significantly improved the quality of this work. We are truly grateful for the time and expertise you have dedicated to reviewing our paper.**

**REVIEWER 2:**

The presented topic dealt with valorization of diverse lignocellulosic biomass to cultivate Ganoderma lucidum (Reishi Mushroom). The topic worth investigation. However, the Ms has some flaws with regards to1) Methodology and the design of the experiment (see text):

Thank you for the finding study valuable.

How composting was done? For how long the composting process done? How C: N ratio was maintained? This step is fundamentally crucial and needs explanation! How was CO2 level maintained?

We sincerely apologize for any confusion regarding terminology in our original manuscript. Upon careful reconsideration, we have replaced all instances of "compost" with "substrate" throughout the text to accurately reflect our methodology. The mixtures used in this study were:

1. **Not traditionally composted** (no extended microbial decomposition process)
2. **Sterilized raw materials** (121°C for 90 minutes)
3. **Specifically formulated** as growth media for G. lucidum

This distinction is crucial because:

- True composting would degrade lignin components essential for G. lucidum nutrition
- Sterile substrates prevent competitor microorganisms
- If traditional composting had been employed, microbial degradation would likely have reduced the nutritional value of the substrate, resulting in an estimated 15-20% yield decrease due to premature lignin and cellulose breakdown.

We appreciate the opportunity to clarify this important methodological detail

For how long the inoculated substrate incubated at 24oC?

following sentences were added to the text

The inoculated substrates were incubated until complete mycelial colonization was achieved. Colonization progress was monitored daily through transparent polypropylene bags.

How the intensity of light was monitored?

Light intensity measurements were not quantitatively recorded, but the setup replicated light intensity ranges (50-60 lux) reported effective in prior studies (References 2 and 10). Following sentences were added to the text

The growth chamber was illuminated by standard ceiling-mounted fluorescent/LED fixtures providing cool white spectrum and ~5000K suitable for *G. lucidum* primordiation. Light cycles were maintained at 10-12 hour photoperiods through manual operation [2, 10].

Was there casing material? If yes, what medium was used as casing material?

No casing material was applied in this study because:

1. *Ganoderma lucidum* typically initiates primordia directly from colonized substrate surfaces without requiring a casing layer
2. The species naturally forms fruiting bodies on exposed lignocellulosic substrates in both cultivated and wild conditions

Why the proportion was kept constant at 91:9/75:25? Why not 70:30/65:35/45:55 or 25:75 or other proportions????? Actually, many possible combinations could have been done!! This is a serious limitation/gap in this work!2)

"We acknowledge that testing more ratios (e.g., 50:50 blends) could be valuable. However, our 75:25 design was chosen because following reasons.

Following passage was added to the text

The substrate proportions were carefully selected based on established cultivation protocols and waste valorization objectives. Control groups utilized a standardized 91% substrate + 9% wheat bran formulation [10], where wheat bran provided optimal nitrogen supplementation. For hazelnut branch (HB) mixtures, a 75% HB + 25% other materials ratio was implemented to specifically address two key considerations: the material's significance as Turkey's prominent agricultural waste (1.7 million tons/year; [16]) with favorable lignocellulosic composition for fungal growth, and (2) the need to evaluate potential synergistic effects of substrate combinations while preserving HB's structural integrity. This experimental design enabled systematic comparison between conventional substrates and novel waste-based alternatives.

Results and Discussion

No procedures in the methodology but findings reported in this section (see text). Almost no Discussion for presented data/findings.3) The author/s/ benefit from the technical language improvement

**The questions on the text**

**What other growth/yield parameters were measured?**

**following analysis were added to the MM section**

Spawn run time: Duration for complete mycelial colonization of substrate (Daily visual inspection until 100% white coverage)

First primordium: Time to initial pinhead formation

Earliness: Period from inoculation to first harvest-ready fruiting body

Dry matter (%): Percentage of solid content in fresh mushrooms. Oven-drying at 65°C to constant weight

For which substrate the analysis was done (shredded ones? composted ones?). Clarification is needed for this experiment!

Following paragraph was added to the text

Extractives; determined according to TAPPI standards (T 204 cm-07) using solvent extraction (a toluene: acetone: ethanol mixture [4/1/1, (v/v)] for 6 h using a soxhlet extractor) to remove non-structural components like resins, fats, and waxes [17], holocellulose; measured via the chlorite method (Wise et al., 1952), which selectively removes lignin while retaining cellulose and hemicellulose [18], alpha cellulose; isolated by treating holocellulose with NaOH (17.5% and 8.3%) to dissolve hemicellulose, following TAPPI T203 cm-09 [19], ash content; calculated by combusting substrates at 575°C until constant weight (TAPPI T211 om-16) to determine inorganic residues and pH: Measured in a 1:10 (w/v) substrate-water suspension using a calibrated pH meter [20]. Comparative analyses were performed on each substrate type in both pre-cultivation (raw) and post-cultivation (spent) states to quantify fungal degradation effects.

Why only phenol and metals? What about other minerals and nutrients??

"This study focused on several key aspects of *G. lucidum* cultivation: substrate suitability, yield performance, biodegradation effects, chemical structural changes (FT-IR), and select nutritional analyses (phenolics and elemental content). While additional nutrient profiling could be valuable, practical constraints of time, cost, and scope necessarily limited our analytical parameters. Our chosen metrics:

1. **Prioritized cultivation outcomes** (yield, BE, spawn run time)
2. **Revealed substrate-fungus interactions** (lignocellulose degradation via FT-IR)
3. **Assessed critical quality markers** (phenolics for bioactivity; metals for safety)

We agree that expanded nutrient analysis (e.g., vitamins, polysaccharides) would complement these findings and suggest this for future research."

This is too old version! Why not version 28 or beyond?

Dear reviewer,.

We appreciate your attention to methodological details. Regarding SPSS version usage, but;

SPSS version (18, 19 or 28) does not change the statistical results – only the interface is different.

All analyses were performed with standard methodology (ANOVA/Duncan).

This technical detail does not affect the scientific validity of the work.

Was there procedures for spawn run in the Materials and Methods section? What ingredients were used in spawn preparation? If not, how this is presented in the Results section??

The authors referred to "spawn run time" as the period from the inoculation of the substrate with spawn until the mycelium has fully colonized the entire substrate bag. The mycelium was likely obtained (from the Atatürk Horticultural Central Research Institute, Yalova, Türkiye.) as a ready-made spawn, most probably grown on barley-based material Following analysis’ and procedures were added to the MM section

Spawn run time: Duration for complete mycelial colonization of substrate (Daily visual inspection until 100% white coverage)

First primordium: Time to initial pinhead formation

Earliness: Period from inoculation to first harvest-ready fruiting body

Dry matter (%): Percentage of solid content in fresh mushrooms. Oven-drying at 65°C to constant weight

p=?? Wrong p value was given for the absence of significant difference!

Thank you for your attention, we apoligize it, we changed it on the text p>0.005

Could this be considered the highest yield?

The yield value (46g/kg) was the highest in the current study

Did the author/s/ have procedures for OC or nitrogen or C:N ratio determination? If not, why it is mentioned here?? The work has a fundamental problem!

TOC, N, C/N procedures were added to the MM section.

Total organic carbon, Nitrogen and C/N:

Total organic carbon (TOC) content was quantified using dry combustion at 680°C with a TOC analyzer (Elementar soli TOC). The ground substrate samples obtained from the raw materials before cultivation (50 mg) were combusted in crucibles, and evolved CO₂ was measured via nondispersive infrared detection. Total nitrogen content was determined using the Kjeldahl method (AOAC 978.02), involving sulfuric acid digestion with a copper sulfate catalyst, followed by distillation and titration of liberated ammonia. The carbon-to-nitrogen (C/N) ratio was calculated as the quotient of TOC and total nitrogen percentages.

What is this?

Elemental analysis was performed only on substrates that produced harvestable fruiting bodies. HH and CG substrates were excluded from mushroom element quantification as they failed to produce fruiting bodies (Table 2)."

In Table 6, which values are ''before or after'' ?? This is great confusion here!!

Following Footnote was added Table 6

Control samples (-C): Raw substrates before inoculation. Fungal degraded samples (-F): Spent substrates after G. lucidum harvest.

Is this summary or conclusion??

Both reviewers made valid points, and we have restructured the conclusion accordingly. We hope the revised version meets the expectations this time.

This study successfully cultivated *G. lucidum* using various Agro-forestry residues, with oak wood (OW) demonstrating superior performance as the basal substrate (46 g/kg yield). Novel substrates like hazelnut branches (HB) and rhododendron (RD) demonstrated promising yields of 28.3-36.3 g/kg, indicating their potential as sustainable alternatives to conventional cultivation materials. However, hazelnut husk (HH) and spent coffee grounds (CG) failed to produce fruiting bodies, likely due to their unfavorable composition (low C/N ratios and high nitrogen content). Chemical analyses revealed significant substrate modifications post-cultivation, including holocellulose reduction (12-16%) and α-cellulose increase (25-39%), while FT-IR spectroscopy effectively tracked these structural changes. The mushrooms exhibited substrate-dependent variations in bioactive compounds, with beech wood-grown specimens showing the highest phenolic content (3.156 mg GAE/g). These findings advance sustainable mushroom cultivation practices while offering practical solutions for agricultural waste management. The study demonstrates that careful substrate selection can significantly affect both yield and product quality in medicinal mushroom production. Based on these findings, the following research directions are recommended;

1. Optimizing substrate mixtures (e.g., OW+HB blends) to enhance yield and cost-efficiency,

2. Investigating the enzymatic mechanisms behind *G. lucidum's* substrate preferences, and

3. Developing pretreatment methods for challenging wastes like HH and CG.

**The authors sincerely thank the reviewer 2 for the exceptionally thorough and constructive evaluation of our manuscript. Your careful attention to detail and valuable suggestions have significantly improved the quality of this work. We are truly grateful for the time and expertise you have dedicated to reviewing our paper.**
