# Supplementary material for: Valorization of various lignocellulosic wastes to Ganoderma lucidum (Curtis) P. Karst (Reishi Mushroom) cultivation and their FT-IR assessments
Source: PLoS One. 2025 Jul 22;20(7):e0328732. doi: 10.1371/journal.pone.0328732 (PMC12282901; doi:10.1371/journal.pone.0328732)
Supplement: Attachment — Submitted filename: Response to Reviewer.docx [file pone.0328732.s006.docx]

Reviewer #1: Comments to the Author:Title: Valorization of Various Lignocellulosic Wastes to Ganoderma lucidum (Reishi Mushroom) Cultivation and Their FT-IR AssessmentsOverview and general recommendation:Authors have made all needed improvements to their manuscript and are well thanked for that. Therefore, based on the overall evaluation of the manuscript, I find it well suitable for publication in current form.

We appreciate the positive feedback and are pleased that the reviewer finds our manuscript suitable for publication in its current form.

Reviewer #3: In this work by Caglar Akcay and colleagues, the cultivation of the medicinal mushroom Ganoderma lucidum was tested on various agricultural residues. The degradative capacity of G. lucidum on different substrates was assessed using Fourier-transform infrared (FTIR) spectroscopy. Furthermore, the chemical composition of both the substrates and the fruiting bodies obtained after cultivation was investigated.Overall, the manuscript requires substantial improvements, particularly in methodology, language and English style. Specifically:

Abstract

We thank the reviewer for their detailed and constructive comments. Below are our responses to each point:

Revisions are necessary to include results concerning the chemical composition of both the substrates and the harvested fruiting bodies.

Line 16: The full scientific name Ganoderma lucidum along with the authority should be provided at its first mention, both in the abstract and the main text. The common name "Reishi" should be inserted in parentheses immediately afterward and removed from line 18.Introduction –Line 40: The full name and authority for Ganoderma lucidum should be provided at first mention. Additionally, when the name appears at the beginning of a sentence (see line 56), it should be written out in full.

Updated to include the full scientific name with authority: *Ganoderma lucidum* (Curtis) P. Karst. The common name "Reishi" is now in parentheses at first mention.

Line 77: Replace “Turkiye” with “Turkey.”

Replaced with "Turkey" as requested

Lines 81–82: Insert the authority for Pleurotus ostreatus.

Added authority for Pleurotus ostreatus: (Jacq.) P. Kumm.

Lines 82–84: While the authors provided context for the selection of hazelnut branches as a substrate, no justification is given for the choice of rhododendron. Context should be added to support this choice.

Added context: *Rhododendron* sp. branches were selected due to their regional abundance as forestry waste, aligning with our goal to valorize underutilized lignocellulosic materials.

Materials and Methods –Line 97: Please insert the authority for Fagus orientalis.Lines 98–102: Move the substrate abbreviations to lines 96–98, where they are first mentioned.Line 102: Replace “Turkiye” with “Turkey.”

Lines 110–116: This section should be moved to the Introduction, as it explains the objectives of the study.

Actually, our aim here was to explain why we used these materials and their specific ratios in the study; it was a response to another reviewer. If possible, we would prefer to keep it here. Thank you for your suggestion

Line 127: Replace “Turkiye” with “Turkey.”Lines 127–128: Replace "compost" with "substrate."

Lines 138–139:

After colonization, were the bags opened to stimulate primordia formation, or were they kept closed until primordia emerged and then opened to promote primordia development and fruiting body expansion? Please clarify.

following sentence was added to the text

“All cultivation bags remained hermetically sealed during the entire process, with only the sterile cotton filter at the neck being removed to permit the directional growth and emergence of fruiting bodies”.

Lines 145–150: This information should be rewritten in a more discursive manner rather than presented as bullet point.

Rewritten as a discursive paragraph: Spawn run time was recorded as the duration for complete mycelial colonization, assessed via daily visual inspection. Earliness represented the period from inoculation to first harvest-ready fruiting body. Dry matter content was determined by oven-drying fresh mushrooms at 40°C to constant weight."

Line 154: What was the dry weight of the different formulated substrates? This is essential for calculating biological efficiency. Please provide this information.

Following sentence was added to the manuscript:

Substrates were initially weighed as dry materials (500 g per bag, except for high-volume CH and WS which were reduced to 250 g to accommodate bag capacity).

Lines 156–166: This paragraph should be written in a more discursive manner and should include more detailed information about the methods used.

Following text was added to the manuscript:

“The extractives content of both pre-cultivation control substrates (HB-C, HH-C, WS-C, RD-C, OW-C, BW-C, CH-C, CG-C) and post-cultivation fungal-degraded materials (HB-F, HH-F, WS-F, RD-F, OW-F, BW-F, CH-F, CG-F) was determined according to a modified TAPPI T 204 cm-17 standard. All substrate samples were first ground to 40-mesh particle size using a Wiley mill and then oven-dried at 103±2°C until constant weight was achieved. For the extraction process, precisely weighed 5 g aliquots of each dried sample were subjected to solvent extraction (toluene: acetone: ethanol mixture [4/1/1, (v/v)] in a Soxhlet apparatus for 6 hours, with the extraction cycles carefully monitored to ensure complete removal of soluble compounds. Following extraction, the solutions were vacuum-filtered through pre-weighed porosity-2 crucibles, and the retained residues were subsequently dried at 103±2°C for 12 hours. The extractives content was calculated gravimetrically by comparing the initial sample mass to the mass of insoluble residue after extraction, with all measurements performed in triplicate to ensure reproducibility[17].

The holocellulose content of the five substrate samples before and after cultivation (hazelnut pruning waste, hazelnut husk, rice husk, coffee grounds, and wheat straw) was determined using an optimized Wise and John chlorite method. Extractives-free 40-mesh samples (5 g), prepared by oven-drying at 103±2°C, were subjected to a four-stage delignification process in 250 mL Erlenmeyer flasks. Each reaction cycle consisted of adding 160 mL distilled water, 1.5 g sodium chlorite (NaClO₂), and 0.5 mL glacial acetic acid, followed by incubation at 78±1°C in a temperature-controlled water bath with continuous magnetic stirring. The mixture underwent vigorous shaking every 15 minutes during the 1-hour reaction period to ensure homogeneous treatment. After each cycle, fresh reagents (1.5 g NaClO₂ + 0.5 mL acetic acid) were added, with the complete process repeating four times to achieve thorough lignin removal. The resulting holocellulose was collected by vacuum filtration through pre-weighed porosity-2 glass crucibles, then sequentially washed with ice-cold distilled water (to remove residual acids) and acetone (to facilitate drying). The purified holocellulose was dried to constant weight at 103±2°C. The holocellulose content of the substrates (%) were determined relative to the initial full dry weight[18].

Alpha -cellulosecontent was determined following TAPPI T 203 cm-09 with modifications. Precisely 2 g of holocellulose was treated with 17.5% NaOH (10 mL initial + 2×5 mL additions at 5-min intervals) at 20.0±0.5°C for 30 min. After dilution with 33 mL distilled water and 60 min incubation, the mixture was filtered through porosity-2 crucibles. The residue was sequentially washed with 8.3% NaOH (100 mL), 10% acetic acid (15 mL), and distilled water (250 mL), then dried at 105°C to constant weight. Alpha-cellulose content was calculated gravimetrically relative to initial holocellulose mass, with triplicate measurements [19].

Ash content was calculated by combusting substrates at 575°C until constant weight (TAPPI T211 om-16) to determine inorganic residues. pH was measured in a 1:10 (w/v) substrate-water suspension using a calibrated pH meter”

Lines 192–196: Please provide additional details on how the obtained spectra were processed. How many replicates per treatment were conducted? The figures (Figures 1–9) suggest that baseline correction was not performed. To better highlight significant changes in the substrates before and after fungal growth, it is recommended that the authors include histograms of peak areas (%) processed using curve fitting in the region from 1800 to 1200 cm⁻¹.

Following text was added to the manuscript:

Line 249: FT-IR spectra were obtained using an IRPrestige-21 FT-IR Spectrophotometer (Shimadzu) equipped with a single-reflection ATR sampling module. The spectra were recorded in the range of 4000–400 cm^-1^ with a resolution of 4 cm^-1^ by averaging 32 scans. Spectral data in the region between 1800 and 800 cm^-1^ were processed using curve fitting analysis performed in the OriginPro 2013 software (OriginLab Corporation, Northampton, MA 01060 USA), by determining the area under each band. The FT-IR spectra were fitted using Gaussian-shaped bands. Optimal Gaussian curve fitting was determined when the best fit was achieved (reduced chi-square < 1×10^-6^) and based on the agreement between the calculated areas (R-square, R^2^ = 0.998–0.987 for substrates, R^2^ = 0.901-0.941 for mushrooms).

Using the Gaussian curve fitting procedure, peak areas for both the substrates and the fungi were analyzed, and the corresponding percentage histograms were incorporated into the revised Figures 1–9. As the histograms adequately represent the data, Table 5 has been omitted. Accordingly, the results presented between lines 433 and 631 have been revised in light of the updated histograms and spectral data.

Following text was added to the manuscript:

Line 433: The FT-IR spectra in the fingerprint region (1800–800 cm^-1^), which show the structural changes in hazelnut branches (HB), beech wood (BW), oak wood (OW), rhododendron branches (RD), corn husk (CH), wheat straw (WS), hazelnut husk (HH), and spent coffee grounds (CG) after fungal attack compared to the control samples (before fungal attack), along with the histograms of areas obtained through the Gaussian curve fitting procedure applied to the examined regions of these spectra, are given in Figs 1–8.

Line 446: **Fig 1. FT-IR results.** a) FT-IR spectra of HB-C and HB-F; b) histograms of FT-IR peak areas processed using Gaussian curve fitting.

**Fig 2. FT-IR results.** a) FT-IR spectra of BW-C and BW-F; b) histograms of FT-IR peak areas processed using Gaussian curve fitting.

**Fig 3. FT-IR results.** a) FT-IR spectra of OW-C and OW-F; b) histograms of FT-IR peak areas processed using Gaussian curve fitting.

**Fig 4. FT-IR results.** a) FT-IR spectra of RD-C and RD-F; b) histograms of FT-IR peak areas processed using Gaussian curve fitting.

**Fig 5. FT-IR results.** a) FT-IR spectra of WS-C and WS-F; b) histograms of FT-IR peak areas processed using Gaussian curve fitting.

**Fig 6. FT-IR results.** a) FT-IR spectra of CH-C and CH-F; b) histograms of FT-IR peak areas processed using Gaussian curve fitting.

**Fig 7. FT-IR results.** a) FT-IR spectra of HH-C and HH-F; b) histograms of FT-IR peak areas processed using Gaussian curve fitting.

**Fig 8. FT-IR results.** a) FT-IR spectra of CG-C and CG-F; b) histograms of FT-IR peak areas processed using Gaussian curve fitting.

Line 470-568:

The Gaussian curve fitting procedure applied to the FT-IR spectra of the substrates gave twelve Gaussian curves centered at 890, 1030, 1110, 1150, 1240, 1320, 1370, 1420, 1460, 1510, 1620, and 1730 cm^-1^. The percentage area of each band showed significant changes after fungal attack. The functional groups corresponding to these bands were examined in detail.

The bands observed between 1750 and 1720 cm^-1^ are attributed to hemicellulose degradation in lignocellulosic materials. This band indicates unconjugated C=O vibrations of acetyl, carboxylic acid, and uronic ester groups within the hemicellulose structure [31, 32]. Hemicellulose degradation caused by fungal attack resulted in a decrease in the area of this band (12) in all samples. The strong band observed at 1742 cm^-1^ in the CG-C sample is attributed to the C=O stretching vibrations of ester groups from kinic acid and lipids [33]. After the fungal attack, this band disappeared.

The bands between 1650 and 1600 cm^-1^ correspond to lignin degradation in lignocellulosic materials [31]. In this region, the deformation of O-H groups in the lignin structure (1640 cm^-1^), C=C and C=O stretching in the lignin aromatic chain (1630 cm^-1^), and C-O stretching vibrations in the lignin skeletal structure (1630 cm^-1^) overlapped, forming a broad band [16]. As a result of lignin degradation by *G. lucidum*, a significant increase in the area of this band (11) was observed in the HB, BW, CH, HH, and CG samples, whereas no notable change was detected in the OW, RD, and WS samples. This result may be associated with the selective lignin degradation by *G. lucidum* mycelia, depending on the type and structure of the substrate. The broad band observed at 1638 cm^-1^ in the CG-C sample is attributed to C=C stretching vibrations of lipids and fatty acids, C=O stretching vibrations of caffeine, and C=C, C=O, and C-O stretching vibrations of the lignin aromatic chain [34].

The bands between 1530 and 1500 cm^-1^ correspond to the aromatic C–O stretching vibrations of lignin [35]. Following fungal attack, the area of this band (10) increased in the CG sample, while it decreased in all other samples. Similarly, Akcay et al. [16] reported that *Pleurotus ostreatus* increased the visibility of the 1652 cm^-1^ band due to lignin degradation in coffee. However, in the present study, since fungal growth could not be achieved on the CG substrate, this may be associated with the lower impact of *G. lucidum* on lignin in spent coffee grounds compared to *Pleurotus ostreatus*.

The bands between 1465–1455 cm^-1^ and 1425–1410 cm^-1^ are attributed to C-H deformations of CH_2_ and CH_3_ groups in lignin and hemicellulose, as well as chlorogenic acids in coffee, and CH_2_ in-plane bending vibrations in cellulose and lignin, respectively [36–38]. After fungal attack, the area of the first band (9) decreased in the HB, BW, OW, RD, and CG samples, while it slightly increased in the WS, CH, and HH samples. The area of the second band (8), however, decreased in all samples. A similar result was observed for CG in Pleurotus ostreatus mycelia [16].

The bands between 1375–1360 cm^-1^ and 1330–1310 cm^-1^ correspond to symmetric and asymmetric C-H deformation vibrations of cellulose and hemicellulose, O-H deformation of kinic acid, CH₂ in-plane bending vibrations at the sixth carbon of crystalline cellulose, and C-H deformation of kinic acid, respectively [35,39]. After the fungal attack, the area of the first band (7) increased for the HH and CG samples, while it decreased for all other samples. Similarly, the area of the second band (6) increased for the CH, HH, and CG samples, but decreased in all others.

The bands between 1250–1230 cm^-1^ correspond to C-O stretching vibrations of hemicellulose, lignin, and kinic acid (in CG samples) [39]. After the fungal attack, the area of this band (5) decreased for the all samples.

The weak shoulders observed between 1165–1155 cm^-1^ and 1120–1075 cm^-1^, along with the band observed between 1040–1020 cm^-1^, are attributed to C–O–C vibrations of cellulose and hemicellulose (glycosidic linkages), quinic acid (in the CG sample), and C–OH bending vibrations [33,34,40]. After fungal attack, the area of the first band (4) decreased in the WS and CG samples, while it increased in the other samples. The significant decrease in the band area of the CG sample may be associated with the removal of quinic acids from the structure. The area of the second band (3) decreased in the HB, BW, RD, WS, and CH samples, while it increased in the OW, HH, and CG samples. The area of the third band (2) decreased in the HH sample, whereas it increased in all other samples.

The weak bands observed between 900 and 870 cm^-1^ are attributed to C_1_–O–C_4_ β-(1→4) glycosidic linkages [40,41]. The area of these bands (1) increased in the CH, HH, and CG samples, while it decreased in all other samples.

*G. lucidum* was observed to exhibit degradative effects on lignocellulosic components, including cellulose, hemicellulose, lignin, and polysaccharides, as well as on chlorogenic acids present in spent coffee grounds. However, the extent of this degradation varied significantly depending on the substrate type. In particular, the degree of degradation in HH and CG substrates was considerably lower compared to the others, which likely hindered the biochemical environment required for fungal growth. Consequently, no mushroom development was observed on these substrates. In contrast, Akçay et al. [16] reported that *Pleurotus ostreatus*, unlike *G. lucidum*, was capable of degrading both HH and CG substrates, thereby enabling successful mushroom cultivation.

The FT-IR spectra (3600–800 cm^-1^) of *G. lucidum* mushrooms cultivated on substrates of hazelnut branches (HB), beech wood (BW), oak wood (OW), rhododendron branches (RD), corn husk (CH), and wheat straw (WS), along with the histograms of the peak areas obtained through Gaussian curve fitting applied to specific regions of these spectra, are presented in Fig. 9.

**Fig 9. FT-IR results.** a) FT-IR spectra of all mushroom samples; b) histograms of FT-IR peak areas processed using Gaussian curve fitting. HB-M: Mushroom fruit bodies cultivated from HB, other mushroom fruit bodies were coded similarly.

The broad bands observed between 3500 and 3000 cm^-1^ are attributed to O–H stretching vibrations due to intermolecular hydrogen bonds and humidity [42,43]. The band observed at 2912 and 2854 cm^-1^ are attributed to CH_2_ asymmetric and symmetric stretching vibrations of lipids in G. lucidum mushrooms. These bands are associated with the structural features of triterpenoids, which are characteristic of G. lucidum mushrooms [42,44]. Similarly, the two bands observed at 1455 and 1380 cm^-1^ are attributed to CH_3_ in-plane and symmetric bending vibrations in the aliphatic structure of triterpene compounds. While these vibrations are primarily attributed to the structural properties of triterpenoids, it has also been suggested that protein and polysaccharide structures can influence these bands [43]. Puliga et al [31] reported that CH_2_ bending vibrations in polysaccharides occur at 1455 cm^-1^, while Wickramasinghe et al. [42] mentioned that there was the presence of β-glucan in the band at 1373 cm^-1^. Analysis of the histograms corresponding to the bands (3, 4, 7) representing triterpene structures reveals that the peak area of the RD-M mushroom is greater than that of the other mushrooms.

The bands at 1635, 1535, and 1235 cm^-1^ are attributed to amide I (C=O stretching), amide II (C–N, N–H stretching), and amide III (C–N, N–H stretching) in the protein structure of G. lucidum mushrooms, respectively [31,44,45]. Analysis of the histograms corresponding to these bands indicates that the RD-M mushroom exhibits the highest peak areas for the first (6) and second (5) bands, whereas the WS-M mushroom shows the largest peak area for the third band (2) compared to the other mushrooms.

The bands observed at 1150 and 1037 cm^-1^ are attributed to C-O stretching vibrations of triterpene compounds and β-glucan of polysaccharides in the G. lucidum, as well as –OH and C–O–C stretching vibrations of sugar and polysaccharide structures (arabinan) [44]. Analysis of the histogram for the band at 1037 cm^-1^ (1) reveals that the OW-M and CH-M mushrooms exhibit higher peak areas compared to the other mushrooms. The weak band observed at 889 cm^-1^ is correspons to the anomeric configuration of polysaccharides. This band is associated to the presence of 1,3-β-D-glucan as well as C–H deformation in polysaccharide structures [31].

When all *G. lucidum* mushrooms are examined collectively, it is evident that lipid, protein, and polysaccharide components are generally present in the mushroom structure, regardless of the substrate type (HB, BW, RD, WS, OW, CH). However, evaluation of the peak areas in the FT-IR spectra, calculated using Gaussian curve fitting, reveals quantitative differences in these components depending on the substrate. It can be inferred that mushrooms cultivated on the RD substrate contain higher levels of triterpenes and proteins compared to those grown on other substrates, whereas mushrooms grown on OW and CH substrates exhibit higher polysaccharide content. In this study, G. lucidum mushrooms could not be cultivated on HH and CG substrates, whereas Akçay et al. [16] reported successful cultivation of Pleurotus ostreatus on both substrates.

Lines 203–216: Please remove this paragraph, as it does not belong in the Materials and Methods section. At most, it may be included at the end of the manuscript.

The paragraph was moved at the end of the manuscript after conclusion section, technic editor may decide exact place during the publication stage if accepted.

Results and Discussion – This section needs further development. The authors should discuss their findings in greater depth and include more references.

Line 227: “Some studies…” – Which studies? Please provide references.

Reference have been provided to the related sentence.

Lines 233–236: What explanation do the authors provide for this earlier onset of fruiting?

added: The shorter earliness in OW may reflect its optimal C/N ratio (50.68) and lignin content (∼25%), which enhance mycelial efficiency (See Table 3)

Lines 245–247: Beech sawdust is also widely used as a cultivation substrate.

Thank you for your suggestion, following sentence was revised and beech wood was added to the text

“In *G. lucidum* cultivation, beech wood (BW) and oak wood (OW) are widely recognized as the traditional basal substrates due to their optimal lignin content and C/N ratios (approximately 45~50) which are favorable fruiting body formation.”

Lines 261–262: This sentence should be moved to the Conclusions section.

Moved

Lines 290–291: What is the optimal C/N ratio for the growth and fruiting of G. lucidum? Lines 292–293: On what basis do the authors make this claim? Supporting literature anddiscussion are needed.

added: “The relationship between substrate C/N ratios and *G. lucidum* productivity is complex and substrate-dependent, though a clear positive correlation emerges across studies. While agricultural waste-based substrates like wheat straw and tea waste achieve peak yields at higher C/N ratios (70-80) due to their lignin-rich composition requiring extended carbon availability for degradation [10], wood-based substrates such as oak perform optimally at moderate ratios (~50) where nitrogen availability better supports mycelial expansion [24]. Our results align precisely with these mechanisms: low C/N substrates (e.g., coffee grounds at 8.6) failed to fruit entirely, while oak wood (C/N 50.7) yielded maximally (46 g/kg), corroborating Atila’s [28].) findings of yield being positively correlated with C/N (r=0.82, p<0.01) and negatively with nitrogen content. This pattern reflects *G. lucidum*’s dual metabolic demands – sufficient nitrogen for biomass accumulation balanced against carbon reserves for ligninolytic enzyme production during fruiting. Consequently, ideal C/N ranges must account for both substrate type (agricultural vs. woody) and cultivation phase (colonization vs. fruiting), with our 25-50 range accommodating this plasticity.”

Lines 305–311: The authors state that TPC values in mushrooms may vary depending on the solvent used for extraction. However, given the same methodology and solvent, how do they explain the significantly lower values reported here compared to those in the literature?

added: “This discrepancy can be attributed to several interconnected factors that collectively influence phenolic compound production and extraction efficiency. One of them is that the agricultural waste substrates used in our cultivation system appear to have preferentially stimulated polysaccharide biosynthesis over phenolic compound production, contrasting with the metabolic patterns observed in forest-grown specimens developing on natural woody substrates [2]. Another factor maybe related with tthe commercial harvesting protocol we followed, targeting optimal fruiting body size rather than peak metabolite accumulation, likely captured a different developmental stage than the mature specimens typically collected from wild populations [4]. Additionally, the domesticated strain utilized in this study, obtained from the Atatürk Horticultural Institute, may have been selectively bred for rapid growth characteristics rather than secondary metabolite production [6]. While Demirci et al. [30] have clearly established the significant impact of extraction solvents on measured TPC values, our findings further highlight how cultivation parameters - including substrate composition, genetic strain characteristics, and harvest timing - serve as equally critical determinants of phytochemical profiles”

Line 323: Insert the authority for Lentinus edodes.

Added authority for *Lentinus edodes*: “(Berk.) Pegler”

Table 4: The footnote is unclear and should be revised for clarity.

Table 4 was revised for clarity

Conclusions –Lines 492–495: This text should be rewritten in a more discursive manner.

added to the conclusion: “The findings of this study suggest several promising directions for future research on G. lucidum cultivation. Optimizing substrate mixtures, particularly combinations like oak wood and hazelnut branches, could significantly enhance both yield and cost-efficiency while utilizing agricultural byproducts more effectively. A deeper investigation into the enzymatic mechanisms governing G. lucidum's substrate preferences would provide valuable insights into its biodegradation capabilities, potentially enabling targeted improvements in cultivation practices. Finally, developing effective pretreatment methods for challenging waste materials such as hazelnut husks and spent coffee grounds could expand the range of viable substrates, further supporting sustainable mushroom production and waste valorization efforts”

Reviewer #4: Reviewer’s comments on PONE-D-25-12794R1Title: SuitableAbstract: Alright

Introduction: Line 58: Make it forest residue and not forestry residue.

done

Line 63: Please unbundle what you mean by energy or food.

added: biofuels, animal feed, or human food (e.g., mushrooms).

What do authors mean by biodegradation properties of G. lucidum?

added: Here, 'biodegradation properties' refer to *G. lucidum*'s capacity to decompose lignocellulosic components (cellulose, hemicellulose, lignin) quantified via FT-IR and chemical assays.

If the novelty statement of the manuscript suggests cultivation of the medicinal mushroom on lignocellulosic waste, authors should highlight in the last but one paragraph of the ‘Introduction’ section what substrates have been in use before now and why they think lignocellulosic substrates should replace them.

added: While prior studies used tea waste, sugarcane bagasse, or soybean meal [10, 12], we focus on regionally abundant wastes (hazelnut branches, rhododendron) to reduce disposal costs and enhance sustainability.

Materials and methods: Lines 103-105: Why the variations in substrate particle sizes?

This is the only answer not added to the text: The particle size of the substrate is determined by the form in which it is supplied and the maximum grinding capacity of the grinder. Nevertheless, having a range of particle sizes is beneficial for increasing surface area and porosity.

Lines 106-108: Please state what informed the substrate mixtures and/or cite an appropriate reference for it. What do authors mean by ‘standardized 91% substrate and 9% wheat bran? As far as I know, wheat bran is also a substrate which contains 46% of non-starch polysaccharides, including arabinoxylan (70%), cellulose (24%) and beta-glucan (6%), and it also contains minor amounts of glucoglucomannan and arabinogalactan (Carre and Brillouet, 1986). What is with wheat bran serving as a nitrogen source (supplementation)? Even if it can, it should not serve as a primary nitrogen source.

The reviewer’s comment is right but following references shows wheat bran can be used as N. The authors added reference no:11 to the related place.

in reference 11: Kurd-Anjaraki S, Ramezan D, Ramezani S, Samzadeh-Kermani A, Pirnia M, Shahi BY. Potential of waste reduction of agro-biomasses through Reishi medicinal mushroom (Ganoderma lucidum) production using different substrates and techniques. Acta Ecol Sin. 2022;42: 90–101. doi:10.1016/j.chnaes.2021.04.010.

page 99

“The carbon of the substrate is mainly supplied by various lignocellulosic compounds, and the N is mainly provided by various supplements such as wheat bran [[50](https://www.sciencedirect.com/science/article/pii/S1872203221000548?casa_token=aArUZCt20O8AAAAA:OTdSuD5fluMLqgeoz1Kto0flYaRSyD-gAZxsSH-x1Fj8kLDzsOpzwx0zdi_SY7pwTx-rjks4uQ#bb0250)]. Each fungus variety needs optimal amounts of C/N ratio in the substrate to achieve optimal growth and produce the highest yield in the shortest time [[11](https://www.sciencedirect.com/science/article/pii/S1872203221000548?casa_token=aArUZCt20O8AAAAA:OTdSuD5fluMLqgeoz1Kto0flYaRSyD-gAZxsSH-x1Fj8kLDzsOpzwx0zdi_SY7pwTx-rjks4uQ#bb0055)] while the values of C/N of PWC in present study was the higher than standards.”

in: S.A. Dowom, S. Rezaeian, H.R. Pourianfar, Agronomic and environmental factors affecting cultivation of the winter mushroom or Enokitake: achievements and prospects, Appl. Microbiol. Biotechnol. 103 (6) (2019) 2469–2481, https://doi.org/10. 1007/s00253-019-09652-y.

page 2472 title : Nitrogen content of substrate/media and its interaction with carbon

“While carbon is provided by basic lignocellulosic residues, nitrogen content of a substrate is mostly determined by supplementations added to it, including wheat bran, sugarcane bagasse, and rice bran”

10% wheat bran suplementation was also mentioned in Table 1 in same reference (page 2471)

Following sentence was revised as follow in the manuscript

“Control groups utilized a standardized substrate composed of 91% substrate + 9% wheat bran (containing of 1.88 % N content) [10, 11], with wheat bran serving as an optimal nitrogen supplement to balance the C/N ratio”

Line 118: To what level was the pH adjusted?

The pH levels of the substrates after the addition of CaCO₃ are shown in Table 6.

What was the 90 minutes autoclaving for? How did authors arrive at that?

In mushroom cultivation, autoclaving is generally applied for 90 minutes. A reference was added to the related place. (Atila F, Ogutcu H, Bilginoglu E, Kazankaya A, Kumar P, Fayssal SA. Effect of Phenolic-rich Forest and Agri-food Wastes on Yield, Antioxidant, and Antimicrobial Activities of Ganoderma lucidum. Biomass Convers Biorefinery. 2023;14: 25811–25821. doi:10.1007/s13399-023-04708-6)

Please remove all horizontal and vertical lines from Table 1 except the top two horizontal lines for headers and bottom horizontal line for closure.

Thank you for your suggestion; it would indeed be an improvement. However, the journal's writing guidelines allow the use of both horizontal and vertical lines. The technical editor will make the final decision regarding the formatting

Line 129: please make the phrase ‘mixed homogeneously’ read ‘mixed to homogeneity’.

done

Lines 132-133: Cite reference.

cited

Line 136: How did authors raise the incubation to a range between 28 and 30C? Please, pick a temperature value to which the preparation was incubated and why. Cite a reference.

References were added to the related places.

Results: Please remove all horizontal and vertical lines from all tables as earlier recommended.

Thank you for your recommendation; it would indeed be an improvement. However, the journal's writing guidelines allow the use of both horizontal and vertical lines. The technical editor will make the final decision regarding the formatting

What is the relationship between alpha-cellulose and hemicellulose?

In our study, α-cellulose increased proportionally after-cultivation due to preferential hemicellulose degradation by fungal enzymes. Hemicellulose degradation provides readily metabolizable pentoses (xylose, arabinose) that support initial mycelial growth, while cellulose breakdown is reserved for later fruiting body development.
